# Anisotropic polymer nanoparticles with controlled dimensions from the morphological transformation of isotropic seeds

**DOI:** 10.1038/s41467-019-13263-6

**Published:** 2019-11-27

**Authors:** Zan Hua, Joseph R. Jones, Marjolaine Thomas, Maria C. Arno, Anton Souslov, Thomas R. Wilks, Rachel K. O’Reilly

**Affiliations:** 1 Department of Chemistry, University of Warwick, Gibbet Hill Road, Coventry, CV4 7AL UK; 2 School of Chemistry, University of Birmingham, Edgbaston, Birmingham B15 2TT UK; 3 Department of Physics, University of Bath, Claverton Down, Bath, BA2 7AY UK

**Keywords:** Polymers, Polymer synthesis

## Abstract

Understanding and controlling self-assembly processes at multiple length scales is vital if we are to design and create advanced materials. In particular, our ability to organise matter on the nanoscale has advanced considerably, but still lags far behind our skill in manipulating individual molecules. New tools allowing controlled nanoscale assembly are sorely needed, as well as the physical understanding of how they work. Here, we report such a method for the production of highly anisotropic nanoparticles with controlled dimensions based on a morphological transformation process (MORPH, for short) driven by the formation of supramolecular bonds. We present a minimal physical model for MORPH that suggests a general mechanism which is potentially applicable to a large number of polymer/nanoparticle systems. We envision MORPH becoming a valuable tool for controlling nanoscale self-assembly, and for the production of functional nanostructures for diverse applications.

## Abstract (web summary)

Understanding and controlling self-assembly processes at multiple length scales is essential to design and create advanced materials. Here the authors report a method for the production of highly anisotropic nanoparticles with controlled dimensions based on the morphological transformation of initially isotropic seeds, driven by supramolecular bonding.

## Introduction

Complex structures are a hallmark of natural systems, achieved through hierarchical self-assembly across multiple length scales—bone, with its remarkable combination of stiffness and toughness, exhibits ordering across at least nine distinct levels, from the molecule upwards^1^. Scientists have long been interested in mimicking biological organisation to create artificial materials with similarly exceptional properties. However, while chemists have become adept at manipulating molecules, it has been more challenging to achieve the same degree of control at the nanoscale, just one level up. In particular, highly anisotropic structures are a natural target for nanoscale self-assembly because they are ubiquitous in biology (e.g. microtubules, muscle filaments) and have been shown to possess unique properties in applications as diverse as photonics^2–4^ and drug delivery^5–10^. The ideal building blocks for the bottom-up self-assembly of anisotropic nanostructures would (1) be readily accessible (i.e. cheap and scalable syntheses), (2) allow straightforward tuning of chemical composition (so materials can be tailored for different applications) and (3) allow fine control over nanoparticle dimensions (enabling controlled higher-order assembly at still larger length scales). However, combining all three of these requirements has proven difficult.

DNA nanotechnology^11–13^ allows well-defined anisotropic nanostructures to be built with a high degree of precision, but concerns remain about the scalability and cost-effectiveness of this approach (despite recent advances^14^). Inorganic nanoparticles are more accessible, and high aspect ratio structures can be produced, but only limited variation of the chemical composition is possible^3,4^. In this context, synthetic polymers are highly promising building blocks because their synthesis is cheap and scalable, and the development of controlled polymerisation techniques has allowed straightforward modulation of the length, architecture and chemical composition of polymer chains^15^.

However, while the self-assembly of polymers in solution affords bottom-up access to nanoscale objects^16^, controlling this process to make highly anisotropic nanoparticles with well-defined dimensions has proven to be very challenging. This is because conventional methods rely on exploiting differences in the stabilities of particles with different shapes, which are not significant between high aspect ratio structures of different lengths^17^. While a large body of work exists demonstrating the formation of anisotropic polymer nanostructures^8,10,16,18,19^, and even triggered switching between different shapes^20,21^, it remains challenging to create stable systems with fine control of nanoparticle dimensions. For example, it is possible to generate pure phases of wormlike nanoparticles using polymerisation-induced self-assembly^18^, but the products are highly disperse with little control over length and width.

**Fig. 1:**
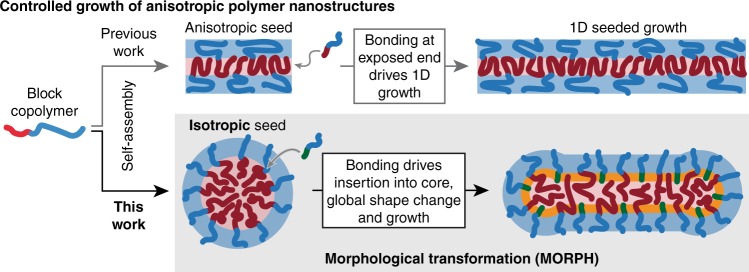
Methods for the growth of anisotropic polymer nanostructures with controlled dimensions. Previous efforts have been based on 1-dimensional growth from an anisotropic seed particle (top). We report a qualitatively different process based on the global morphological transformation of an isotropic seed particle (bottom).

The seeded growth method (Fig. 1) circumvents these problems, but is somewhat limited in scope. In the seeded growth approach, anisotropic nanoparticle seeds are fed with polymer unimers in solution and 1-dimensional (1D) growth is driven by the formation of bonding interactions between the unimers and the exposed ends of the seed. Under the right conditions, this enables the growth of long, cylindrical particles with lengths determined simply by the amount of added unimer. Crystallisation-driven self-assembly (CDSA)^22–25^ is the most well-known example, but metal–metal interactions^26,27^, hydrogen bonding (H-bonding)^28^ and π–π stacking^29^ have also been exploited. However, seeded growth has so far proven limited to a narrow range of polymers and depends on the generation of a uniform population of anisotropic seed particles, which can be a major challenge.

Here, we report a distinct method for the production of high aspect ratio anisotropic polymer nanoparticles with controlled lengths and widths, which we term morphological transformation (MORPH, Fig. 1). Unlike 1D seeded growth, MORPH uses readily accessed isotropic nanoparticles as a starting point, which are then driven to transform and grow into anisotropic structures by the formation of supramolecular bonds with an added polymer. We show that the amount of growth is determined by the amount of added polymer, allowing fine control over length. We further demonstrate that multi-functional nanoparticles of defined dimensions can be built using MORPH by simple stepwise growth using appropriately modified polymers. Finally, we develop a physical model for the process, which suggests that the method may be generalisable to a broad range of polymer systems and supramolecular interactions, and with many potential applications across materials science.

## Results

### Single-step MORPH

We first encountered the possibility for MORPH while investigating the self-assembly behaviour of nucleobase-containing polymers^30–36^, in a nanoparticle system that exhibited poorly controlled shape-changing behaviour in response to the addition of a polymer in solution. A handful of other examples of this behaviour were subsequently found in the literature^37,38^, which had similarly failed to demonstrate control of product dimensions. We reasoned that achieving this control would result in a useful approach to the generation of well-defined anisotropic nanoparticles and hypothesised that these previous efforts had not succeeded because of nanoparticle disassembly/reassembly during the transformation process, which led to the formation of a range of structures of different sizes. To retain control, it would be necessary to eliminate this pathway, and we hypothesised that this could be achieved by building the seed nanoparticle out of a polymer with low water solubility and a high glass transition temperature (*T*_g_). This would inhibit disassembly by making extraction of polymer chains into the bulk solvent highly unfavourable and introducing a significant barrier to rearrangement of the nanoparticle core.

**Fig. 2:**
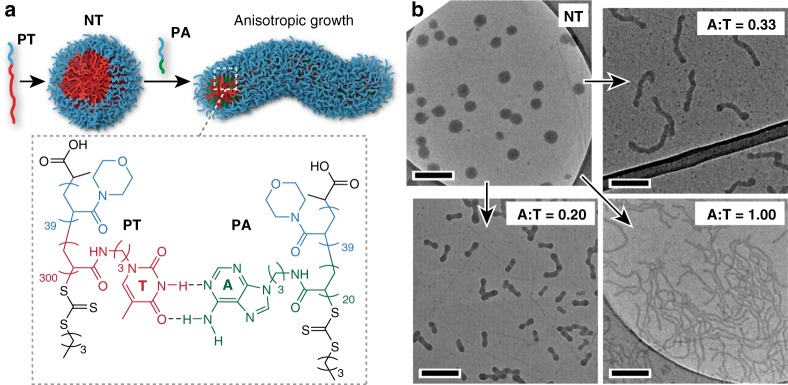
Single-step transformation of spherical nanoparticles into different anisotropic morphologies by MORPH. **a** Schematic representation of MORPH using nucleobase-containing polymers. Spherical nanoparticle **NT** with a thymine-containing core was formed by self-assembly of the polymer **PT** using a solvent switch method from DMF to water. Introduction of the adenine-containing polymer **PA** induced MORPH at A:T molar ratios above 0.20. **b** TEM images of the nanoparticles formed before and after addition of **PA** at different A:T molar ratios to separate solutions of **NT**. Particles were imaged stain-free on graphene oxide (GO)^46^. Scale bars = 200 nm.

To test these hypotheses, we synthesised the amphiphilic block copolymer **PT** with a long, hydrophobic thymine-containing block and a short hydrophilic block (see Supplementary Methods). This design was intended to minimise water-solubility and maximise the polymer’s *T*_g_, which was measured to be 73 °C. **PT** was self-assembled in water to give well-defined nanoparticle **NT** (Fig. 2a, and Supplementary Fig. 9) with a diameter of ~60 nm according to transmission electron microscopy (TEM) (Fig. 2b). A second polymer, **PA** (see Supplementary Methods), was designed with the same length of hydrophilic block as **PT** and an adenine-containing block that was sufficiently short to allow **PA** to freely dissolve in water at moderate concentrations. **PA** was added at different molar equivalents to separate solutions of **NT**, and the mixtures stirred for 2 h at 24 °C. Stain-free TEM analysis revealed that addition of **PA** to **NT** drove a transformation from spheres to dumbbells or worms depending on the A:T molar ratio (Fig. 2b and Supplementary Fig. 11).

### Controlled stepwise MORPH

**Fig. 3:**
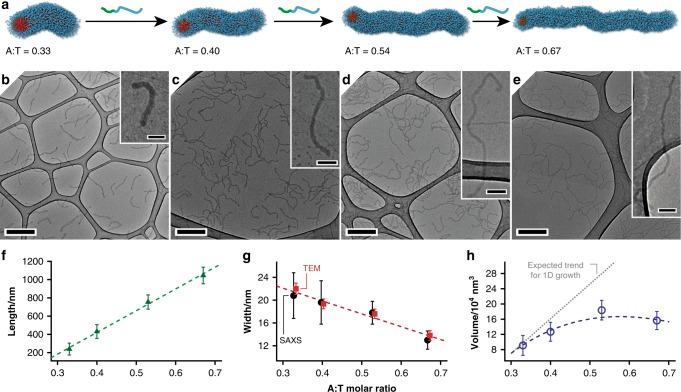
Stepwise growth of well-defined anisotropic nanoparticles by MORPH. **a** Schematic representation and **b**–**h** characterisation of the wormlike nanoparticles. **b**–**e** Stain-free TEM images of the nanoparticles at different A:T molar ratios; the scale bars in (**b**–**e**) and their insets are 500 and 100 nm, respectively. Average lengths (**f**), widths (**g**) and volumes (**h**) of the worms from analyses of TEM images (**b**–**e**) and SAXS. Note that the TEM and SAXS data in **g** have been offset for clarity. Error bars represent one standard deviation.

We next explored whether sequential addition of **PA** could achieve the same behaviour, by feeding **NT** with small aliquots (0.07 molar equivalents) of **PA**, leaving 2 h between additions. Surprisingly, this did not lead to the same MORPH process. Instead, the particles remained spherical, swelling in size before disassembling into much smaller spherical particles as the A:T ratio approached 1:1 (Supplementary Fig. 14). We speculated that a threshold concentration of **PA** was required to induce anisotropy, after which stepwise growth of the worms might be possible. To explore this idea, short seed worms ~300 nm long were fabricated by adding **PA** to **NT** at an A:T molar ratio of 0.33 (Fig. 3a), followed by stepwise addition of further **PA**. Figure 3b–e shows that longer and thinner worms were obtained with each addition of **PA**. The average contour length gradually extended to over 1000 nm, with an approximately linear relationship between worm length and A:T molar ratio, and narrow length distributions (Fig. 3f and Supplementary Figs. 16, 17). TEM images suggested the average width of the worms decreased from ~22 nm to 14 nm, with a slight increase in the volumes of individual worms (Fig. 3g, h). The decrease in width was verified for the bulk sample by small-angle X-ray scattering (SAXS) analyses (Fig. 3g and Supplementary Fig. 18). These results confirmed that the growth process could be controlled, and that uniformly sized wormlike nanoparticles could be produced using this method.

### MORPH control experiments

We performed a series of control experiments in which H-bonding for one of the polymers was blocked or removed altogether (see Supplementary Discussion and Supplementary Figs. 20–25). In no case was MORPH observed, confirming that the process was driven by the formation of strong H-bonding interactions as opposed to weaker hydrophobic effects. We also investigated whether the process was indeed a single-particle transformation, rather than particle–particle fusion mediated by **PA**, by obtaining further information about the mass average molar masses ($\overline{M_{\mathrm{{w}}}}$) of **NT** and the dumbbells formed on addition of **PA** (A:T = 0.20) using light scattering (LS). The dumbbells were found to have a $\overline{M_{\mathrm{{w}}}}$ of 69 ± 1 × 10^6^ Da, indicating an average mass increase of only 8% compared with **NT** ($\overline{M_{\mathrm{{w}}}}$ = 63 ± 1 × 10^6^ Da) (Supplementary Figs. 28, 29), consistent only with a single-particle transformation process. Finally, we asked whether the adoption of an anisotropic shape might be due to polymer crystallisation, since this is well known to favour 1D structures^22^. Attempts to perform wide-angle X-ray scattering on samples of worms at an A:T ratio of 0.67 in solution were unsuccessful as the scattering from the sample solution was indistinguishable from that of the solvent—even at high concentrations—so we investigated the same nanoparticles using micro-differential scanning calorimetry (Supplementary Fig. 27). The absence of any significant thermal events across the temperature range studied led us to conclude that the sample was most likely amorphous, and that crystallinity was unlikely to be the driving force behind the adoption of an anisotropic shape.

### Tuning polymer block lengths to influence MORPH

The use of polymers for the construction of the nanoparticles gives us a straightforward method for investigating how perturbations of the system affect the MORPH process. We synthesised different versions of **PA** in which we varied the lengths of the adenine or hydrophilic block (Supplementary Discussion and Supplementary Figs. 31, 32). Increasing the length of the hydrophilic block (from DP 39 to 96 or 295) resulted in drastic changes to the MORPH process. While for the original **PA** disassembly to small spheres was only observed at A:T ratios above 1:1, increasing the hydrophilic block length caused disassembly to occur at lower ratios (Supplementary Fig. 31). Decreasing the length of the adenine block (while retaining the same hydrophilic block length as in the original **PA**), meanwhile, abolished the development of anisotropy completely, with disassembly to small spheres observed at all A:T ratios studied. In contrast, increasing the length of the adenine block resulted in a slower progression along the MORPH pathway, with dumbbells observed at an A:T ratio of 0.33 (compared to 0.20 for the original **PA** sample) and the growth of noticeably shorter, fatter worms when compared to those produced using the original **PA** sample at the same A:T ratio (Supplementary Fig. 32).

### Controlled growth of multifunctional worms using MORPH

**Fig. 4:**
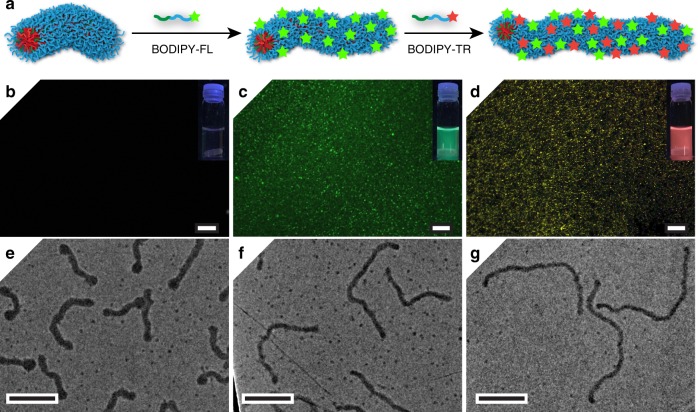
Controlled fabrication of fluorescent anisotropic nanoparticles by MORPH. **a** Non-fluorescent seed worms with A:T molar ratio 0.33 were converted into green and then yellow fluorescent nanoparticles by adding BODIPY-FL (**PA**^**G**^) and -TR (**PA**^**R**^) tagged polymers sequentially. **b**–**d** Confocal fluorescence images (overlay of green and red channels, scale bars = 10 μm) with photographs of the nanoparticle solutions under UV light as insets. **e**–**g** Stain-free TEM images confirming the controlled morphological transformation (scale bars = 200 nm).

To illustrate the potential to use MORPH to produce well-defined, functional nanoparticles, **PA** was derivatised with two different dyes—BODIPY-FL (**PA**^**G**^) and -TR (**PA**^**R**^) (Supplementary Discussion and Supplementary Figs. 33, 34)—and used in worm growth experiments. Starting with unfunctional worms at an A:T ratio of 0.33, we added 0.07 equivalents of **PA**^**G**^ followed by 0.07 equivalents of **PA**^**R**^ and inspected the resulting worms using TEM and confocal fluorescence microscopy (Fig. 4). As expected, feeding the unfunctional worms with **PA**^**G**^ gave green fluorescent worms (Fig. 4, middle column). Further feeding with **PA**^**R**^ gave yellow fluorescent worms as a result of colocalisation of the dyes (Fig. 4, right-hand column—see Supplementary Fig. 35 for control experiments). Control over worm dimensions was retained, as evidenced by stain-free TEM images (Fig. 4, bottom row).

## Discussion

MORPH operates differently from 1D seeded growth—as illustrated in Fig. 3f–h, an increase in worm length is accompanied by a corresponding decrease in width. Whereas in 1D seeded-growth nanoparticles lengthen only at the ends, the worms obtained using MORPH grow along their full length. This is because the complementary copolymers can insert themselves anywhere along the worm, as confirmed by the LS and TEM data showing even widths along the full lengths of the nanostructures. Addition of further polymer triggers a global transformation, as opposed to local growth at the nanoparticle ends. MORPH’s other unique feature is induced anisotropy, which allows readily accessed isotropic particles to be used as seeds. In the system we describe, the MORPH process is highly controlled, reproducibly giving wormlike nanoparticles with well-defined dimensions determined simply by the amount of added complementary copolymer. The growing particles retain their reactivity and remain capable of incorporating more of the complementary polymer as they grow. As a result, worm length scales approximately linearly with the amount of added copolymer, allowing specific particle dimensions to be targeted.

To confirm which features of our system are necessary for MORPH to occur, we begin by considering the behaviour of the polymer chains in the cores of the nanoparticles. The glass transition temperature (*T*_g_) of **PT** was found to be 73 °C, far above the experimental temperature (24 °C), indicating that the core of **NT** is most likely in a ‘frozen’, glassy state, with low chain mobility^39^. Bulk *T*_g_ does not always give a reliable indication of the behaviour of a polymer in a solvated nanoparticle^40^, but other evidence also points to glassy core dynamics: when we mixed **PA** and **PT** in the appropriate ratios in DMF (a good solvent for all blocks) and performed a slow solvent switch to water, only spheres were observed (Supplementary Fig. 26), which indicates that these are the thermodynamically most stable structures^39^. This observation implies that in the worm-like nanoparticles formed, there is a significant barrier to core chain mobility, and these glassy dynamics are essential to preserve the anisotropic shape and prevent disassembly into spheres. Because core chain mobility is restricted in **NT**, there must be a driving force behind MORPH—in the system we consider, this is the formation of energetically favourable H-bonds between thymine and adenine. The absence of any rearrangement driven solely by the hydrophobic effect (see Supplementary Figs. 20–25) further implies that this driving force must exceed a threshold value. Below, we show that MORPH can be explained on the basis of these properties (glassy core dynamics and the presence of a driving force for polymer insertion), without the need to invoke more specific details of the chemical bonding involved, such as directionality.

**Fig. 5:**
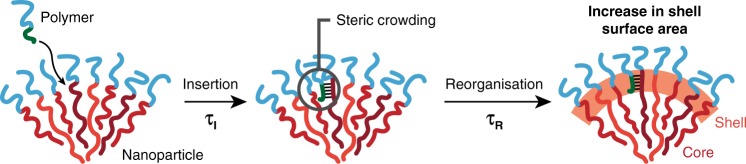
Illustration of the polymer insertion process in MORPH. The polymer inserts with timescale *τ*_I_ into a thin shell region at the core–corona interface. Insertion causes steric crowding that must be relieved by reorganisation to increase the shell’s surface area, which proceeds with timescale *τ*_R_.

To develop a physical model for the process, we consider the response of a nanoparticle to the insertion of a polymer at the core–corona interface (Fig. 5). We assume that the polymer will diffuse to the interface and insert into the nanoparticle core with an associated timescale, *τ*_I_, but that because of the short length of the core block in the added polymer this insertion will occur only in a thin shell region. It is predicted that *τ*_I_ will be inversely proportional to the concentration of the polymer (i.e., more polymer will lead to faster insertion). Insertion of the polymer will introduce steric crowding which the system can relieve by two possible routes. The first, which is thought to be operational in many copolymer systems capable of undergoing morphological transitions^41,42^, is removal of material from the nanoparticle by extraction of polymer chains into solution. However, in MORPH, this process is strongly disfavoured because of a combination of the glassy core dynamics and strong bonding between the nanoparticle and inserted polymer. With chain extraction suppressed, the only way that the additional mass can be accommodated is by an increase in the surface area of the core–corona interface, requiring rearrangement of the nanoparticle core chains, with timescale *τ*_R_. We assume that *τ*_R_ will be determined principally by the bulk properties of the core chains (i.e. the bulk modulus and viscosity) and remain more or less independent of the polymer concentration.

**Fig. 6:**
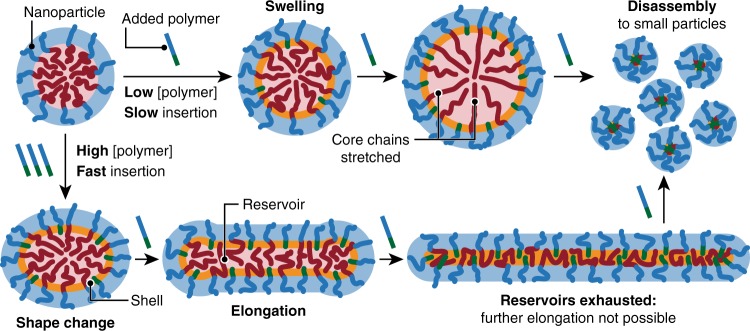
Proposed MORPH pathways. Schematic illustrations at low (upper pathway) and high (lower pathway) concentrations of the added polymer.

Since the sphere is the shape with the least surface area per unit volume, an increase in the surface area of the nanoparticle’s core–corona interface can be achieved in only two ways: swelling or shape change. At sufficiently low polymer concentrations, *τ*_I_^−1^ < *τ*_R_^−1^ and insertion of the polymer is slow enough that the core chains have time to rearrange and allow expansion of the interface by simple isotropic swelling, minimising the core surface-tension energy (Fig. 6, upper pathway)^41^. Swelling requires stretching of the core polymer chains, so it cannot continue indefinitely—at some point no further stretching is possible and the only way for the system to continue increasing the shell surface area to accommodate more of the added polymer is by disassembly into small particles, in agreement with the experimental observations (Supplementary Fig. 14). At higher polymer concentrations, *τ*_I_^−1^ > *τ*_R_^−1^ and rapid insertion of the polymer requires an equally fast increase in the surface area of the interface, which can only be achieved by both core chain rearrangement and shape change (Fig. 6, lower pathway). Induction of anisotropy is only possible if the sphere cannot isotropically swell on the timescale of polymer insertion, and proceeds by overcoming the core surface-tension energy barrier that favours a spherical shape. Once this barrier has been overcome and anisotropy induced, elongation is expected to become the favoured pathway because, unlike swelling, it does not require the energetically unfavourable stretching of core chains. Regardless of the polymer concentration, further insertion is therefore anticipated to cause the dumbbell to increase in length and shrink in width as more material is drawn from the core to form bonds with the added polymer in the shell region. Each nanoparticle core can be thought of as a reservoir of material capable of driving elongation by forming bonds with the added polymer. When the core is saturated with bonds to the added polymer, this reservoir is exhausted, and elongation is no longer possible. Addition of further polymer is then expected to drive disassembly, as for the low concentration swelling pathway, and this was indeed observed in the experimental system at A:T ratios above around 1:1 (Supplementary Fig. 19).

We can formalise this physical argument by writing down the following equation for the evolution of small shape eccentricity, *ϵ* (i.e., a parameter to represent particle anisotropy): ∂_t_*ϵ*^2^=(*τ*_I_^−1^−*τ*_R_^−1^) (see Supplementary Discussion for a phenomenological derivation). When *τ*_I_^−1^ < *τ*_R_^−1^, *ϵ* = 0 is a stable solution of the equation, indicating isotropic growth due to minimisation of core surface-tension; when *τ*_I_^−1^ > *τ*_R_^−1^, *ϵ* ≠ 0 and eccentricity grows in time, indicating the induction of anisotropy. Note that unlike many instabilities in equilibrium polymer micelles^43–45^, the glassy core prevents the anisotropic nanoparticles from disassembling in the absence of an external source of added polymer. This model predicts that MORPH proceeds through an ellipsoidal intermediate, so we attempted to access this in the experimental system by forming particles at an A:T ratio intermediate between isotropic swelling (A:T = 0.07) and dumbbell formation (A:T = 0.20). As predicted, LS and SAXS analyses confirmed the formation of prolate ellipsoids at an A:T molar ratio of 0.14 (Supplementary Fig. 13). The model also explains the observed effects of varying the core and corona block lengths (Supplementary Figs. 31, 32). Increasing the length of the corona block in **PA** is predicted to result in more mass being added into the nanoparticle corona per adenine residue, in turn requiring a greater degree of structural reorganisation and therefore faster progression along the MORPH pathway. Decreasing the length of the adenine block is predicted to have a similar effect because this also results in more coronal mass being added per adenine residue—it is interesting to note that our experiments indicate that changing the core block length seems to have a much more drastic impact on the process than changing the corona block length. Decreasing the amount of coronal mass added per adenine unit—which we achieved above by increasing the length of the adenine block in **PA**—is by contrast expected to result in a lower degree of structural reorganisation and therefore slower progression along the MORPH pathway, and this was indeed observed. This highlights the potential to use MORPH to tune nanoparticle dimensions by making straightforward adjustments to the polymers involved.

Our minimal complexity model therefore succeeds in describing the main features of the MORPH process. It assumes only that (1) the seed nanoparticle has a glassy core, (2) the added polymer is sufficiently short to penetrate only a thin shell around the core, and (3) its insertion results in the formation of reasonably strong bonds. A wide range of polymers assemble into nanoparticles with glassy core dynamics, controlled polymerisation techniques allow polymer length to be easily tuned, and supramolecular chemistry provides us with a broad palette of strong, non-covalent bonding interactions. We therefore hypothesise that MORPH could be a general mechanism for the generation of anisotropy applicable to a large number of polymer/nanoparticle systems beyond the specific case reported here.

We report a distinct method, MORPH, for the production of anisotropic polymer nanoparticles with well-defined aspect ratios, based on the morphological transformation of isotropic seeds driven by the formation of supramolecular bonds. MORPH exhibits a number of useful features: the isotropic seed nanoparticles are straightforward to assemble because they are the thermodynamic product of a simple self-assembly process; the shape change can be controlled with precision simply by mixing aqueous solutions of nanoparticles and complementary polymer; and the global transformation process makes it straightforward to incorporate additional functionality across the nanostructure, as demonstrated by our stepwise incorporation of fluorescent tags. One can imagine incorporating functional ligands, therapeutics and reporter groups to build up multifaceted delivery platforms with a high degree of control. In future work, particles like these could be used to explore the precise effects of aspect ratio on their interactions with cells. Most significantly, the physical model we have developed suggests that it may be possible to exploit the huge diversity of supramolecular bonding motifs to perform controlled nanoparticle growth using MORPH and could, therefore, become an invaluable addition to the self-assembly toolkit.

## Methods

### Polymer synthesis

The adenine- and thymine-functionalised monomers (**AAm** and **TAm**) were synthesised as reported in our previous work^33^ and full details are given in the Supplementary Methods. The poly(4-acryloylmorpholine) (**PNAM**_**39**_) macro-chain transfer agent (macro-CTA) was synthesised by RAFT polymerisation as follows. A 10 mL ampoule was charged with NAM (500 µL, 4.0 mmol), 2-(((butylthio)carbonothiolyl)thio)-propanoic acid (23.8 mg, 0.1 mmol), VA-044 (1.3 mg, 0.004 mmol) and a mixture of 1,4-dioxane and water (2.0 mL, v:v 1:4). The mixture was thoroughly degassed via 4 freeze–pump–thaw cycles, filled with nitrogen and then immersed in an oil bath at 70 °C for 2 h. The polymerisation solution was precipitated three times from cold CH_3_OH. The light yellow polymer was dried in a vacuum oven overnight at room temperature and analysed by ^1^H NMR spectroscopy and size exclusion chromatography (SEC) using N,N-dimethylformamide (DMF) as an eluent. The degree of polymerisation (DP) was calculated using ^1^H NMR spectroscopy by comparing the integrated signals corresponding to the backbone signals (*δ* = 1.62 ppm) with those of the methyl group from the CTA (*δ* = 0.87 ppm). **PNAM**_**39**_ was then chain extended to give the diblock copolymers used in this report—an example procedure for **PT** follows. **PNAM**_**39**_ (14 mg, 0.0025 mmol), **TAm** (178 mg, 0.75 mmol) and the initiator VA-044 (0.08 mg, 0.00025 mmol) were dissolved in a mixture of DMF and water (0.5 mL, v:v 1:1). The mixture was thoroughly degassed via 4 freeze–pump–thaw cycles, filled with nitrogen and then immersed in an oil bath at 70 °C overnight. An aliquot of the crude product was taken and analysed by ^1^H NMR spectroscopy to calculate the conversion and degree of polymerisation. The residual solution was then precipitated three times from cold CH_3_OH. The light yellow polymer was dried in a vacuum oven overnight at room temperature and analysed by ^1^H NMR spectroscopy and DMF SEC.

### Self-assembly of PT to form NT

The seed nanoparticles **NT** were assembled as follows. **PT** was dissolved in DMF (at 8 mg mL^−1^) and stirred for 2 h at 70 °C. Then an excess of 18.2 MΩ cm water was added via a syringe pump at a rate of 1 mL h^−1^. The final volume ratio between water and organic solvent was about 8:1. The solution was then dialysed against 18.2 MΩ cm water, incorporating at least six water changes, to afford nanoparticles **NT** at a concentration of ca. 1 mg mL^−1^.

### MORPH of NT

For the single-step transformations shown in Fig. 2, **PA** was dispersed in H_2_O at 5 mg mL^−1^. This was then added to separate solutions of the nanoparticle **NT** (0.5 mg mL^−1^) with stirring at A:T molar ratios of 0.07, 0.20, 0.33, 0.67, 1.0, 1.33. The molar ratios were calculated according to the *M*_n_ determined from ^1^H NMR spectroscopic analyses and the polymers’ mass concentrations. The mixtures were sealed and allowed to stir at room temperature for 2 h, and then characterised by LS and TEM. For the stepwise growth experiments shown in Fig. 3, **PA** (0.33 molar ratio of A relative to T) was added to a solution of **NT** (0.5 mg mL^−1^) to give short seed worms. After 2 h stirring at 24 °C, further **PA** solution (0.07 molar ratio of A relative to T) was added. This process was repeated until A:T molar ratios of 0.33, 0.40, 0.53 and 0.67 were achieved. Each stage was characterised by TEM and SAXS analyses.

### Controlled production of fluorescent wormlike nanoparticles

**PA** was modified with BODIPY-FL or BODIPY-TR as described in the Supplementary Discussion to give **PA**^**G**^ and **PA**^**R**^, respectively. A solution of **NT** (0.5 mg mL^−1^) was then fed with an aliquot of **PA** solution to give non-fluorescent wormlike nanoparticles with an A:T molar ratio of 0.33. This solution was then fed with an aliquot of **PA**^**G**^ followed by an aliquot of **PA**^**R**^ (0.07 molar equivalents of A relative to T in both cases), with 2 h stirring at 24 °C in between each addition. Each stage was characterised by TEM and confocal fluorescence microscopy. In addition, control experiments were performed in which the seed worms were fed with two successive additions of the same dye-functionalised polymer to give pure red or green worms.

### Light scattering and SAXS experiments

Scattering experiments were conducted to determine the uniformity of particles within the bulk sample. Initial estimates for hydrodynamic diameters and the particle size distribution were made using a Malvern Zetasizer Nano S instrument fitted with a (He–Ne) 633 nm laser module and the refractive index increment was measured using a DnDc1260 differential refractometer supplied by PSS GmbH. The mass average molar mass and particle size information were then more accurately determined using an ALV-CGS3 goniometer-based system supplied by ALV GmbH, also operating at *λ* = 633 nm.

SAXS measurements were made using a Xenocs Xeuss 2.0 equipped with a micro-focus Cu Kα source collimated with Scatterless slits. The scattering was measured using a Pilatus 300k detector with a pixel size of 0.172 mm × 0.172 mm. A radial integration as a function of scattering length, *q*, was performed on the 2-dimensional scattering profile and the resulting data corrected for absorption and background from the sample holder.

## Supplementary information

Supplementary Information

## Data Availability

The authors declare that the data supporting the findings of this study are available within the paper and its supplementary information file. Raw data underlying the figures presented are available from the corresponding authors upon reasonable request.
