# Supplementary material for: Anisotropic polymer nanoparticles with controlled dimensions from the morphological transformation of isotropic seeds
Source: Nat Commun. 2019 Nov 27;10:5406. doi: 10.1038/s41467-019-13263-6 (PMC6881314; doi:10.1038/s41467-019-13263-6)
Supplement: Supplementary file 1 — Supplementary Information [file 41467_2019_13263_MOESM1_ESM.pdf]

Supporting Information

for

Anisotropic polymer nanoparticles with controlled dimensions from the morphological transformation of isotropic seeds

Zan Hua[1, 2], Joseph R. Jones[2], Marjolaine Thomas[2], Maria C. Arno[2], Anton Souslov[3], Thomas R. Wilks[2] and Rachel K. O'Reilly[2]**

[1]Department of Chemistry, University of Warwick, Gibbet Hill Road, Coventry, CV4 7AL, UK.

[2]School of Chemistry, University of Birmingham, Edgbaston, Birmingham, B15 2TT, UK.

[3]Department of Physics, University of Bath, Claverton Down, Bath BA2 7AY, UK.

*Corresponding authors: Thomas R. Wilks (t.r.wilks@bham.ac.uk) and Rachel K. O'Reilly (r.oreilly@bham.ac.uk)

TABLE OF CONTENTS

SUPPLEMENTARY METHODS **5**

Materials 5

Instrumentation & Analysis 6

NMR Spectroscopy *6*

Size Exclusion Chromatography (SEC) *6*

Refractive Index (RI) Measurements *6*

Dynamic Light Scattering (DLS) Analysis *7*

Static and Dynamic Light Scattering (LS) Analysis *7*

Small-Angle X-Ray Scattering (SAXS) Analysis *9*

Transmission Electron Microscopy (TEM) *9*

Atomic Force Microscopy (AFM) *10*

Confocal Microscopy *10*

Micro-Differential Scanning Calorimetry (microDSC) *10*

Monomer Syntheses 11

Synthesis of N-(3-bromopropyl)acrylamide *11*

Synthesis of 3-(adenine-9-yl)propyl acrylamide (AAm) *11*

Synthesis of 3-benzoylthymine *12*

Synthesis of 3-(3-benzoylthymin-1-yl)propyl acrylamide *13*

Synthesis of 3-(thymin-1-yl)propyl acrylamide (TAm) *13*

Synthesis of 3-(3-methylthymin-1-yl)-propylacrylamide (T^{Me}Am) *14*

Synthesis of 3-(N6,N6-dimethyladenine-9-yl)propyl acrylamide (A^{Me}Am) *15*

Polymer Syntheses 17

Synthesis of Poly(4-acryloylmorpholine) (PNAM$_{39}$) Macro-CTA via RAFT Polymerization *17*

S1.1.i Syntheses of Diblock Copolymers *18*

Assembly and Characterisation of Seed Nanoparticles NT 24

Self-Assembly of PT in Water *24*

Batch Addition of PA to NT *24*

Stepwise Addition of PA to NT *24*

SUPPLEMENTARY DISCUSSION 25

Analysis of Seed Nanoparticles NT 25

Analysis of the Morphological Transformation Products 25

DLS Analysis of the Transformation Products *25*

TEM Analysis of the Transformation Products *26*

CryoTEM Analysis of the Transformation Products *27*

LS and SAXS Analyses of the Transformation Products *28*

Stepwise Morphological Transformation of NT 31

Morphological Transformation at Low PA Concentrations *31*

Stepwise Transformation of Dumbbells *32*

Stepwise Growth of Long Wormlike Nanoparticles *32*

SAXS Analysis of Wormlike Nanoparticles *35*

Worm Disassembly at High A:T Ratios *38*

Morphological Transformation Control Experiments 40

Analysis of Mixtures of NT with PA^{Me}, PT1 and PS *40*

Analysis of the Aggregation Behaviour of PA^{Me}, PT1, PS and PA *42*

Blocking of H-Bonding in the Nanoparticle *44*

Self-Assembly by Solvent Switch from a Common Solvent *46*

MicroDSC Measurements *47*

Experiments Confirming Single Particle Transformation Process 49

SLS Analyses to Determine Nanoparticle Molecular Weights *49*

AFM Analyses of Nanoparticles *52*

The Effect of Altering Polymer Block Lengths on MORPH 53

Synthesis of PA with Different Block Lengths *53*

Effect of Increasing the Length of the Corona Block *53*

Effect of Varying the Length of the Adenine-Containing Block *55*

Fluorescent Tagging Using Morphological Transformation 57

Syntheses of Fluorescently-Labelled PA *57*

Stepwise Growth of Fluorescent Wormlike Nanoparticles *58*

Physical Model for MORPH 60

Relevant Timescales *60*

Comparison with Equilibrium Phenomena *61*

Swelling Dynamics *62*

MORPH Dynamics *63*

SUPPLEMENTARY REFERENCES 65

SUPPLEMENTARY METHODS

Materials

2,2′-Azo-bis(isobutyronitrile) (AIBN) was obtained from Molekula and recrystallized from methanol. 2,2′-Azobis[2-(2-imidazolin-2-yl)propane]dihydrochloride (VA-044, Wako) was used without further purification. 4-Acryloylmorpholine (NAM) was bought from Aldrich and was purified by vacuum distillation. 2-(((Butylthio)carbonothiolyl)thio)propanoic acid was synthesized as described previously and stored at 4 °C.[1] Wafers of p-silicon (100) were purchased from Sigma-Aldrich and cut into plates with a size of 10 mm × 10 mm for AFM imaging. Dialysis membranes (molecular weight cut-off = 3.5 kDa) were purchased from Spectra/Por. DMF, DMSO and other chemicals were obtained from Fisher Chemicals and used without further purification. Dry solvents were obtained by passing over a column of activated alumina using an Innovative Technologies solvent purification system.

Instrumentation & Analysis

NMR Spectroscopy

^{1}H NMR spectra were recorded on a Bruker DPX-400 or HD500 spectrometer with DMSO-d_6 as the solvent. The chemical shifts of protons were relative to solvent residues (DMSO 2.50 ppm, $CDCl_3$ 7.26 ppm).

Size Exclusion Chromatography (SEC)

SEC data were obtained in HPLC grade DMF containing 5 mM NH_4BF_4 at 50 °C, with a flow rate of 1.0 mL min^{-1}, on a set of two PLgel 5 μm Mixed-D columns, and a guard column. SEC data were analyzed with Cirrus SEC software calibrated using poly(methyl methacrylate) (PMMA) standards.

Refractive Index (RI) Measurements

Values for the refractive index increment (dn/dc) of polymers listed in Supplementary Table 1 were determined using a PSS DnDc1260 differential refractometer fitted with a 620 nm laser.

Supplementary Table 1. Refractive index increments (dn/dc) for the polymers and nanoparticle formulations used in this study.

**Sample**	**d*n*/d*c*** / mL g^{-1}
**PA**	0.162
**PT**	0.175
**NT** + **PT** (0.14 A:T)	0.174*
**NT** + **PT** (0.20 A:T)	0.172*

* Refractive index increments for mixed nanoparticle systems were calculated using a weighted sum of the dn/dc values of the individual copolymers.[1]

Dynamic Light Scattering (DLS) Analysis

Initial estimates for hydrodynamic diameters (D_H) and size distributions of particles were determined using a Malvern Zetasizer Nano S instrument fitted with a 4 mW He-Ne 633 nm laser module, which records measurements at a single detection angle, 173°. The proprietary software was used to calculate D_H according to the Stokes-Einstein equation for diffusion of particles through a liquid with low Reynolds number (see below).

In Supplementary Figures 7, 8, 20 and 22, 'PD' refers to (poly)dispersity index, a measure of the particle size distribution provided by the proprietary software, where

$$\mathrm{PD} = \log_{10}\left(\frac{\bar{M}_\mathrm{W}}{\bar{M}_\mathrm{N}}\right) \qquad \text{(Supplementary Equation 1)}$$

Note that in some cases DLS was used to qualitatively assess the solutions of anisotropic particles, but not to quantify parameters such as D_H – for these cases multi-angle light scattering was used (see below).

Static and Dynamic Light Scattering (LS) Analysis

LS experiments were conducted using an ALV-CGS3 goniometer-based system operating a λ = 633 nm wavelength laser, with the sample maintained at 25 °C. Samples were contained in 5 mm borosilicate glass tubes. Aliquots of the particles in solution at concentration c = 0.7 g L^{-1} were passed through 1.2 μm cellulose syringe filters and a dilution series prepared over the concentration range $0.2 \leq c \leq 0.7$ g L^{-1}. Intensity of light scattering, *I(t)*, was recorded over the angular range $30° \leq \theta \leq 130°$ at 5° intervals for each of the standard (toluene), the solvent (H_2O) and the solution. At each datum (*q, c*), where the magnitude of the scattering vector is

$$q = \left(\frac{4\pi}{\lambda}\right) \cdot n_\mathrm{D} \sin\left(\frac{\theta}{2}\right) \qquad \text{(Supplementary Equation 2)}$$

and n_{D} is the refractive index of the solvent, the proprietary software records the following measurements:

1. $\frac{R(q,c)}{Kc}$ (Supplementary Equation 3)

   which is the Rayleigh ratio,

   $$\left(\frac{I_{\mathrm{solution}}-I_{\mathrm{solvent}}}{I_{\mathrm{standard}}}\right) \cdot I_{\mathrm{std(abs)}} \quad \text{(Supplementary Equation 4)}$$

   normalized with respect to sample concentration, *c*, and an instrument constant,

   $$K = \left(\frac{4\pi^2}{\lambda^4 N_{\mathrm{A}}}\right) \cdot \left(n_{\mathrm{D}} \cdot \frac{dn}{dc}\right)^2 \quad \text{(Supplementary Equation 5)}$$

   where N_{A} is the Avogadro number and $\frac{dn}{dc}$ is the refractive index increment (see below).

2. The normalized scattering intensity autocorrelation function,

   $$g_2(q,\tau) = \frac{\langle I(q,t)I(q,t+\tau)\rangle}{\langle I(q,\tau)^2\rangle} \quad \text{(Supplementary Equation 6)}$$

   and from this the amplitude correlation function,

   $$g_1(q,\tau) \quad \text{(Supplementary Equation 7)}$$

   according to the Siegert relation,

   $$g_2(q,\tau) = 1 + g_1(q,\tau)^2 \quad \text{(Supplementary Equation 8)}$$

   both of these calculated by the ALV LSE-5004 correlator module.

Zimm plots were constructed using the Berry transformation, as recommended by Andersson,[2] using a first order polynomial fit for both variables to perform the double extrapolation

$$\frac{R}{Kc}(q \to 0, c \to 0) \quad \text{(Supplementary Equation 9)}$$

and thereby estimate the intensity weighted radius of gyration, $\langle R_{\mathrm{G}}\rangle_{\mathrm{Z}}$, mass average molar mass, $\overline{M}_{\mathrm{W}}$, and a virial coefficient to represent pairwise interactions amongst particles, A_2.

The REPES algorithm was used to determine relaxation rates, $\tau^{-1}(\theta, c)$, that were consistent with a diffusion process from the amplitude correlation function. The intensity weighted mean translational diffusion coefficient, *D*, was then estimated according to the relation

$$\tau^{-1} = Dq^2 \qquad \text{(Supplementary Equation 10)}$$

and thereby the hydrodynamic radius, $\langle R_\mathrm{H} \rangle_\mathrm{Z}$, calculated according to the Stokes-Einstein equation,

$$\langle R_\mathrm{H} \rangle_\mathrm{Z} = \frac{k_\mathrm{B}T}{6\pi\eta D} \qquad \text{(Supplementary Equation 11)}$$

where k_B is the Boltzmann constant, *T* is solution temperature and $\eta(T)$ is the kinematic viscosity of the solvent. Statistical analysis and parameter fitting were conducted using R statistical software and the library 'FME'.[3]

Small-Angle X-Ray Scattering (SAXS) Analysis

Small-angle X-ray scattering (SAXS) measurements were made using a Xenocs Xeuss 2.0 equipped with a micro-focus Cu K_α source collimated with Scatterless slits. The scattering was measured using a Pilatus 300k detector with a pixel size of 0.172 mm × 0.172 mm. The distance between the detector and the sample was calibrated using silver behenate ($AgC_{22}H_{43}O_2$), giving a value of 2.481(5) m. Samples were mounted in 1 mm borosilicate glass capillaries.

Transmission Electron Microscopy (TEM)

TEM observations were performed on a JEOL 2100 electron microscope at an acceleration voltage of 200 kV. All TEM samples were prepared on graphene-oxide (GO)-coated lacey carbon grids (400 Mesh, Cu, Agar Scientific), to enable high contrast TEM images without any staining.[4] Generally, a drop of sample (10 μL) was pipetted onto a grid and left for several minutes, then blotted away. TEM images were analyzed using the ImageJ software, and over 100 particles were counted for each sample to obtain number-average diameter D_n (for spheres), length L_n and width W_n (for worms). Volumes of worms were calculated according to volume,

$$V = \pi W_n^2 L_n/4 \quad \text{(Supplementary Equation 12)}$$

Atomic Force Microscopy (AFM)

AFM imaging and analysis were performed on an Asylum Research MFP3D-SA atomic force microscope in tapping mode. Samples for AFM analysis were prepared by drop casting 5 μL of solution (0.1 mg mL^{-1}) onto a silicon wafer that had been freshly cleaned with water and ethanol, then activated using plasma treatment to generate a hydrophilic surface.

Confocal Microscopy

Confocal microscopy images were taken using a Zeiss LSM 880 confocal fluorescent microscope. The solution of the assembly being studied (5 μL of a 0.1 mg mL^{-1} in H_2O) was dropped onto a plasma-cleaned microscope slide and left to dry overnight. Assemblies tagged with BODIPY-FL dye (green) were excited using a 488 nm laser, while assemblies tagged with BODIPY-TR dye (red) were excited using a 633 nm laser. Both channels were used at the same time to detect the presence of green or red assemblies and overlays were produced.

Micro-Differential Scanning Calorimetry (microDSC)

MicroDSC measurements were performed on a Nano Differential Scanning Calorimeter 602000 (TA instruments) at 3 atm pressure and with a heating and cooling rate of 0.2 °C min^{-1}. A volume of 400 μL of solution was introduced into the sample and reference capillary cells. 18.2 MΩ cm water was used to baseline all measurements. The instrument was controlled by the DSCrun software. The data was analysed with the NanoAnalyze software.

Monomer Syntheses

Synthesis of N-(3-bromopropyl)acrylamide

N-(3-Bromopropyl) acrylamide was synthesized using procedures similar to the previous literature.[1] To a solution of 3-bromopropylamine (10.1 g, 45 mmol), triethylamine (TEA) (14 mL, 100 mmol) and 4-(dimethylamino)pyridine (DMAP) (288 mg, 2.3 mmol) in CH_2Cl_2 (150 mL), acryloyl chloride (4.2 mL, 50 mmol) was added dropwise in an ice bath and then left at room temperature for another 4.5 h. The reaction solution was washed with saturated $NaHCO_3$ aqueous solution (100 mL) and water (2 × 100 mL). The organic layer was collected and dried with anhydrous $MgSO_4$ and filtered. Then 2,6-bis(1,1-dimethylethyl)-4-methylphenol (6.3 mg, 1.5 mmol) was added to the filtrate followed by concentration under vacuum to give a brown oil. The brown oil (6.3 g, 73%) was used for the following reaction immediately without further purification. ^1H NMR (400 MHz, $CDCl_3$) δ = 6.27 (d, J = 16.8 Hz, 1H, C*H*=CH-CO), 6.11 (d, J = 10.0 Hz, 1H, C*H*=CH-CO), 6.06 (s, 1H, N*H*CO), 5.63 (dd, J = 16.8 Hz, 10.0 Hz, 1H, CH_2=C*H*-CO), 3.42-3.50 (m, 4H, CH_2-CH_2-CH_2-Br), 2.08-2.15 (m, 2H, CH_2-CH_2-CH_2-Br) ppm; ^{13}C NMR (100 MHz, $CDCl_3$) δ = 166.0, 130.8, 126.7, 38.2, 32.2, 31.0 ppm.

Synthesis of 3-(adenine-9-yl)propyl acrylamide (AAm)

The **AAm** monomer was synthesised according to our previous work.[1] To a suspension of adenine (3.0 g, 24.2 mmol) in dry DMF (100 mL), 60% NaH dispersed in mineral oil (1.0 g, 25.4 mmol NaH) was slowly added in small portions under a nitrogen atmosphere. The mixture was stirred for 1 h until no gas was produced. The viscous mixture was immersed into an ice bath and *N*-(3-bromopropyl) acrylamide freshly synthesized (5.4 g, 28.2 mmol) was added dropwise. The ice bath was left in place and the yellow viscous mixture was stirred overnight. The resulting suspension was concentrated under high vacuum at 50 °C to give a highly viscous oil, to which CH_2Cl_2 was added and the contents mixed by gentle swirling. The CH_2Cl_2 was then poured off and the process repeated several times, followed by concentration under vacuum. The crude residue was further purified by column chromatography

using a mixture of CH_2Cl_2 and CH_3OH as eluent and a gradient from 1:0 to 9:1* to give a white solid, **AAm** (3.18 g, 52%). ^1H NMR (400 MHz, DMSO-d_6) δ = 8.19 (t, J = 5.2 Hz, CONH), 8.15 (s, 1H, purine H-2), 8.14 (s, 1H, purine H-8), 7.20 (s, 2H, NH_2), 6.21 (dd, J = 16.8 Hz, 10.0 Hz, 1H, CH_2=CH-CO), 6.08 (dd, J = 16.8 Hz, 2.0 Hz, 1H, CH=CH-CO), 5.59 (dd, J = 10.0 Hz, 2.0 Hz, 1H, CH=CH-CO), 4.15 (t, 2H, J =6.8 Hz, CH_2-purine) 3.13 (m, 2H, OC-NH-CH_2), 1.97 (m, 2H, OC-NH-CH_2-CH_2-CH_2-purine) ppm; 13C NMR (100 MHz, DMSO-d_6) δ = 165.6, 153.3, 150.4, 148.3, 141.8, 132.6, 126.1, 119.7, 41.8, 36.8, 30.4 ppm; HR-MS (m/z) found 269.1119, calc. 269.1127 $[M+Na]^+$.

Synthesis of 3-benzoylthymine

Following the procedures in a previous report,[1] benzoyl chloride (11.24 mL, 96.8 mmol) and thymine (3.0 g, 24.2 mmol) were suspended in a mixture of acetonitrile (30 mL) and pyridine (12 mL) under nitrogen. The reaction was stirred under a nitrogen atmosphere supplied by a balloon at room temperature overnight. The reaction solution was then concentrated under vacuum. The viscous liquid was partitioned between CH_2Cl_2 and water. The aqueous layer was extracted three times with CH_2Cl_2 and the combined organic layers were dried over anhydrous K_2CO_3. The solvent was removed under vacuum. The residue was dissolved in dioxane (30 mL) and K_2CO_3 (4.1 g) in 30 mL of water was added and the reaction mixture was stirred until TLC showed complete conversion to the mono-protected thymine (around 2 h). The crude product was concentrated and colourless crystals crystallised from the solution upon standing at room temperature and further cooling to 4 °C. The crystals were isolated by filtration and washed on the filter with water to remove salt impurities, then dried to yield the final product (4.5 g, 80%). ^1H NMR (400 MHz, DMSO-d_6) δ = 11.4 (br, 1H, pyrimidine-H1), 7.94 (d, J = 10.0 Hz, 2H, benzene-H1,H5), 7.77 (t, J = 10.0 Hz, 1H, benzene-H3), 7.59 (d, J = 10.0 Hz, 2H, benzene-H2,H4), 7.53 (s, 1H, pyrimidine-H6), 1.82 (d, 3H, J = 6.8 Hz, CH_3-

* Approximately 500 mL of eluent was used for 1:0, 99:1, 95:5, 93:7 and 91:9 $CHCl_3$:MeOH during the gradient column.

pyrimidine) ppm; ^{13}C NMR (100 MHz, DMSO-d_6) δ = 170.7, 164.1, 150.5, 139.3, 135.8, 131.9, 130.7, 129.9, 108.4, 12.2 ppm.

Synthesis of 3-(3-benzoylthymin-1-yl)propyl acrylamide

To a solution of 3-benzoylthymine (2.3 g, 10.0 mmol) in dry DMF (50 mL), 60% NaH (0.42 g, 10.5 mmol NaH) was slowly added. The mixture was stirred for 1 h until no gas was produced. The viscous mixture was immersed in an ice bath and *N*-(3-bromopropyl) acrylamide freshly synthesized (2.3 g, 12.0 mmol) was added dropwise. The ice bath was left in place and the yellow, viscous mixture was stirred overnight. The resulting solution was concentrated under high vacuum at 50 °C. The residue was partitioned between EtOAc and water. The aqueous layer was extracted three times with EtOAc and the combined organic layers were dried over anhydrous $MgSO_4$. The solvent was removed under vacuum. The mixture was further purified by column chromatography using EtOAc as eluent to give a viscous liquid (2.0 g, 58%). ^1H NMR (400 MHz, DMSO-d_6) δ = 8.17 (t, J = 5.2 Hz, CON*H*), 7.96 (d, J = 6.0 Hz, 2H, benzene-*H*1,*H*5), 7.79 (s, 1H, pyrimidine-*H*6), 7.77 (t, J = 6.0 Hz, 1H, benzene-*H*3), 7.59 (d, J = 6.0 Hz, 2H, benzene-*H*2, *H*4), 6.20 (dd, J = 17.0 Hz, 10.0 Hz, 1H, CH_2=C*H*-CO), 6.10 (dd, J = 17.0 Hz, 2.0 Hz, 1H, C*H*=CH-CO), 5.58 (dd, J = 10.0 Hz, 2.0 Hz, 1H, C*H*=CH-CO), 3.73 (t, 2H, J = 7.0 Hz, CH_2-pyrimidine), 3.20 (m, 2H, OC-NH-CH_2), 1.84 (d, 3H, J = 6.8 Hz, CH_3-pyrimidine), 1.82 (m, 2H, OC-NH-CH_2-CH_2-CH_2-pyrimidine) ppm; ^{13}C NMR (100 MHz, DMSO-d_6) δ = 170.3, 165.2, 163.4, 149.9, 143.0, 135.9, 132.1, 131.7, 130.8, 130.0, 125.6, 109.0, 46.6, 36.3, 28.9, 12.3 ppm.

Synthesis of 3-(thymin-1-yl)propyl acrylamide (TAm)

The **TAm** was synthesised as reported previously.[1] (3-Benzoylthymin-1-yl)propyl acrylamide (2.0 g, 5.9 mmol) was dissolved in a mixture of TFA/CH_2Cl_2 (3:1) (20 mL). The reaction solution was stirred at room temperature overnight. After completion of the reaction, solvent was removed under vacuum. The residue was purified by column chromatography with a gradient of $CHCl_3$/CH_3OH from 1:0 to

93:7[†] to give a viscous liquid. Ethanol (20 mL) was then added and the solution cooled to −20 °C to precipitate a white solid[‡], **TAm** (1.0 g, 70%). ^{1}H NMR (500 MHz, DMSO-d_6) δ = 11.23 (s, 1H, pyrimidine-*H*3), 8.12 (t, J = 5.2 Hz, CON*H*), 7.51 (s, 1H, pyrimidine-*H*6), 6.18 (dd, J = 16.8 Hz, 10.0 Hz, 1H, CH_2=C*H*-CO), 6.07 (dd, J = 16.8 Hz, 2.0 Hz, 1H, C*H*=CH-CO), 5.58 (dd, J = 10.0 Hz, 2.0 Hz, 1H, C*H*=CH-CO), 3.63 (t, 2H, J = 6.8 Hz, CH_2-pyrimidine), 3.14 (m, 2H, OC-NH-CH_2), 1.74 (d, 3H, J = 1.0 Hz, CH_3-pyrimidine), 1.74 (m, 2H, OC-NH-CH_2-CH_2-CH_2-pyrimidine) ppm; ^{13}C NMR (125 MHz, DMSO-d_6) δ = 165.1, 164.8, 151.3, 142.0, 132.2, 125.6, 108.9, 45.9, 36.4, 29.1, 12,4 ppm; HR-MS (m/z) found 260.1004, calc. 260.1011 $[M+Na]^+$.

Synthesis of 3-(3-methylthymin-1-yl)-propylacrylamide (T^{Me}Am)

A mixture of 3-(thymin-1-yl)- propylacrylamide (**TAm**) (71 mg, 0.3 mmol), dry K_2CO_3 (66 mg, 0.48 mmol), and iodomethane (75 µL) in anhydrous DMF (0.4 mL) was stirred at room temperature for 24 h and then diluted with ethyl acetate (20 mL), washed with water (2 × 20 mL), and dried with anhydrous Na_2SO_4. The solvent was removed under vacuum. The mixture was further purified by column chromatography with a mixture of CH_2Cl_2/CH_3OH (95:5) to give a white solid, **T^{Me}Am** (73 mg, 0.29 mmol, 97%). ^{1}H NMR (500 MHz, DMSO-d_6) δ = 8.12 (t, J = 5.0 Hz, CON*H*), 7.59 (s, 1H, pyrimidine-*H*6), 6.18 (dd, J = 17.5, 10.5 Hz, 1H, CH_2-C*H*−CO), 6.07 (dd, J = 17.5, 2.0 Hz, 1H, C*H*- CH−CO), 5.58 (dd, J = 10.5, 2.0 Hz, 1H, C*H*-CH−CO), 3.70 (t, 2H, J = 7.5 Hz, CH_2-pyrimidine), 3.17 (s, 3H, OC−NCH_3), 3.14 (m, 2H, OC−HN−CH_2), 1.80 (s, 3H, CH_3-pyrimidine), 1.76 (m, 2H, OC− NH−CH_2−CH_2−CH_2-pyrimidine) ppm. ^{13}C NMR (125 MHz, DMSO-d_6) δ = 165.1, 163.8, 151.5, 140.4, 132.2, 125.5, 107.9, 47.1, 36.3, 29.0, 28.0, 13.1 ppm; HR-MS (m/z) found 274.1165, calcd 274.1162 $[M + Na]^+$.

[†] Approximately 500 mL of eluent was used for 1:0, 99:1, 95:5 $CHCl_3$:MeOH during the gradient column.
[‡] In some cases further concentration and/or addition of Et_2O was required to trigger the crystallisation.

Synthesis of 3-(*N*6,*N*6-dimethyladenine-9-yl)propyl acrylamide ($A^{Me}Am$)

To a suspension of *N*6,*N*6-dimethyladenine (0.16 g, 1.0 mmol) in dry DMF (5 mL), NaH (0.025 g, 1.05 mmol) was slowly added (Supplementary Figure 1). The mixture was stirred for 1 h until no gas was produced. The viscous mixture was immersed into an ice bath and 3-bromopropyl acrylamide freshly synthesized (0.23 g, 1.2 mmol) was added dropwise. The yellow viscous mixture was stirred overnight and the resulting suspension was concentrated under vacuum. The obtained mixture was purified by column chromatography using a mixture of CH_2Cl_2 and CH_3OH as eluent and a gradient from 1:0 to 95:5 to give a white solid, **$A^{Me}Am$** (0.22 g, 80%). Assigned ^1H, ^{13}C NMR spectra are shown in Supplementary Figure 2.

^1H NMR (500 MHz, DMSO-d_6) δ = 8.21 (s, 1H, purine *H*-2), 8.17 (s, 1H, purine *H*-8), 8.19 (t, *J* = 4.5 Hz, 1H, CON*H*), 6.20 (dd, *J* = 17.0 Hz, 10.0 Hz, 1H, CH_2=C*H*-CO), 6.07 (dd, *J* = 17.0 Hz, 2.0 Hz, 1H, CH_2=CH-CO), 5.59 (d, *J* = 10.0 Hz, 2.0 Hz, 1H, CH_2=CH-CO), 4.17 (t, 2H, *J* = 6.5 Hz, CH_2-purine) 3.45 (s, 6H, purine N-(CH_3)$_2$), 3.12 (q, 2H, *J* = 6.5 Hz, OC-NH-CH_2), 1.96 (m, 2H, *J* = 6.5 Hz, OC-NH-CH_2-CH_2-CH_2-purine) ppm. ^{13}C NMR (125 MHz, DMSO-d_6) δ = 165.1, 154.7, 152.2, 150.7, 140.2, 132.2, 125.6, 119.7, 41.4, 40.2, 36.3, 29.9 ppm. HR-MS (m/z) found 275.1616, calc. 275.1615 $[M+H]^+$.

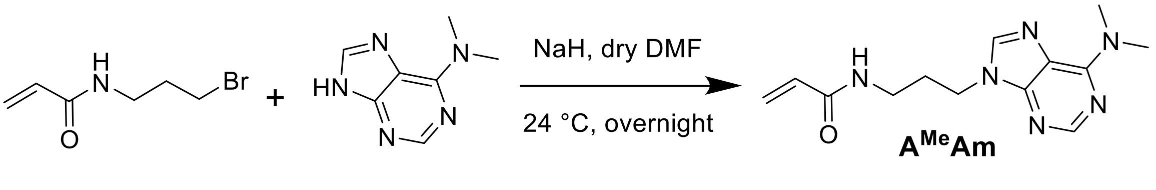

Supplementary Figure 1. The synthesis of 3-(*N*6,*N*6-dimethyladenine-9-yl)propyl acrylamide (**$A^{Me}Am$**).

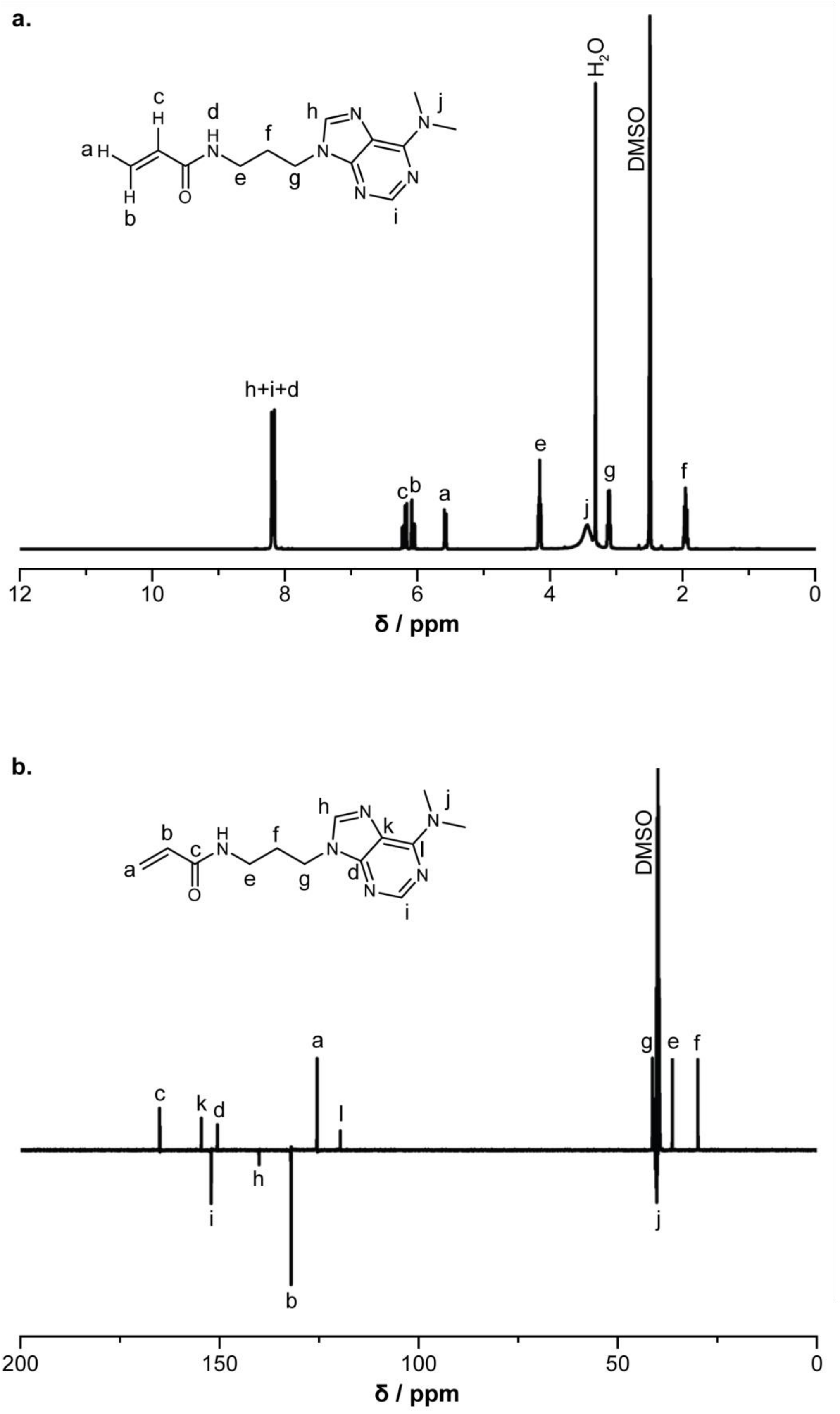

Supplementary Figure 2. Assigned ^{1}H, ^{13}C NMR spectra of $\mathbf{A^{Me}Am}$.

Polymer Syntheses

The synthetic strategies for the macroCTA, **PA** and **PT** are shown in Supplementary Figure 3 – the other polymers used were synthesised using similar procedures. Characterization data for all polymers are shown in Supplementary Table 2, with ^{1}H NMR spectra presented in Supplementary Figure 4Supplementary **Figure 5** and Supplementary **Figure 8**a, and size exclusion chromatograms in Supplementary Figure 6Supplementary **Figure 7** and Supplementary **Figure 8**b.

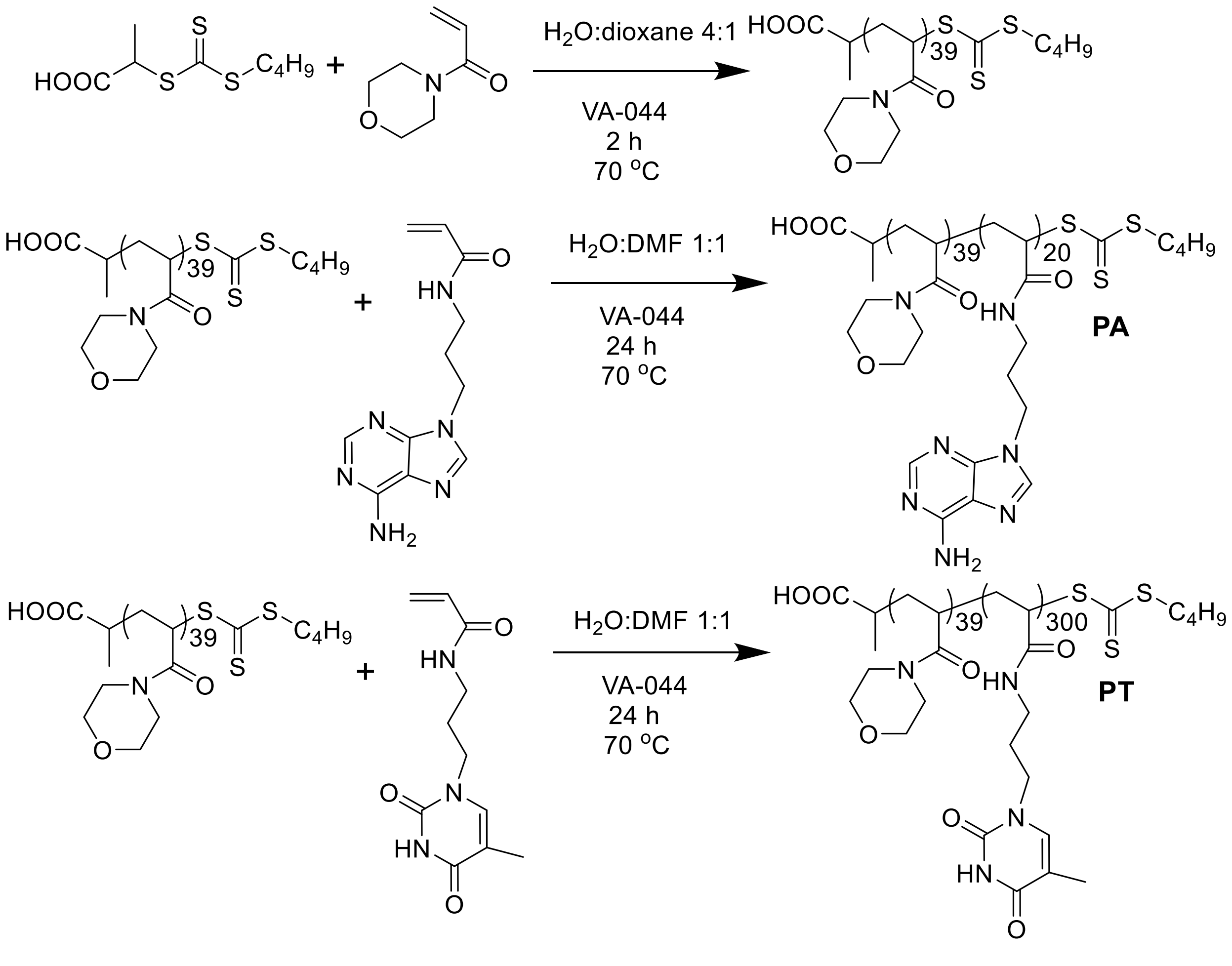

Supplementary Figure 3. Synthetic routes for PNAM$_{39}$, **PA** (PNAM$_{39}$-*b*-PAAm$_{20}$) and **PT** (PNAM$_{39}$-*b*-PTAm$_{300}$).

Synthesis of Poly(4-acryloylmorpholine) ($PNAM_{39}$) Macro-CTA *via* RAFT Polymerization

The procedures were similar to our previous work.[1] The typical procedure was as follows. For $PNAM_{39}$, a 10 mL ampoule was charged with NAM (500 µL, 4.0 mmol), 2-(((butylthio)carbonothiolyl)thio)-propanoic acid (23.8 mg, 0.1 mmol), VA-044 (1.3 mg, 0.004 mmol) and a mixture of 1,4-dioxane and water (2.0 mL, v:v 1:4). The mixture was thoroughly degassed *via* 4 freeze-pump-thaw cycles, filled with nitrogen and then immersed in an oil bath at 70 °C for 2 h. The polymerization solution was precipitated three times from cold CH_3OH. The light yellow polymer was dried in a vacuum oven overnight at room temperature and analyzed by ^1H NMR spectroscopy and DMF SEC (Supplementary Figure 4 and Supplementary **Figure 6**). The degree of polymerization (DP) of this PNAM macro-CTA was calculated to be 39 using ^1H NMR spectroscopy by comparing the integrated signals corresponding to the backbone signals (δ = 1.62 ppm) with those of the methyl group from the CTA (δ = 0.87 ppm).

S1.1.i Syntheses of Diblock Copolymers

The typical procedure was as follows. For $PNAM_{39}$-*b*-$PTAm_{300}$, $PNAM_{39}$ (14 mg, 0.0025 mmol), **TAm** (178 mg, 0.75 mmol), and VA-044 (0.08 mg, 0.00025 mmol) were dissolved in a mixture of DMF and water (0.5 mL, v:v 1:1). The mixture was thoroughly degassed *via* 4 freeze-pump-thaw cycles, filled with nitrogen and then immersed in an oil bath at 70 °C overnight. An aliquot of the crude product was taken and analyzed by ^1H NMR spectroscopy to calculate the conversion. The degree of polymerization (DP) of obtained diblock copolymers was calculated using the conversion from ^1H NMR spectroscopy. The residual solution was then precipitated three times from cold CH_3OH. The light yellow polymer was dried in a vacuum oven overnight at room temperature and analysed by ^1H NMR spectroscopy and DMF SEC. See Supplementary Table 2 for NMR and SEC characterization of polymers used.

Supplementary Table 2. Characterization data for the macroCTA and nucleobase-containing diblock copolymers.

Polymer	$M_{n,NMR}$* / kDa	$M_{n,SEC}$† / kDa	$Đ_M$†
$PNAM_{39}$	5.7	5.9	1.07
$PNAM_{39}$-*b*-$PTAm_{300}$ **PT**	76.9	58.5	1.33
$PNAM_{39}$-*b*-$PAAm_{20}$ **PA**	10.7	12.6	1.07
$PNAM_{39}$-*b*-$PMAAm_{20}$ **PA^{Me}**	11.2	9.7	1.12
$PNAM_{39}$-*b*-$PTAm_{20}$ **PT1**	10.5	13.1	1.07
$PNAM_{39}$-*b*-PSt_{20} **PS**	7.8	8.0	1.09
$PNAM_{39}$-*b*-$PMTAm_{300}$ **PT^{Me}**	81.1	53.7	1.35

* Determined by ^{1}H NMR spectroscopy (400 MHz) in deuterated DMSO. † Determined by DMF SEC, with poly(methyl methacrylate) (PMMA) standards.

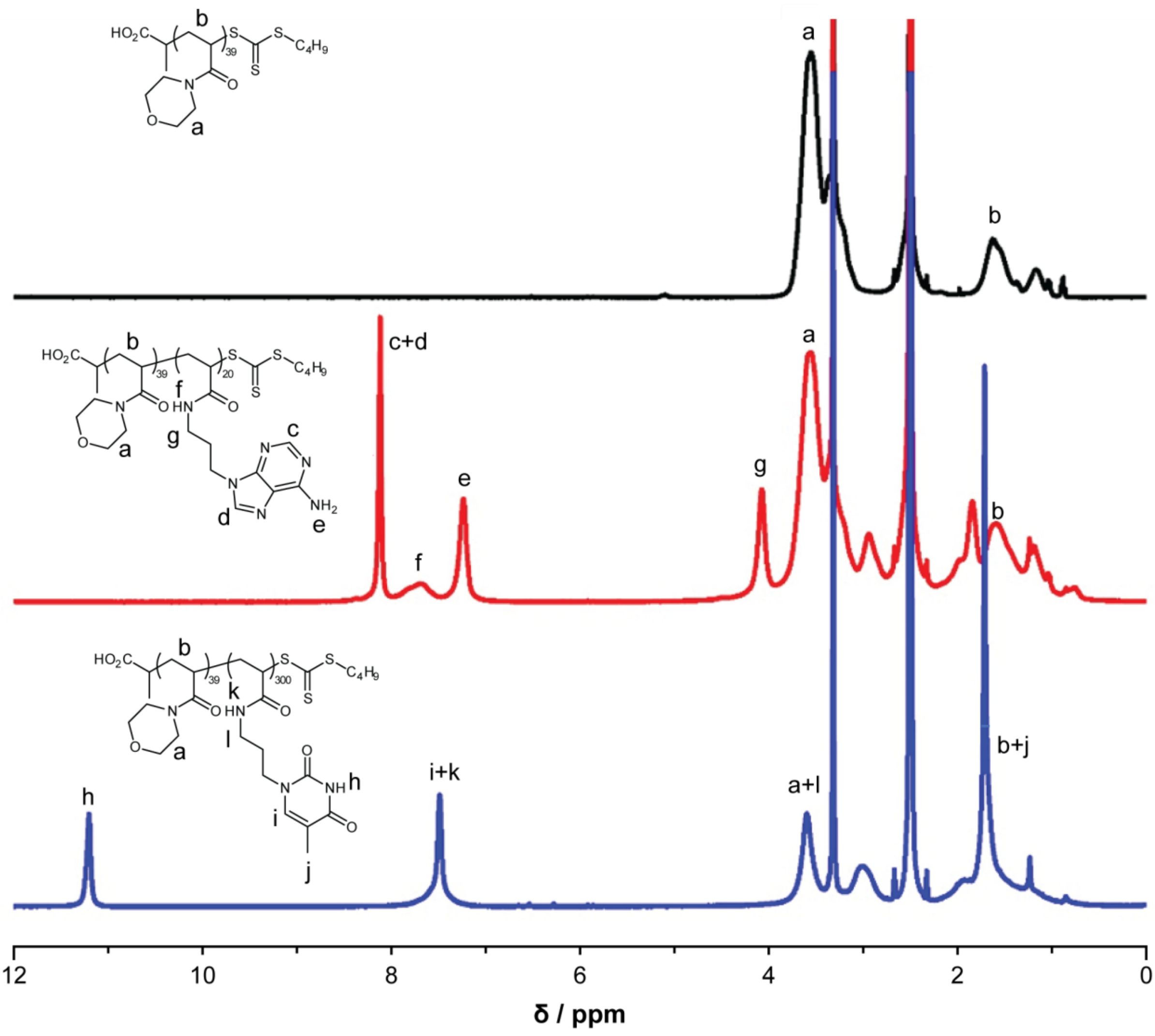

Supplementary Figure 4. ^{1}H NMR spectra of $PNAM_{39}$, **PA** ($PNAM_{39}$-*b*-$PAAm_{20}$) and **PT** ($PNAM_{39}$-*b*-$PTAm_{300}$) (400 MHz, d_6-DMSO).

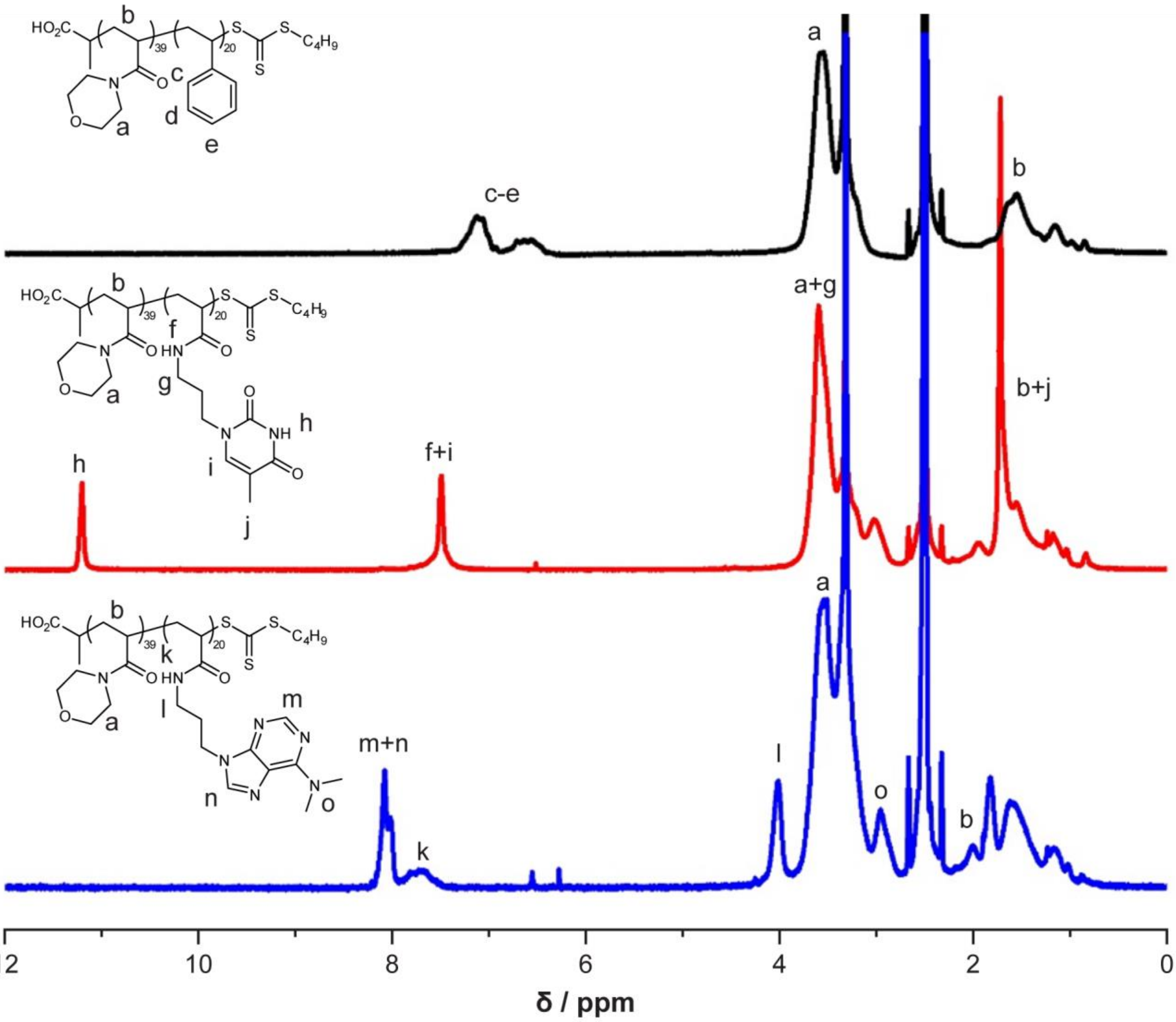

Supplementary Figure 5. ¹H NMR spectra of **PS** (PNAM$_{39}$-*b*-PSt$_{20}$), **PT1** (PNAM$_{39}$-*b*-PTAm$_{20}$) and **PA^{Me}** (PNAM$_{39}$-*b*-PMAAm$_{20}$) (400 MHz, d_6-DMSO).

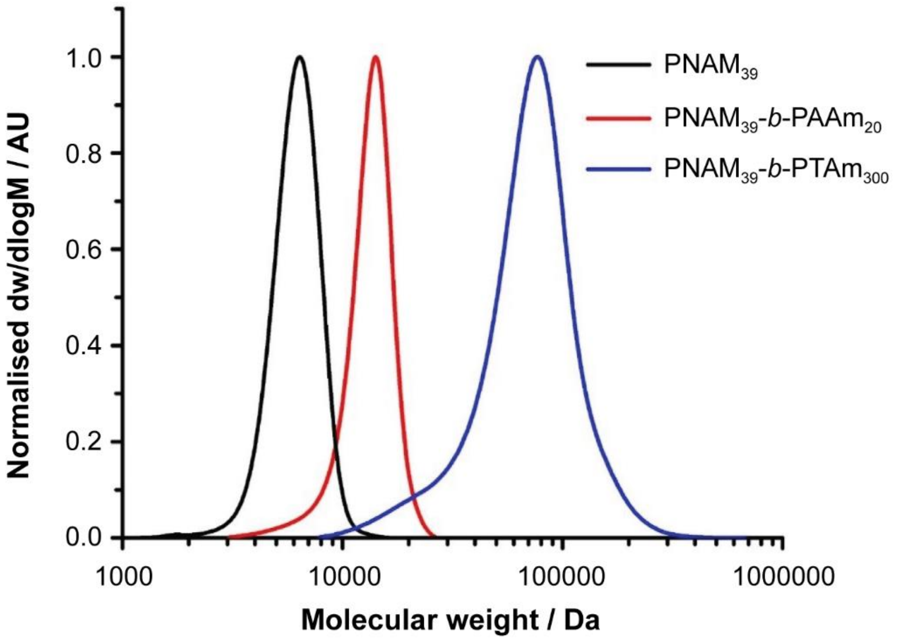

Supplementary Figure 6. Size exclusion chromatograms of $PNAM_{39}$, **PA** ($PNAM_{39}$-*b*-$PAAm_{20}$) and **PT** ($PNAM_{39}$-*b*-$PTAm_{300}$) from DMF SEC using poly(methyl methacrylate) (PMMA) standards.

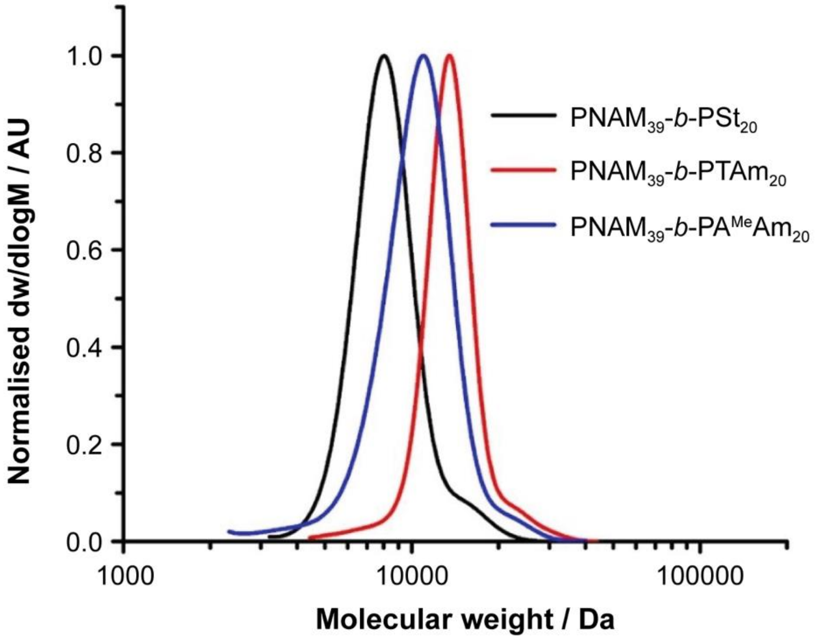

Supplementary Figure 7. Size exclusion chromatograms for **PS** (PNAM39-*b*-PSt_{20}), **PT1** ($PNAM_{39}$-*b*-$PTAm_{20}$) and **PA^{Me}** ($PNAM_{39}$-*b*-$PMAAm_{20}$) from DMF SEC using poly(methyl methacrylate) (PMMA) standards.

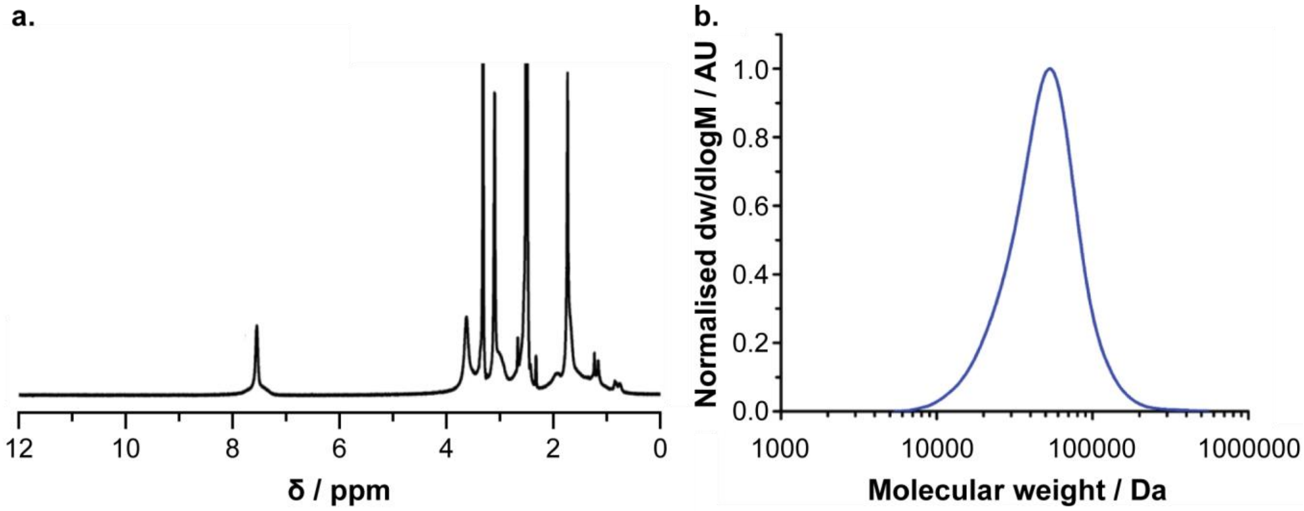

Supplementary Figure 8. (a) ^{1}H NMR spectrum of **PT$^{\mathbf{Me}}$** (PNAM$_{39}$-*b*-PMTAm$_{300}$) (400 MHz, d_6-DMSO); (b) Size exclusion chromatogram of **PT$^{\mathbf{Me}}$** (PNAM$_{39}$-*b*-PMTAm$_{300}$) from DMF SEC using poly(methyl methacrylate) (PMMA) standards.

Assembly and Characterisation of Seed Nanoparticles NT

Self-Assembly of PT in Water

The seed nanoparticles **NT** were assembled as follows. The copolymer was dissolved in DMF (at 8 mg mL^{-1}) and stirred for 2 h at 70 °C. Then an excess of 18.2 MΩ·cm water was added *via* a syringe pump at a rate of 1 mL h^{-1}. The final volume ratio between water and organic solvent was about 8:1. The solution was then dialyzed against 18.2 MΩ·cm water, incorporating at least 6 water changes, to afford self-assemblies **NT** at a concentration of *ca.* 1 mg mL^{-1}.

Batch Addition of PA to NT

The typical procedure was as follows. The diblock copolymer $PNAM_{39}$-*b*-$PAAm_{20}$ **PA** was dispersed in H_2O at 5 mg mL^{-1}. This was then added to separate solutions of the nanoparticle **NT** (0.5 mg mL^{-1}) with stirring at A:T molar ratios of 0.07, 0.20, 0.33, 0.67, 1.0, 1.33. The molar ratios were calculated according to the M_n determined from ^{1}H NMR spectroscopic analyses and the polymers' mass concentrations. The mixtures were then sealed and allowed to stir at room temperature for 2 h. The solutions were then characterized by DLS and TEM.

Stepwise Addition of PA to NT

The procedure was as follows: a solution of **PA** (0.33 molar ratio of A relative to T) was added to the nanoparticle **NT** solution (0.5 mg mL^{-1}) to give short "seed" worms. After 2 h stirring, further **PA** solution (0.07 molar ratio A relative to T) was added. This process was repeated until A:T ratios of 0.33, 0.40, 0.53 and 0.67 were achieved. Each stage was characterised by TEM and SAXS analyses.

SUPPLEMENTARY DISCUSSION

Analysis of Seed Nanoparticles NT

DLS analysis of **NT** is presented in Supplementary Figure 9. See the main paper for TEM images and Supplementary Figure 28 for further SLS characterisation, and Supplementary Figure 12 for cryoTEM images.

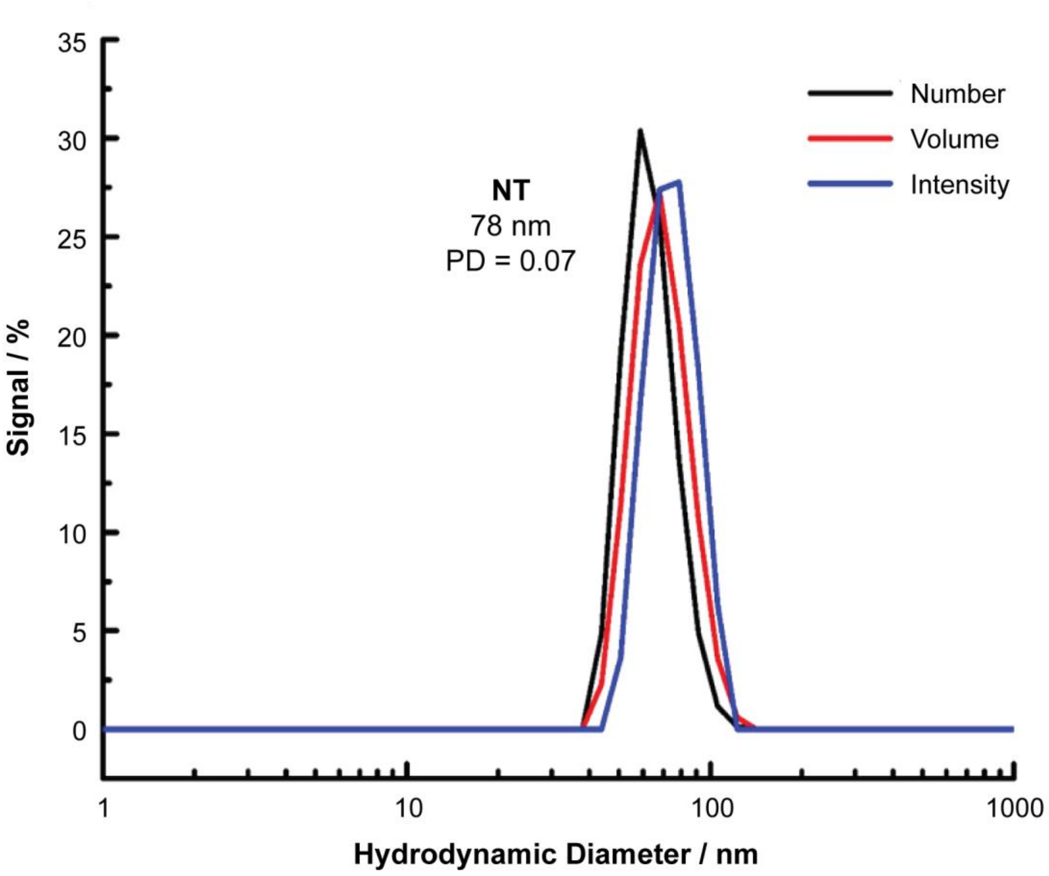

Supplementary Figure 9. DLS analysis of nanoparticles **NT** (0.5 mg mL^{-1}) in water.

Analysis of the Morphological Transformation Products

DLS Analysis of the Transformation Products

The very long worms generated by the morphological transformation process were not amenable to analysis by single angle DLS, since this assumes a spherical particle. However, the dumbbells formed

by addition of **PA** to **NT** at an A:T molar ratio of 0.20 were small and compact enough for effective single angle DLS analysis, which is presented in Figure S8.

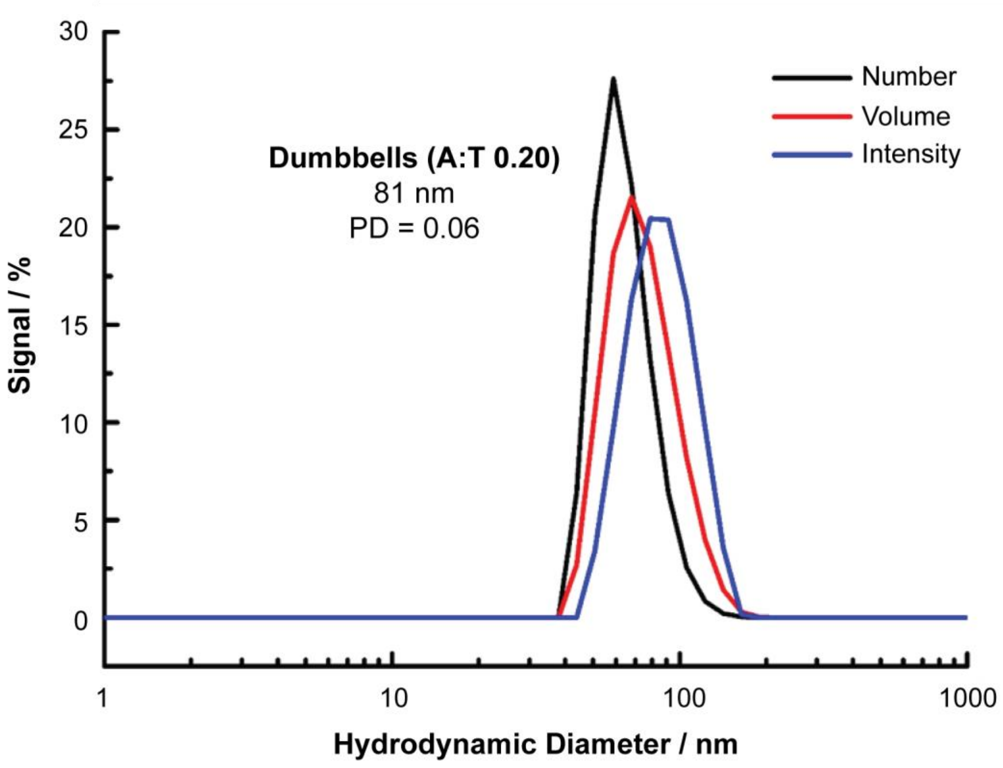

Supplementary Figure 10. DLS analysis of dumbbells (0.5 mg mL^{-1}) formed from **NT** after adding **PA** at an **A:T** molar ratio of 0.20. Note that due to particle anisotropy these single angle DLS measurements are presented for qualitative comparison purposes only.

TEM Analysis of the Transformation Products

Further TEM images of the transformation products formed by the addition of a single dose of **PA** at different A:T molar ratios are presented in Supplementary Figure 11.

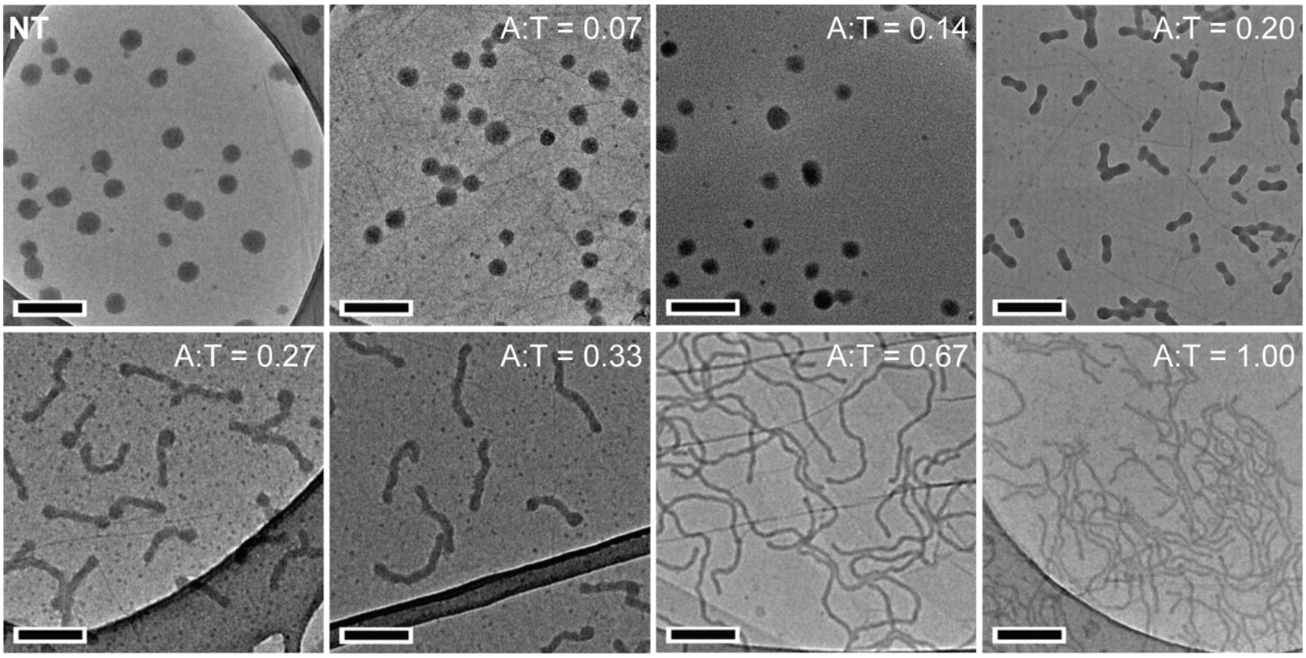

Supplementary Figure 11. Further TEM images of the transformation products formed by the addition of a single dose of **PA** to **NT** at the stated A:T molar ratios. Scale bars = 200 nm.

CryoTEM Analysis of the Transformation Products

To rule out the possibility that the morphological transformation products were artefacts observed in dry state TEM, we performed cryoTEM on the same samples. Images are presented in Supplementary Figure 12, which demonstrate that the structures were not drying artefacts.

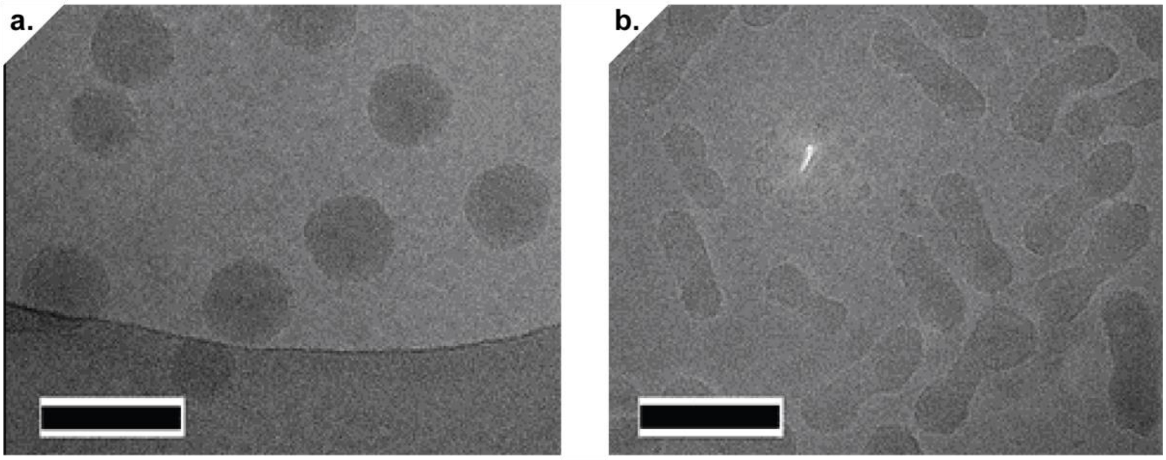

Supplementary Figure 12. Cyro-TEM images of (a) spherical nanoparticles **NT**; (b) dumbbell-like micelles formed by adding **PA** to **NT** at an A:T molar ratio of 0.20. Scale bars = 100 nm.

LS and SAXS Analyses of the Transformation Products

The morphologies of the transformation products were investigated using LS and SAXS. The aim of these analyses was to verify that the particles observed by TEM were representative of the bulk sample.

- LS: This was accomplished by comparing the values obtained for $\langle R_{\mathrm{G}}\rangle_{\mathrm{Z}}$ and $\langle R_{\mathrm{H}}\rangle_{\mathrm{Z}}$. The ratio $R_{\mathrm{G}}/R_{\mathrm{H}}$ may be interpreted as measure of a particle's compactness, e.g. the theoretical value for a homogeneous sphere is $R_{\mathrm{G}}/R_{\mathrm{H}} = 0.775$ and the value for a random coil $R_{\mathrm{G}}/R_{\mathrm{H}} \approx 1.5$.[5] As shown in Supplementary Table 3, the empirical values for this quantity, $\langle R_{\mathrm{G}}\rangle_{\mathrm{Z}}/\langle R_{\mathrm{H}}\rangle_{\mathrm{Z}}$, increase in correspondence with an increase in A:T molar ratio, in a way that is consistent with the respective particles exhibiting greater anisotropy.

1. SAXS: This was accomplished by fitting an appropriately parameterised model to the empirical form factor. The analytical expressions for (a) a homogeneous sphere and (b) a homogeneous ellipsoid are given by Pedersen[6], i.e.

   a) Homogeneous sphere:

$$P_{\mathrm{SPH}}(q,R) = [F]^2\,,\ F(q,R) = \frac{3(\sin(qR) - qR\cdot\cos(qR))}{(qR)^3} \quad \text{(Supplementary Equation 13)}$$

   b) Homogeneous ellipsoid (semi-axes R, R, εR):

$$P_{\mathrm{ELIP}}(q,R,\varepsilon) = \int_0^{\frac{\pi}{2}} F^2(q,r)\sin\alpha\,\mathrm{d}\alpha \quad \text{(Supplementary Equation 14)}$$

$$r(R,\varepsilon,\alpha) = R(\sin^2\alpha + \varepsilon^2\cos^2\alpha)^{\frac{1}{2}} \quad \text{(Supplementary Equation 15)}$$

The SAXS data are shown in relation to these various models in Supplementary Figure 13, confirming the spherical form of **NT** (Supplementary Figure 13a) and the ellipsoidal form of particles for **A:T=0.14** (Supplementary Figure 13b) in keeping with the predictions of the physical model (see discussion section in main paper and below). No analytical expression being available, a dumbbell form factor based on TEM measurements was generated using a Monte Carlo method (Supplementary

Figure 13c) and found to fit well to the experimental data at high q (Supplementary Figure 13d), whilst some discrepancy in the region $2 \leq u \leq 5$ might be explained by variation in the length and thickness of the particle's central region.

Supplementary Table 3. Summary of LS characterization data for spherical nanoparticle, **NT**, ellipsoids (A:T = 0.14) and dumbbells (A:T = 0.20).

**Sample**	$\langle R_{\mathrm{G}} \rangle_{\mathrm{Z}}$, nm	$\langle R_{\mathrm{H}} \rangle_{\mathrm{Z}}$, nm	$\frac{\langle R_{\mathrm{G}} \rangle_{\mathrm{Z}}}{\langle R_{\mathrm{H}} \rangle_{\mathrm{Z}}}$
**NT**	30.8 ± 1.4	38.2 ± 0.2	0.81
**A:T = 0.14**	40.8 ± 0.9	42.9 ± 0.3	0.95
**A:T = 0.20**	44.9 ± 0.9	45.5 ± 0.3	0.99

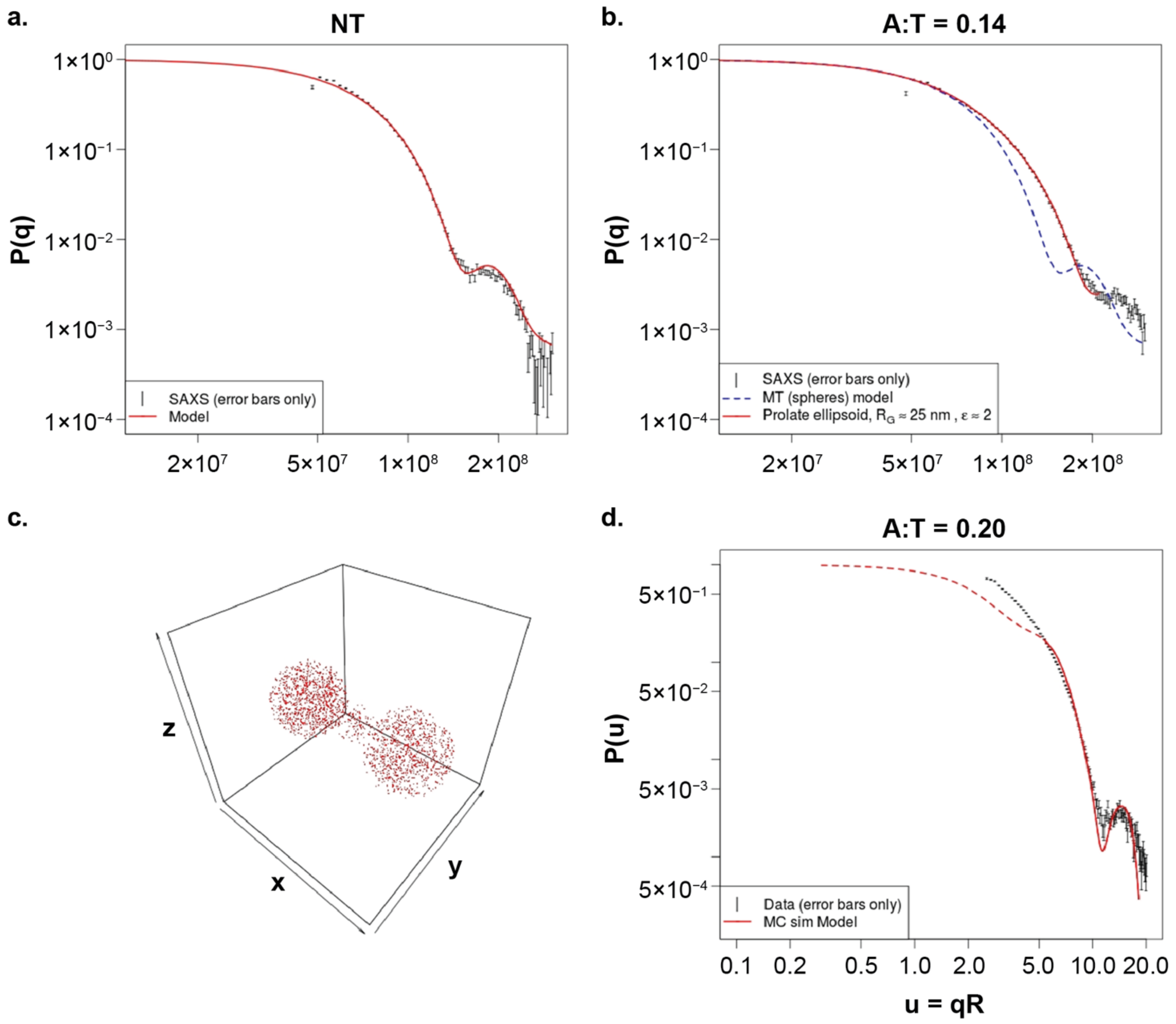

Supplementary Figure 13. SAXS experimental profiles and fittings of: (a) spherical nanoparticles **NT** and (b) nanoparticles with an A:T molar ratio of 0.14, showing the fit to a prolate ellipsoid model. (c) Monte Carlo simulation of the form factor for a dumbbell based on dimensions estimated from TEM images. Pairwise distances were generated either from the enclosed mass to estimate R_{G} and $P_{\mathrm{DUMB}}(q, \boldsymbol{R})$, or from the surface to estimate R_{H}. (d) SAXS experimental profiles and fittings (using Guinier model) of dumbbells at an A:T ratio of 0.20.

Stepwise Morphological Transformation of NT

Morphological Transformation at Low PA Concentrations

We attempted to perform stepwise morphological transformation of the nanoparticles **NT** by adding **PA** in small aliquots (0.07 molar equivalents per addition). However, as shown in Supplementary Figure 14, this resulted only in swelling and partial disassembly of the nanoparticles, rather than shape change.

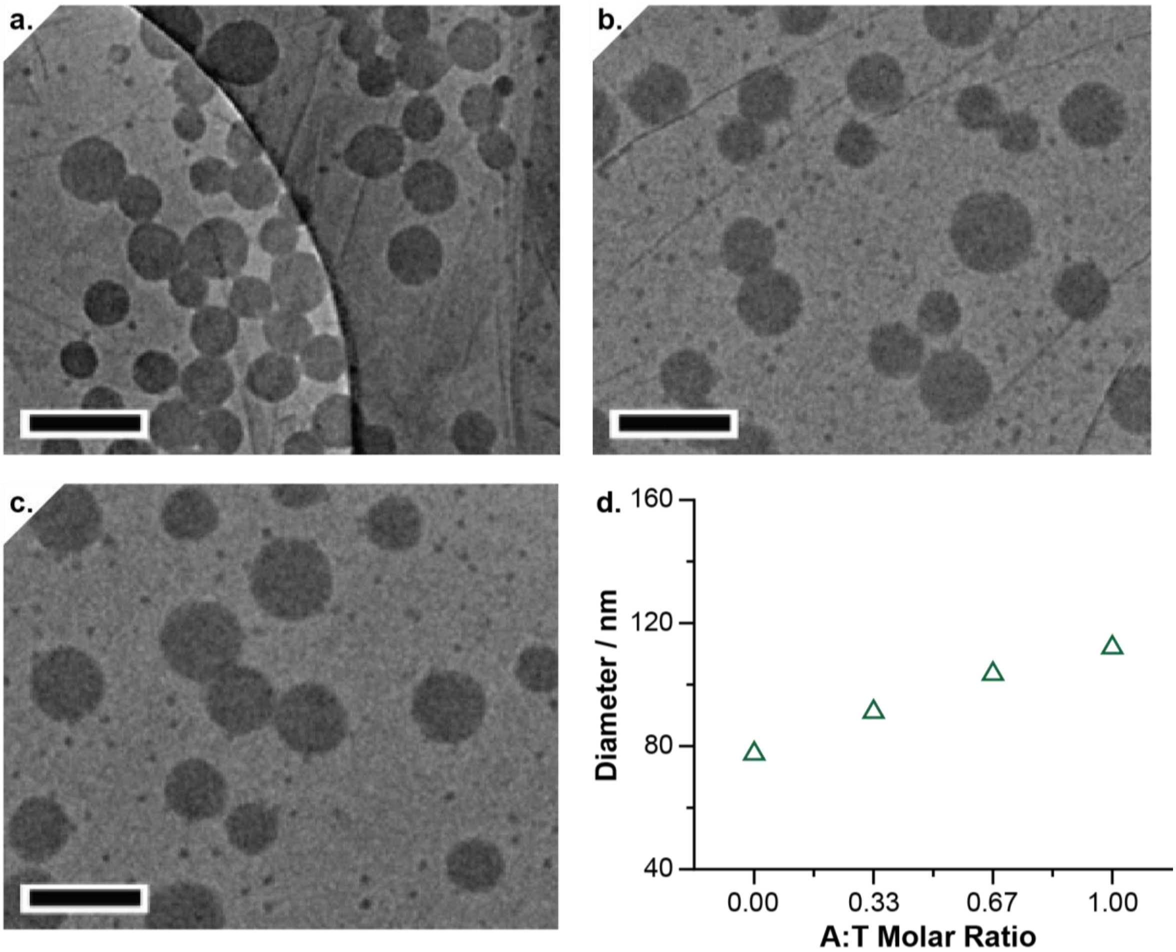

Supplementary Figure 14. TEM images and DLS analyses of nanoparticle **NT** after stepwise addition of low concentrations of **PA** (0.07 molar equivalents relative to thymine per addition) at the following A:T molar ratios: (a) 0.33; (b) 0.67; (c) 1.00; scale bars = 200 nm. (d) Variation of hydrodynamic diameters of the particles shown in (a-c) as measured by DLS.

Stepwise Transformation of Dumbbells

To test the hypothesis that stepwise growth might be possible once anisotropy had been introduced, we first took a sample of the dumbbells (A:T ratio 0.20) and added **PA**, then analysed the resulting particles by TEM (Supplementary Figure 15). As expected, the dumbbells elongated to form worms of around 300 nm length, suggesting that stepwise growth would be possible.

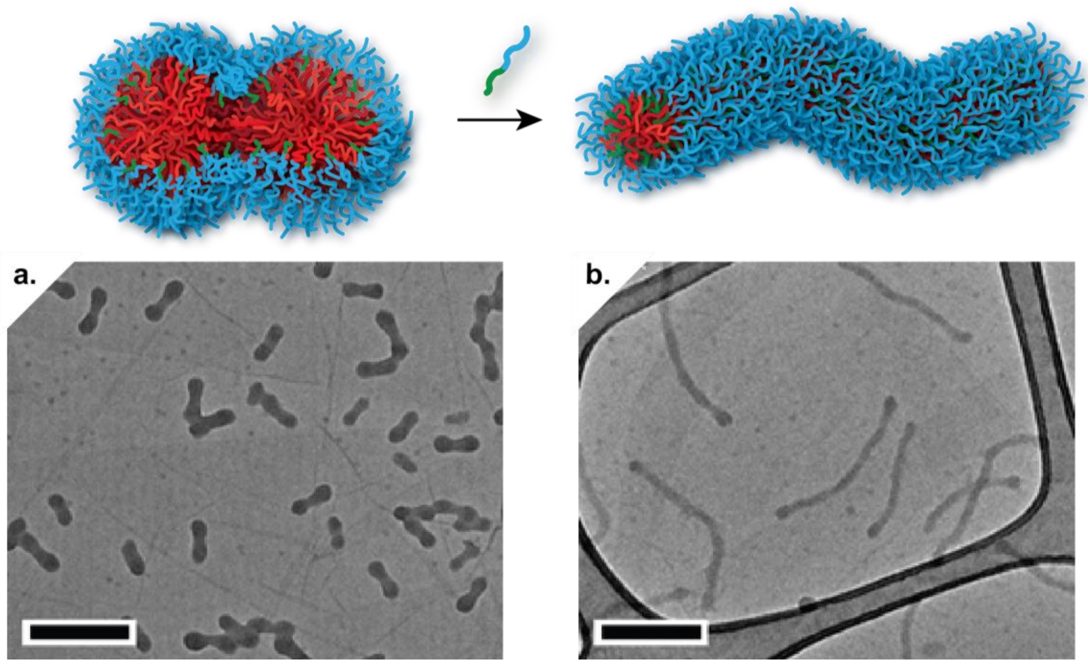

Supplementary Figure 15. Schematic presentation and TEM images of (b) worm-like micelles with lengths over 300 nm formed by further feeding (a) dumbbell-like micelles with **PA** at a total A:T molar ratio of 0.33; scale bars = 200 nm.

Stepwise Growth of Long Wormlike Nanoparticles

Having confirmed that stepwise growth was possible, we moved on to the experiments described in Figure 3 of the main paper. Histograms of the TEM particle counting data are presented in Supplementary Figure 16Supplementary **Figure 17**.

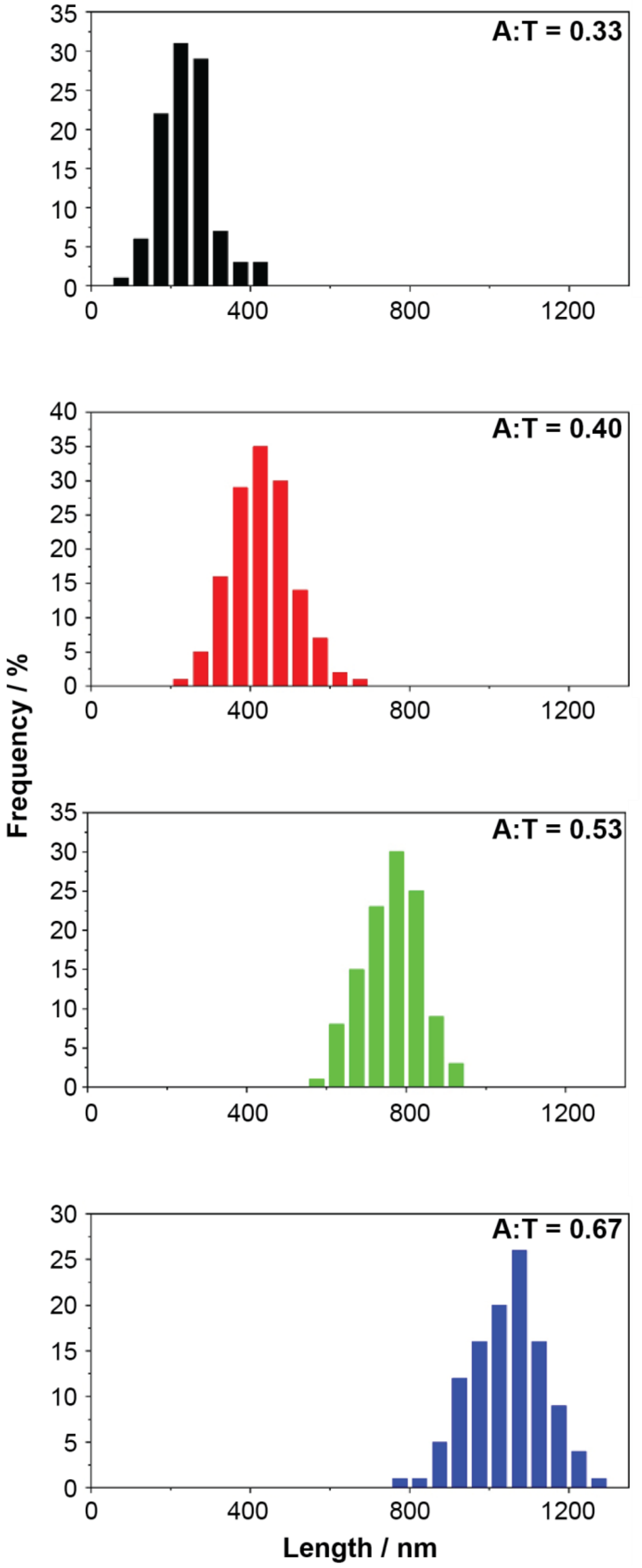

Supplementary Figure 16. Histograms showing the length distributions of the worms grown by stepwise addition of **PA**. Particles were imaged dry on graphene oxide by TEM and measured using ImageJ. The A:T molar ratios in the nanoparticles were: (a) 0.33; (b) 0.40; (c) 0.53; (d) 0.67.

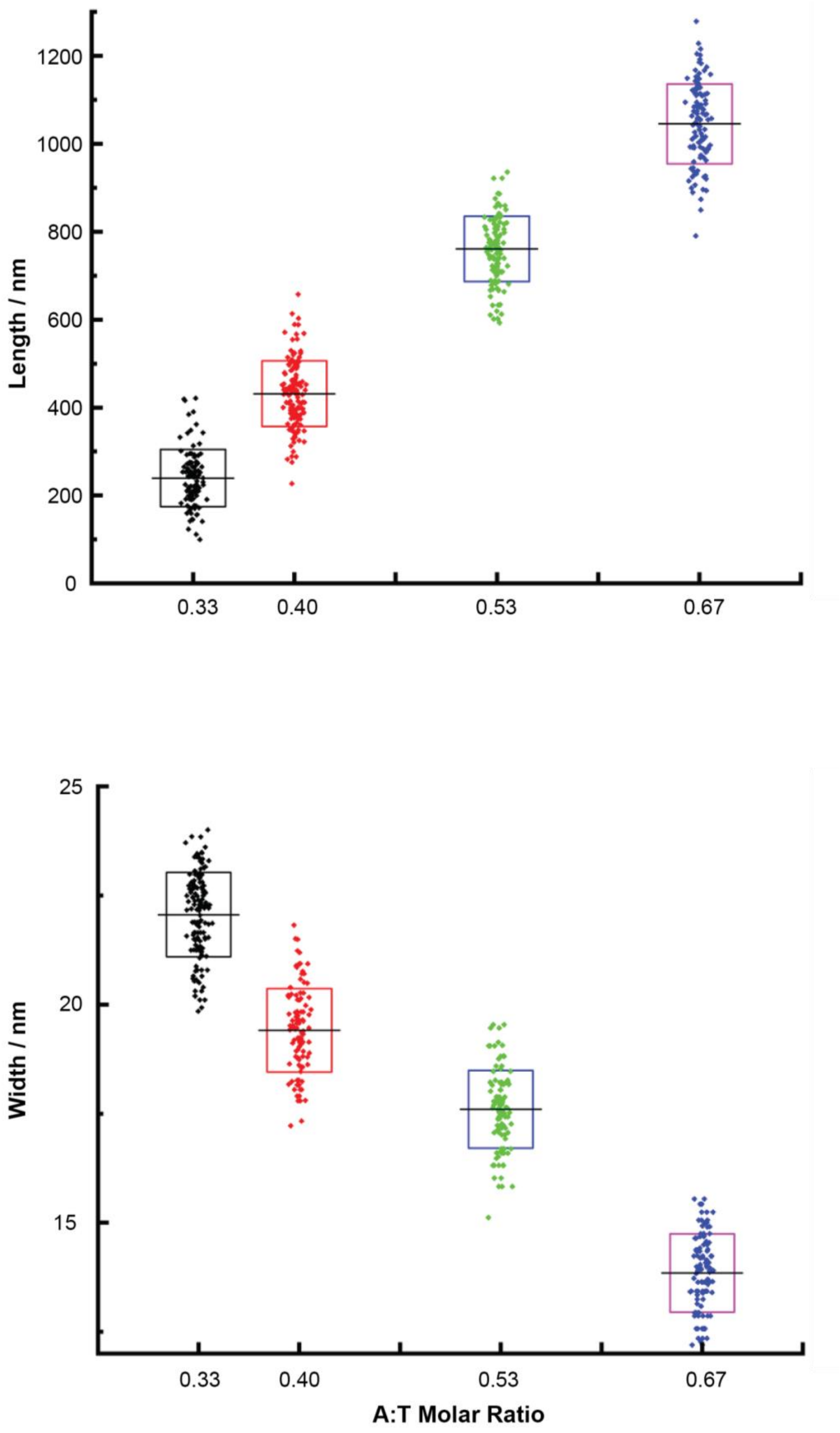

Supplementary Figure 17. Jitter box plot of the (a) lengths and (b) width distributions of the worms fabricated by stepwise growth, as determined by TEM. The central line indicates the mean, and the box encompasses the 95% confidence interval.

SAXS Analysis of Wormlike Nanoparticles

To analyse the wormlike nanoparticles by SAXS we adopted the approach described by Pedersen,[6] which considers the scattering form factor (P_{WORM}) to comprise two components that describe the cross section (P_{CS}) and global particles shape (P_{SH}),

$$P_{\mathrm{WORM}}(q,\boldsymbol{R}) = P_{\mathrm{CS}}P_{\mathrm{SH}} \qquad \text{(Supplementary Equation 16)}$$

We assumed a circular cross-section radius r, i.e.

$$P_{\mathrm{CS}}(q,r) = \left(\frac{2J_1(qr)}{qr}\right)^2 \qquad \text{(Supplementary Equation 17)}$$

where J_1 is a Bessel function of the first kind. For the global shape component, P_{SH}, we assumed a random coil model to be appropriate,

$$P_{\mathrm{SH}}(q,\langle R_{\mathrm{G}}^2\rangle) = \left(\frac{2}{u^2}\right)\cdot[\exp(-u)+u-1] \qquad \text{(Supplementary Equation 18)}$$

$$u = \langle R_{\mathrm{G}}^2\rangle q^2 \qquad \text{(Supplementary Equation 19)}$$

$$\langle R_{\mathrm{G}}^2\rangle = \frac{Lb}{6} \qquad \text{(Supplementary Equation 20)}$$

Observing that the relation $\ln(P_{\mathrm{SH}})\sim\ln(q)$ is approximately linear for the combination of worm lengths, L, apparent by TEM and q range accessible to the SAXS experiment, we further simplified the problem in hand to that of only fitting a two parameter distribution for cross-sectional radius, such that

$$r\sim N(\mu,\sigma) \qquad \text{(Supplementary Equation 21)}$$

The cross-sectional component to the intensity weighted average scattering form factor was then approximated by a sum for values r_i that were sampled from the parameterised distribution:

$$P_{\mathrm{CS}}(q,\langle r\rangle_{\mathrm{Z}}) \approx \frac{\sum_i n_i w_i^2 P_{\mathrm{CS}}(q,r_i)}{\sum_i n_i w_i^2} \qquad \text{(Supplementary Equation 22)}$$

where,

$$w_i \propto r_i^2 \qquad \text{(Supplementary Equation 23)}$$

In Supplementary Figure 18, the raw SAXS data are shown in the left hand panel along with fits to the full form factor ($P_{SH} \cdot P_{CS}$). The middle panel of Supplementary Figure 18 shows the result of dividing the raw data by P_{SH}, and the fit to P_{CS}. The widths extracted from the P_{CS} fit are summarised in Figure 3g (main article) and in Supplementary Table 4.

TEM and SAXS measurements for worm cross-section were analysed by linear regression with the method of measurement, 'TEM' or 'SAXS', incorporated as a factor. According to the fitted regression model, there is very strong evidence to suggest that for each increase in the A:T molar ratio there is a corresponding decrease in worm diameter and this is a statistically significant effect ($F(3,4) = 41.1$, R-squared = 0.969). On average, an increase in A:T molar ratio of 0.1 is associated with a 2.3 nm decrease in worm diameter ($t = -5.92$, $p = 0.004$). There is no statistical evidence to suggest that the trend in the data is different according to the method of measurement ($t = 0.189$, $p = 0.860$).

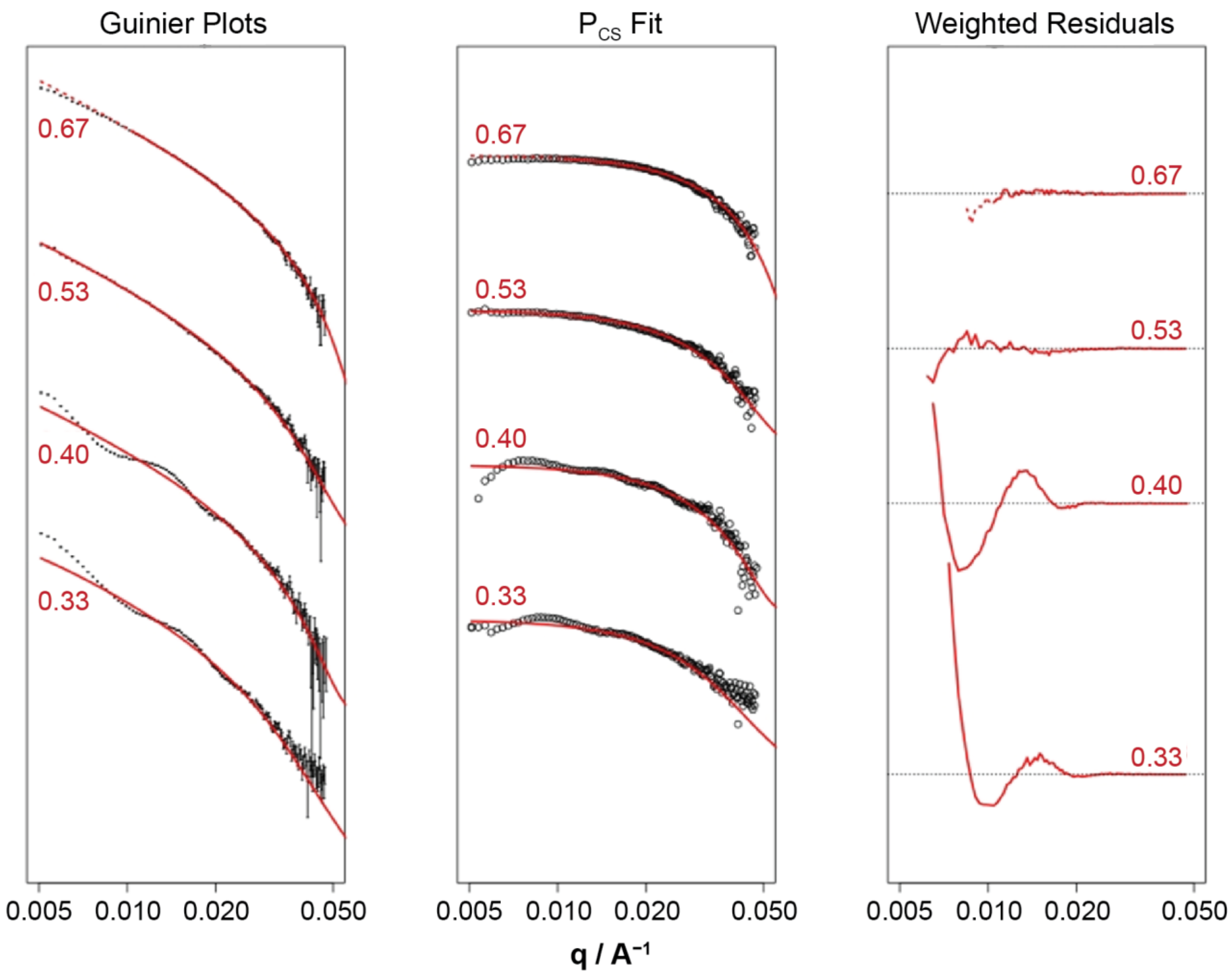

Supplementary Figure 18. Estimation of radius, *R*, from SAXS data, for four samples of worms grown to different mean lengths. Note that in each of the above figures the data are offset for clarity. (a) Guinier plots of the raw SAXS data and the fit $I(q) / P_{SH} \cdot P_{CS}$. (b) Plots of the SAXS data following removal of the P_{SH} component, with P_{CS} fits to estimate radius by variation of two parameters, assuming that $R \sim N(\mu R; \sigma R)$. (c) Weighted residuals, normalized to absolute scattering intensity per sample for comparison. Numbers in red indicate the A:T molar ratio in the nanostructures.

Supplementary Table 4. Summary of SAXS characterization data for cross-sectional dimensions of worms of different lengths. N.B. These results are presented in Figure 3g as diameter, rather than radius, for direct comparison with the measurements from TEM images.

**Sample**	$\mu_r = \langle r \rangle_N$ / nm	σ_r / nm
**A:T = 0.33**	10.4	2.0
**A:T = 0.40**	9.8	1.9
**A:T = 0.53**	8.9	1.0
**A:T = 0.67**	6.5	0.8

Worm Disassembly at High A:T Ratios

Addition of **PA** above A:T ratios of 1.00 was observed to cause disassembly of the nanoparticles into short worms and spheres, as shown in the TEM image in Supplementary Figure 19.

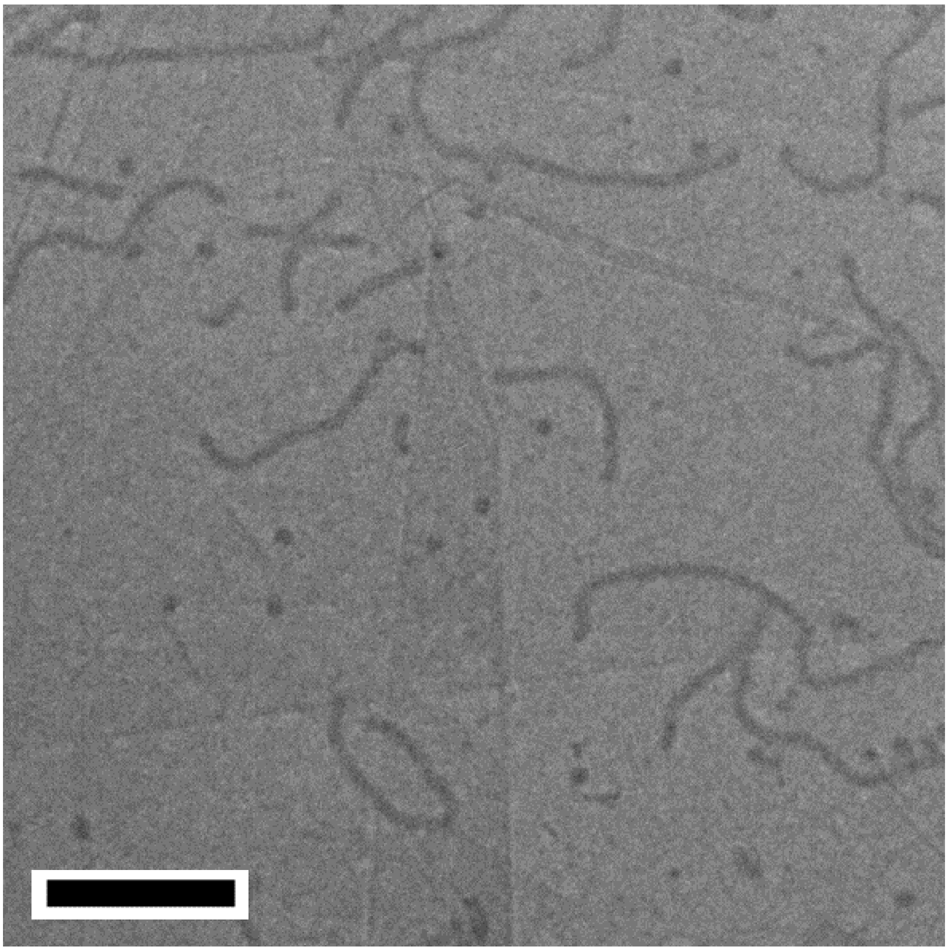

Supplementary Figure 19. TEM image of nanoparticles with an A:T ratio of 1:1 following further addition of **PA**, showing the resulting disassembly into a mixture of short worms and spherical nanoparticle; scale bar = 200 nm.

Morphological Transformation Control Experiments

Analysis of Mixtures of NT with $\mathbf{PA^{Me}}$, PT1 and PS

In order to confirm that strong H-bonding between A and T was necessary to drive the morphological transformation process, we performed a series of control experiments using polymers in which H-bonding was either partially blocked or completely removed. We began by synthesising four polymers: $PNAM_{39}$-*b*-$PMAAm_{20}$ ($\mathbf{PA^{Me}}$), $PNAM_{39}$-*b*-$PTAm_{20}$ (**PT1**), $PNAM_{39}$-*b*-PSt_{20} (**PS**) and $PNAM_{39}$-*b*-$PMT^{Me}Am_{300}$ ($\mathbf{PT^{Me}}$). These were all synthesised from the $PNAM_{39}$ macroCTA whose synthesis is described in the Supplementary Methods. Their characterisation data can be found in Supplementary Table 2. $\mathbf{PA^{Me}}$ was not expected to form strong H-bonds with **NT** because of methylation of the adenine nitrogen; **PT1** was not expected to form strong bonds with **NT** because thymine does not self-dimerise under normal conditions; and **PS** was not expected to form strong bonds with **NT** because of the absence of any H-bond donors or acceptors. $\mathbf{PA^{Me}}$, **PT1** and **PS** were then mixed with separate solutions of **NT** at different molar ratios, and the resulting nanoparticles analysed by DLS and TEM. DLS analyses (Supplementary Figure 20) showed no significant changes in the hydrodynamic diameters of the nanoparticles, and TEM analyses showed no noticeable changes in morphology (Supplementary Figure 21).

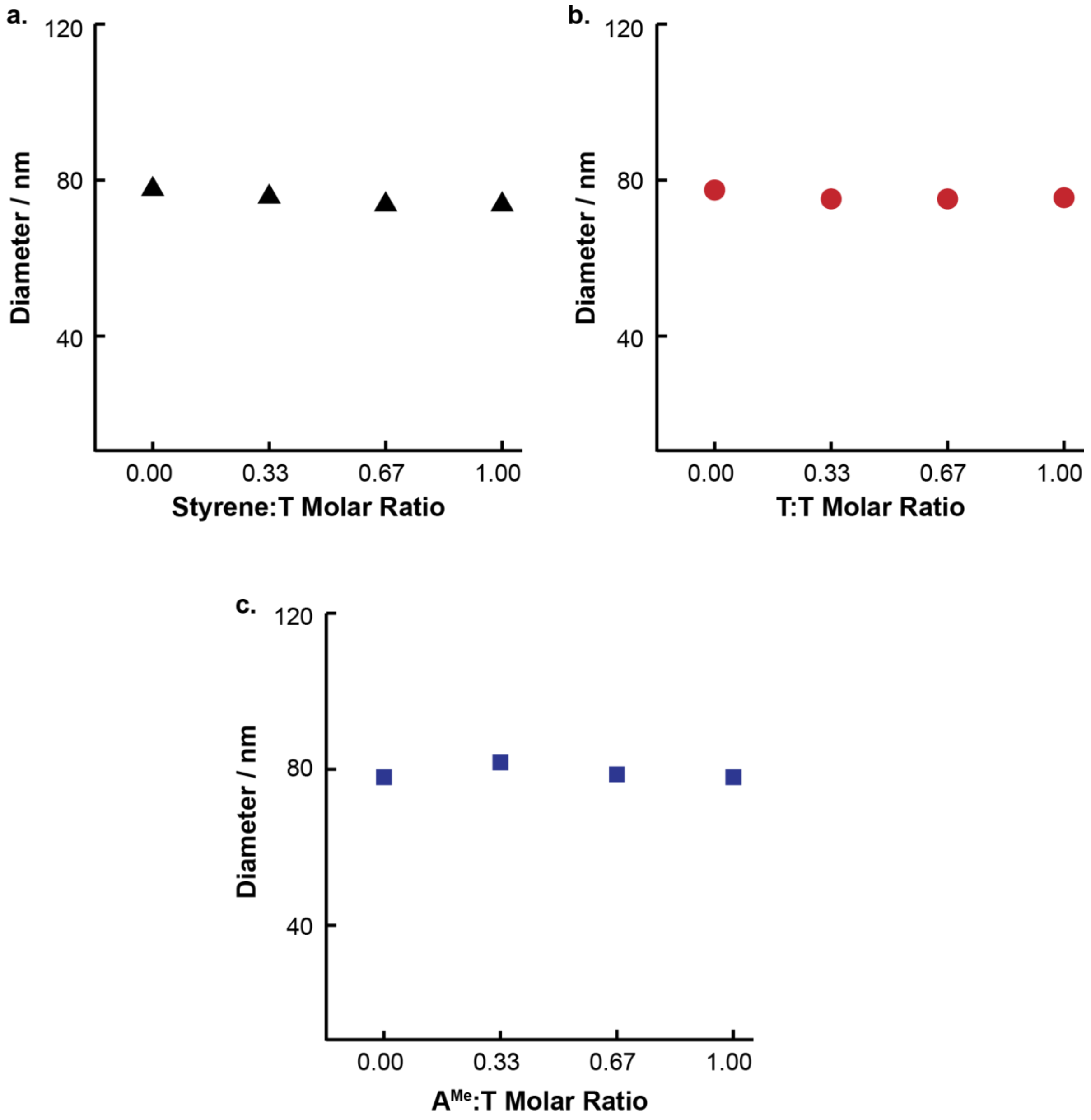

Supplementary Figure 20. Variation of hydrodynamic diameter of the mixture of **NT** with non-complementary copolymers as determined by DLS analyses. (a) Nanoparticle **NT** with **PS**; (b) **NT** with **PT1**; (c) **NT** with **PA^{Me}**; Error bars represent standard deviation of at least three measurements.

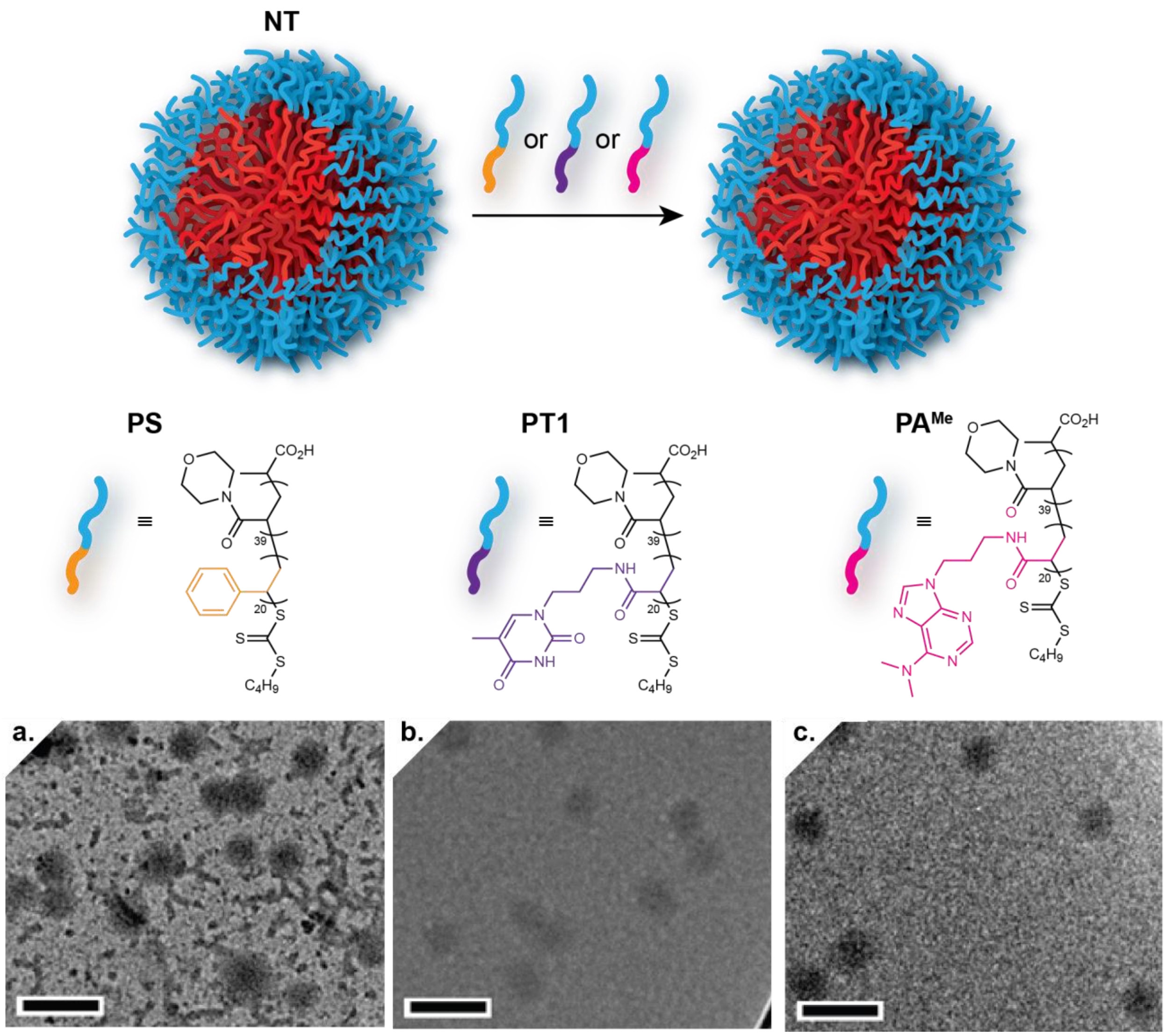

Supplementary Figure 21. Dry-state TEM images on graphene oxide of **NT** following additions of **PS** (a), **PT1** (b) and **PA**$^{\mathbf{Me}}$ (c) at the following molar ratios: (a) **St:T** = 1.00; (b) **T:T** = 1.00; (c) **A**$^{\mathbf{Me}}$**:T** = 1.00; Small **PS** aggregates were observed when mixing **PS** with **NT**; scale bars = 200 nm.

Analysis of the Aggregation Behaviour of PA^{Me}, PT1, PS and PA

We wanted to know whether any of the added polymers formed aggregates in solution, which might complicate their interaction with **NT**. We therefore investigated 0.5 mg mL^{-1} solutions of **PA**$^{\mathbf{Me}}$, **PT1**, **PS** and **PA** by DLS. DLS analyses (Supplementary Figure 22) showed that all polymers formed very small aggregates in water at this concentration, but when diluted to the concentrations used in the addition experiments (below 0.1 mg mL^{-1}) no particles were observable by light scattering, except in

the case of **PS**. We therefore concluded that in the additions experiments the polymers **PA^{Me}**, **PT1**, and **PA** were present as unimers. The **PS** aggregates were further investigated by TEM (Supplementary Figure 23), and observed as small nanoparticles, which were also visible in the TEM images of mixtures of **PS** with **NT** (Supplementary Figure 21a).

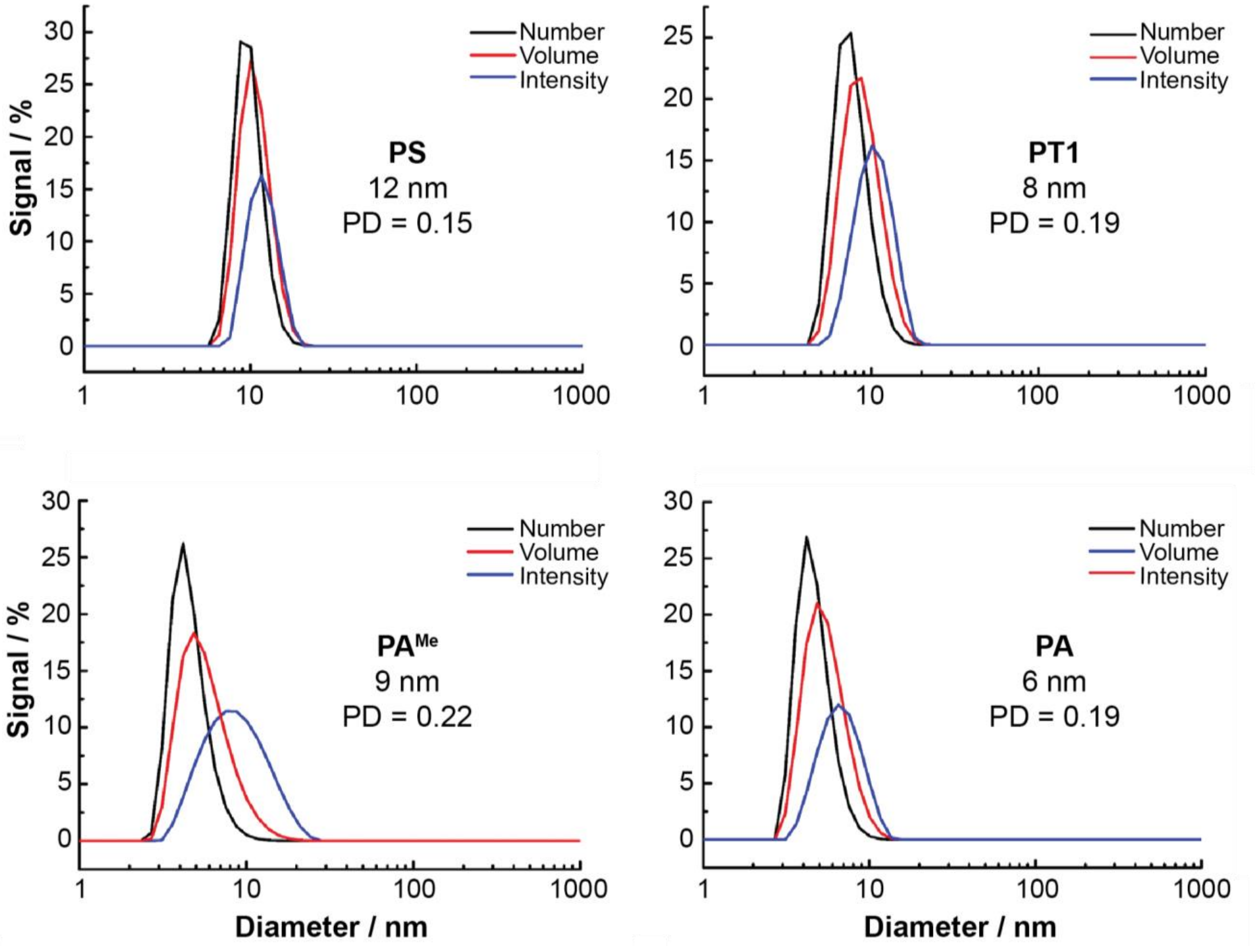

Supplementary Figure 22. DLS analyses of small aggregates (0.5 mg mL^{-1}) formed in water by (a) **PS**; (b) **PT1**; (c) **PA^{Me}**, (d) **PA**.

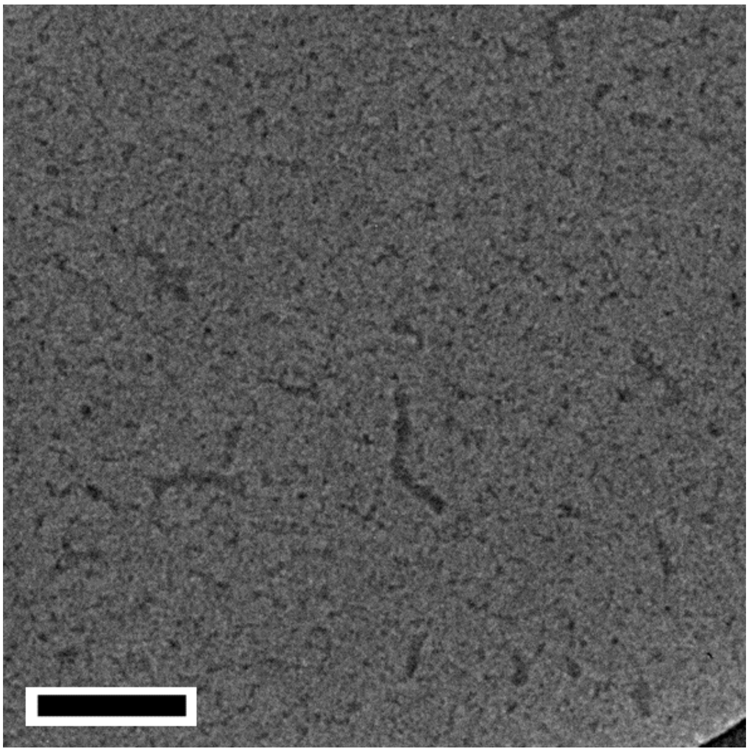

Supplementary Figure 23. TEM images of **PS** aggregates formed in water; scale bar = 200 nm.

Blocking of H-Bonding in the Nanoparticle

We then moved on to investigate the effect of partially blocking H-bonding in the nanoparticle. This was achieved by synthesising a methylated thymine monomer (**T^{Me}Am**, see section Supplementary Methods for synthesis details) and polymerising it with the PNAM$_{39}$ macroCTA to make a methylated analogue of **PT**: **PT^{Me}**. The synthesis of **PT^{Me}** was conducted using the general procedure given in section the Supplementary Methods, and its characterisation data can be found in Supplementary Table 2. **PT^{Me}** was then self-assembled using the protocol in the Supplementary Methods to give spherical nanoparticle **NT^{Me}**, which was analysed by DLS and TEM (Supplementary Figure 24). **PA** was then added to **NT^{Me}** at different A:T molar ratios and the resulting particles investigated by DLS and TEM (Supplementary Figure 25). No change in hydrodynamic diameter or morphology was observed, providing further evidence that strong H-bonding between the nanoparticle core and the added polymer was necessary for morphological transformation to occur.

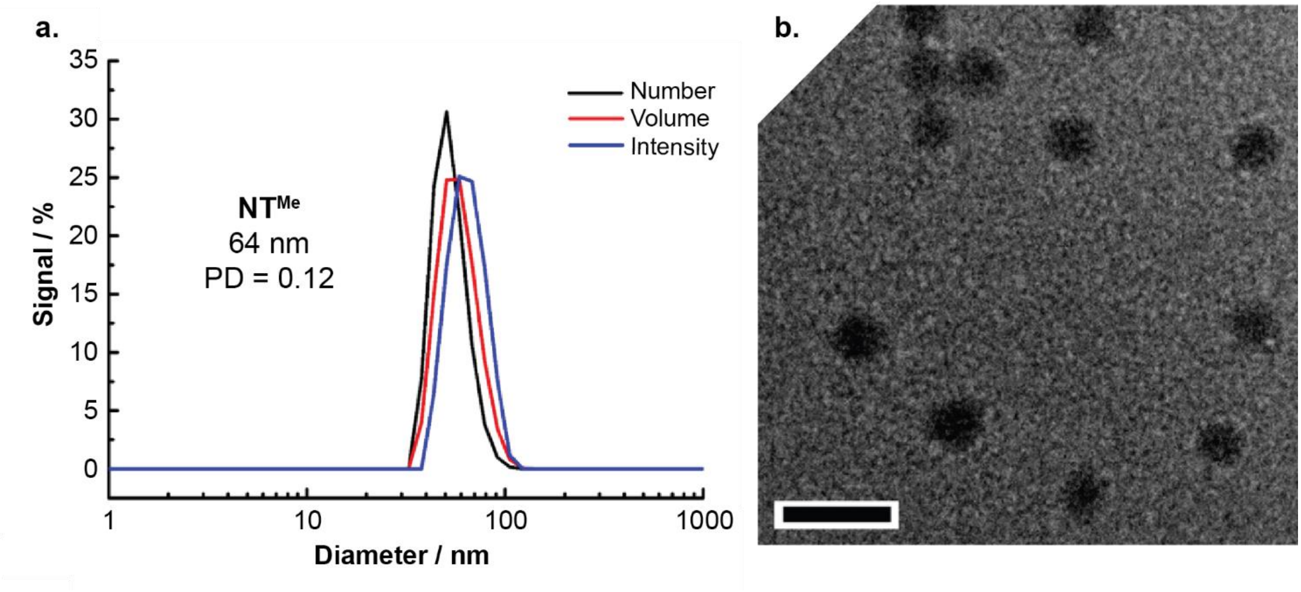

Supplementary Figure 24. (a) DLS analysis of nanoparticle **NT^{Me}** (0.5 mg mL^{-1}) in water; (b) TEM images of **NT^{Me}**; scale bar = 200 nm.

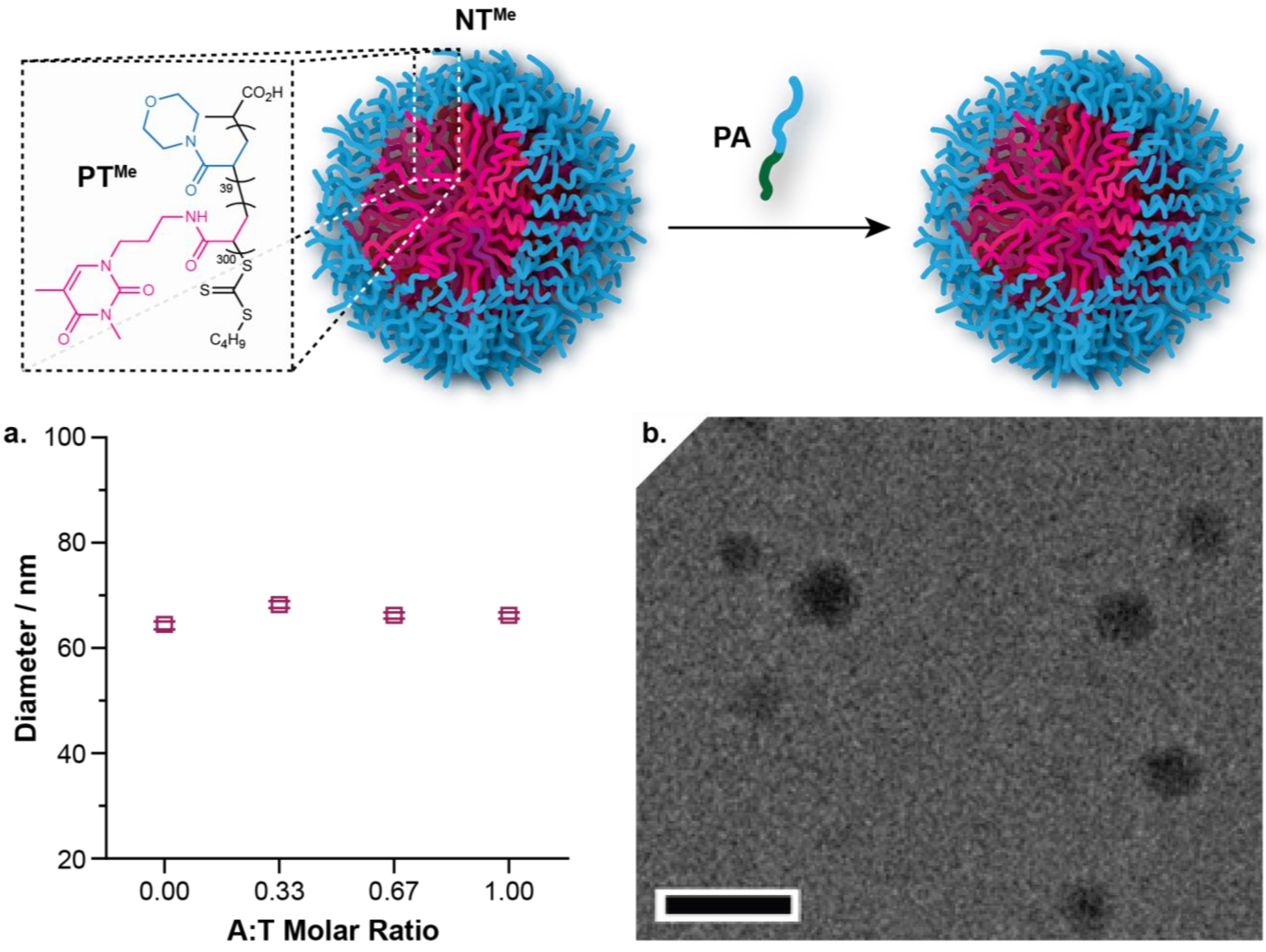

Supplementary Figure 25. (a) Variation of hydrodynamic diameter of the mixture of **NT^{Me}** with **PA** as determined by DLS analyses; Error bars represent standard deviation of at least three measurements. (b) dry-state TEM images of nanoparticles **NT^{Me}** following addition of **PA** at an A:T^{Me} molar ratio of 1:1; scale bar = 200 nm.

Self-Assembly by Solvent Switch from a Common Solvent

To investigate whether the morphological transformation products represented the thermodynamic assembly products, we mixed **PA** and **PT** in the appropriate ratios and performed a slow solvent switch from DMF (a common solvent for all blocks) to water. As shown in Supplementary Figure 26, only small spheres were observed, with no apparent formation of anisotropic morphologies.

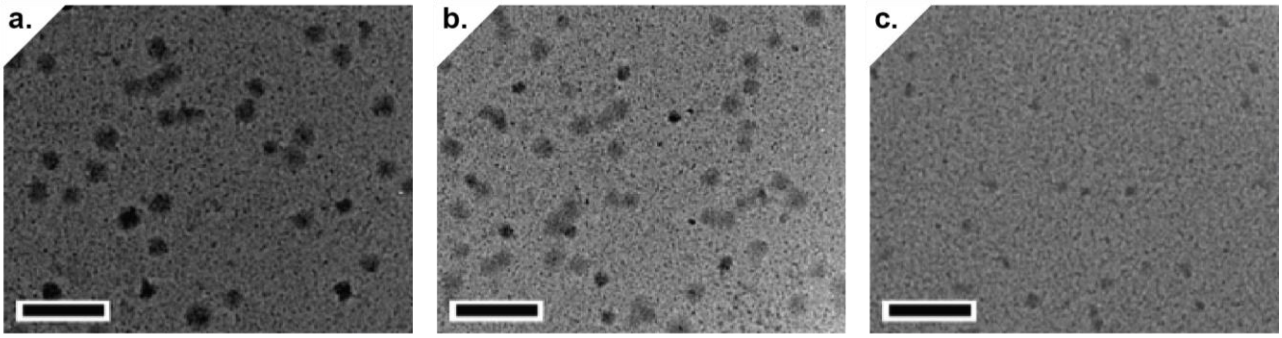

Supplementary Figure 26. TEM images of nanoparticles consisting of **PA** and **PT** prepared through a solvent switch method from DMF to H_2O at the following molar ratios of A:T: (a) 0.33; (b) 0.67; (c) 1.00; scale bars = 200 nm. The sizes of the spherical nanoparticles were 49 ± 5 nm, 42 ± 4 nm and 30 ± 5 nm, respectively.

MicroDSC Measurements

We used microDSC to look for evidence of any crystallinity in the anisotropic nanoparticles. A typical thermogram is shown in Supplementary Figure 27 – the absence of any significant thermal events was consistent with the sample being amorphous.

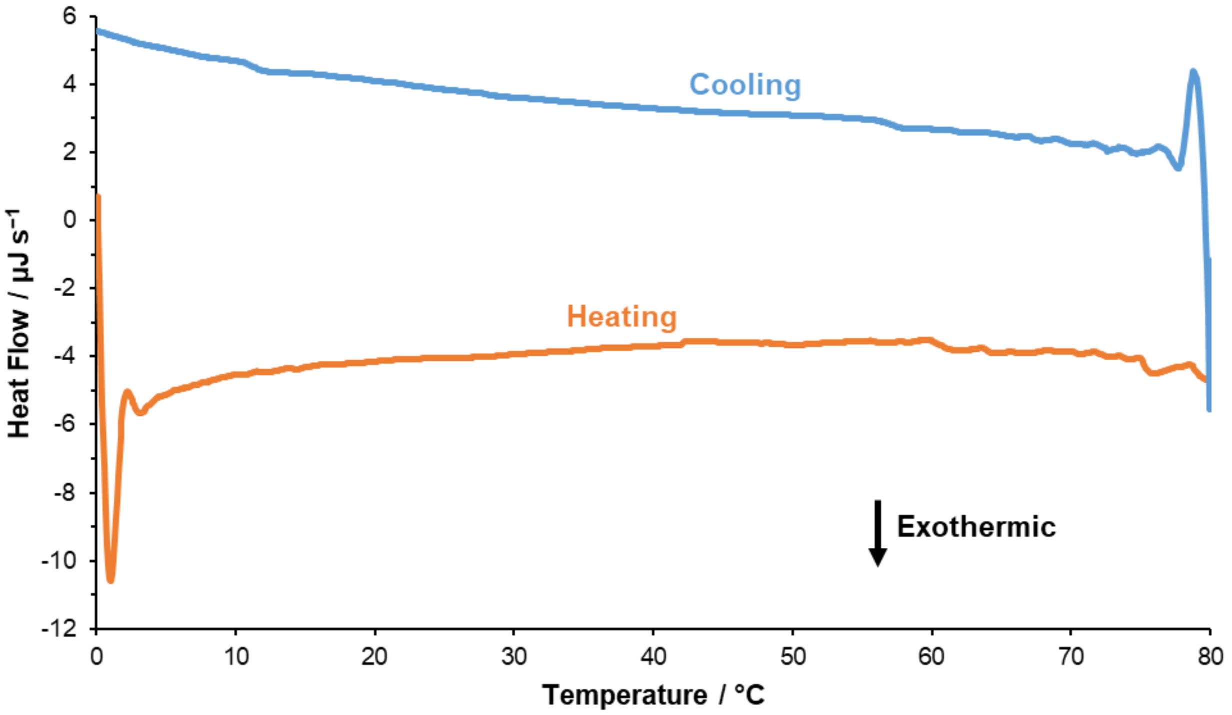

Supplementary Figure 27. MicroDSC thermagrams of a 0.7 mg mL^{-1} solution of **NT/PA** at an A:T ratio of 0.67.

Experiments Confirming Single Particle Transformation Process

SLS Analyses to Determine Nanoparticle Molecular Weights

LS was used to compare estimates for the mass average molar mass of **NT** spherical particles and **A:T = 0.20** dumbbells. Additional filtration of the samples through a 220 nm or 450 nm pore size was found to reduce the sample concentration by up to 30% but allowed estimation of $\langle R_\mathrm{G} \rangle_\mathrm{Z}$ from data over the full angular range and these results were used to constrain the estimation of $\bar{M}_\mathrm{W}$ from samples filtered at 1.2 μm, for which no reduction in the sample concentration could be discerned. Consequently, the Zimm plots in Supplementary Figure 28Supplementary **Figure 29** show that a subset of the data ($80 \leq \theta \leq 130°$) was used in each case to estimate $\bar{M}_\mathrm{W}$, the deviance at small angles usually being associated with dust or aggregates. Results are summarised in Supplementary Table 5.

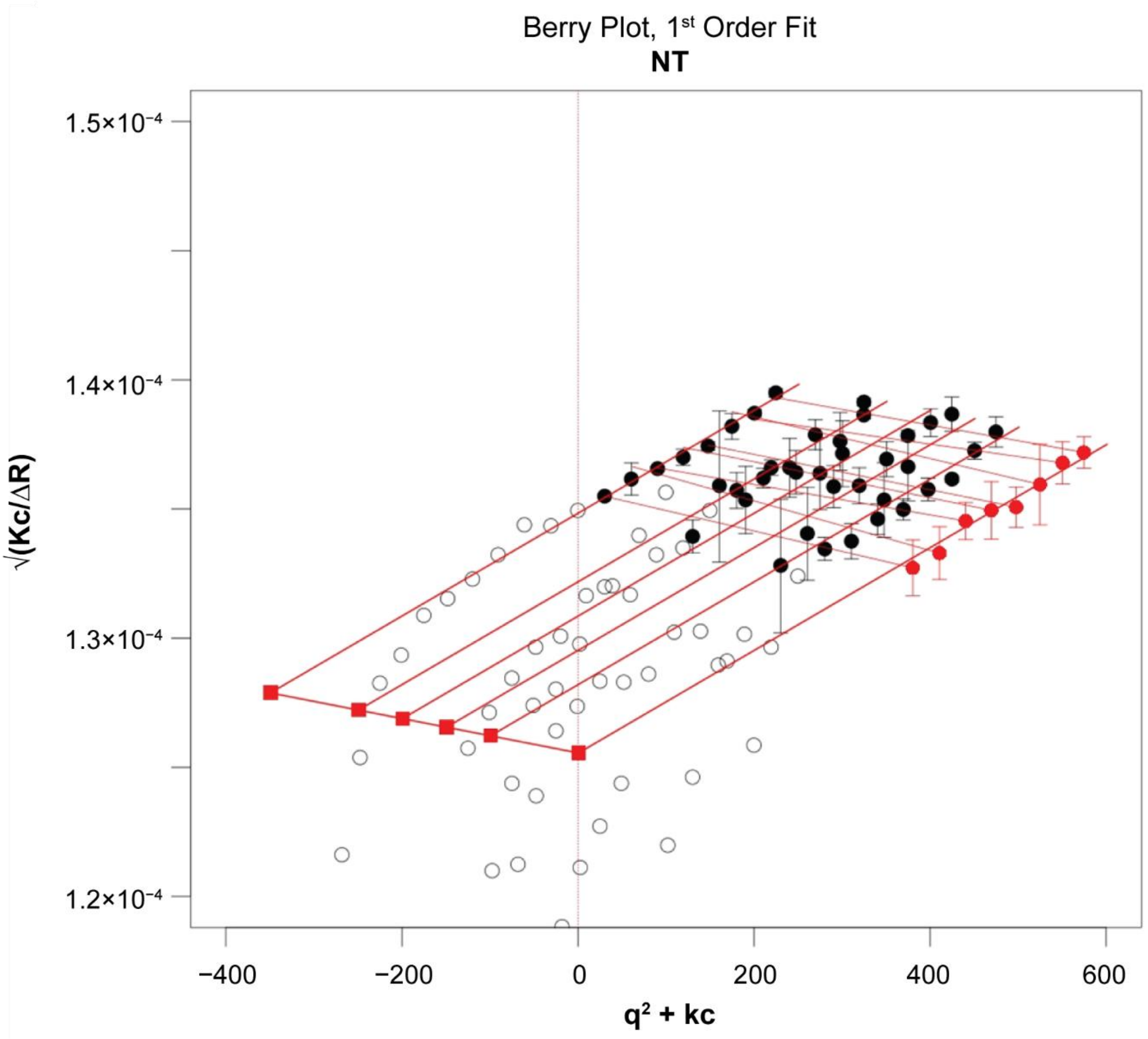

Supplementary Figure 28. Zimm plot (Berry transformation) of spherical nanoparticles **NT**, which gives M_w = 63.4 ± 0.9 × 10^6 Da. The sample concentrations were 0.2, 0.3, 0.4, 0.5 and 0.7 mg mL^{-1}, respectively.

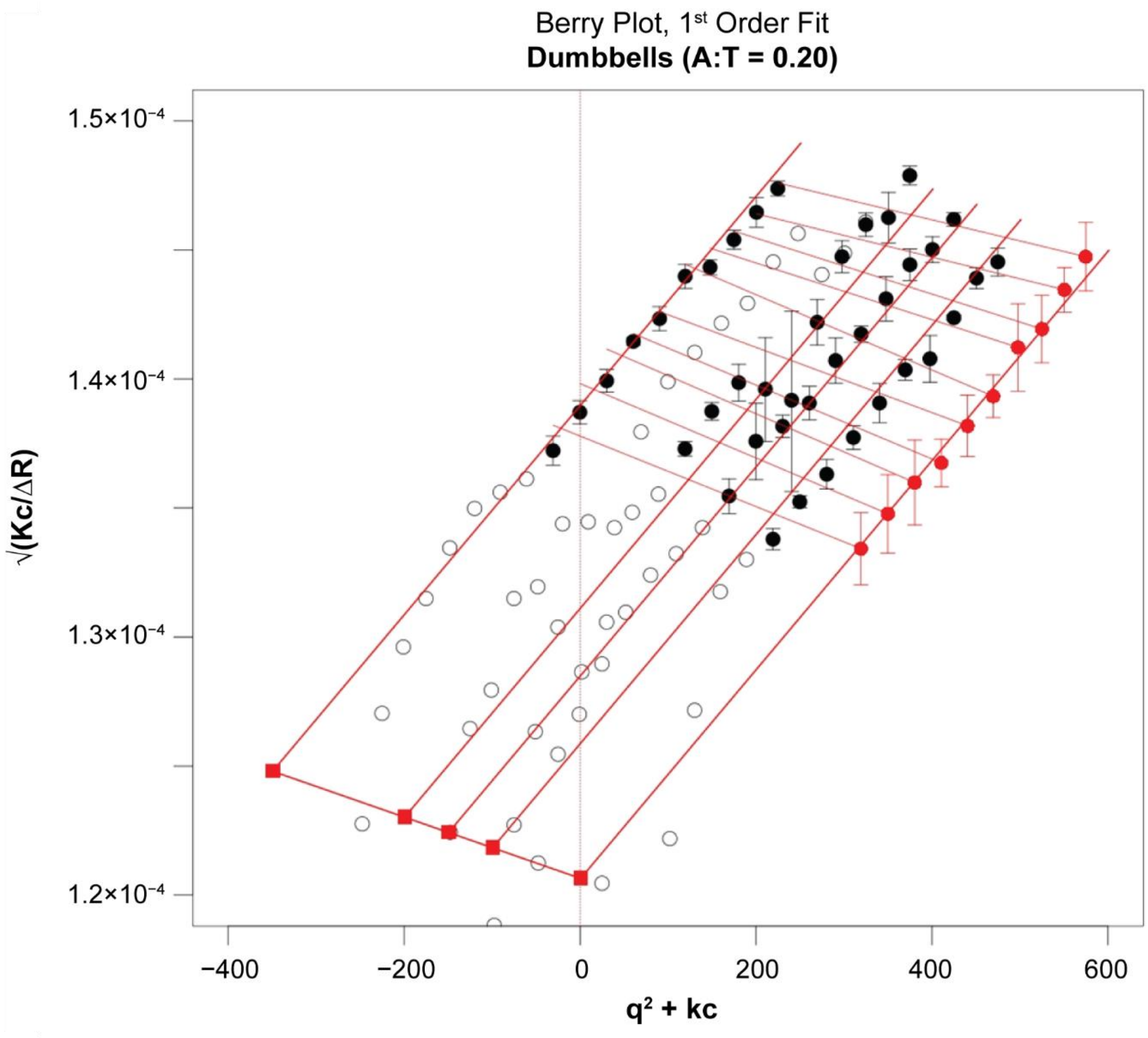

Supplementary Figure 29. Zimm plot (Berry transformation) of dumbbells with an A:T molar ratio of 0.20, which gives $M_w = 68.7 \pm 0.9 \times 10^6$ Da. The sample concentrations were 0.2, 0.3, 0.4, 0.5 and 0.7 mg mL^{-1}, respectively.

Supplementary Table 5. Summary of SLS characterization data for spherical nanoparticle **NT** and dumbbells with an A:T molar ratio of 0.20.

Sample	M_w / Da	R_g / nm	R_h / nm	R_g/R_h
**NT**	$63.4 \pm 0.9 \times 10^6$	30.8	38.2	0.81
**Dumbbells (A:T 0.20)**	$68.7 \pm 0.9 \times 10^6$	44.9	45.5	0.99

AFM Analyses of Nanoparticles

We imaged the seed nanoparticles (**NT**), dumbbells, and a sample of the worms using AFM (Supplementary Figure 30). There was a consistent decrease in the height profile of individual particles across the series, which provided further evidence for a single particle transformation process (particle–particle fusion was expected to produce structures with similar height profiles).

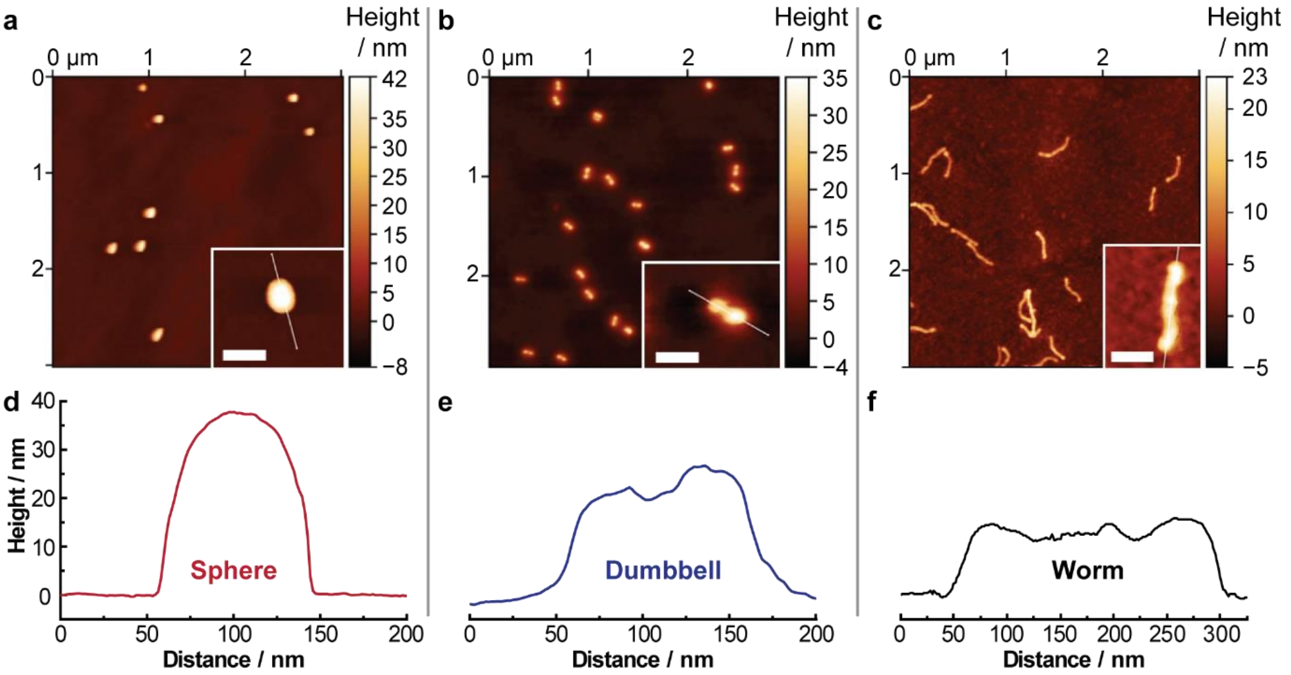

Supplementary Figure 30. AFM images and the corresponding height profiles of (a, d) spherical nanoparticles **NT**, (b, e) dumbbell-like micelles at an A:T molar ratio of 0.20 and (c, f) worm-like micelles formed by further adding **PA** into dumbbells at a total A:T molar ratio of 0.33; scale bars = 100 nm.

The Effect of Altering Polymer Block Lengths on MORPH

Synthesis of PA with Different Block Lengths

A series of analogues of **PA** with different lengths of the PNAM and PAAm blocks were synthesised using the procedures detailed in the Supplementary Methods. Their properties are given in Supplementary Table 6.

Supplementary Table 6. Properties of the **PA** analogues synthesised to investigate the effect of block length on the MORPH mechanism.

Polymer	$M_{n,NMR}$* / kDa	$M_{n,SEC}$† / kDa	$Đ_M$†
$PNAM_{96}$	13.8	13.9	1.06
$PNAM_{295}$	41.9	35.0	1.11
$PNAM_{96}$-*b*-$PAAm_{20}$	18.6	19.9	1.08
$PNAM_{295}$-*b*-$PAAm_{20}$	47.0	36.8	1.17
$PNAM_{39}$-*b*-$PAAm_{10}$	8.2	10.6	1.04
$PNAM_{39}$-*b*-$PAAm_{30}$	13.1	16.4	1.12

* Determined by ^{1}H NMR spectroscopy (400 MHz) in deuterated DMSO. † Determined by DMF SEC, with poly(methyl methacrylate) (PMMA) standards.

Effect of Increasing the Length of the Corona Block

We investigated the effect of increasing the length of the corona block in the added polymer by performing addition experiments using the same seed nanoparticle (**NT**) and either $PNAM_{96}$-*b*-$PAAm_{20}$ or $PNAM_{295}$-*b*-$PAAm_{20}$. The MORPH process was followed by both DLS and TEM (Supplementary Figure 31). As discussed in the main text, increasing the length of the PNAM corona block had a marked effect on the MORPH process, with quicker progression the longer the block.

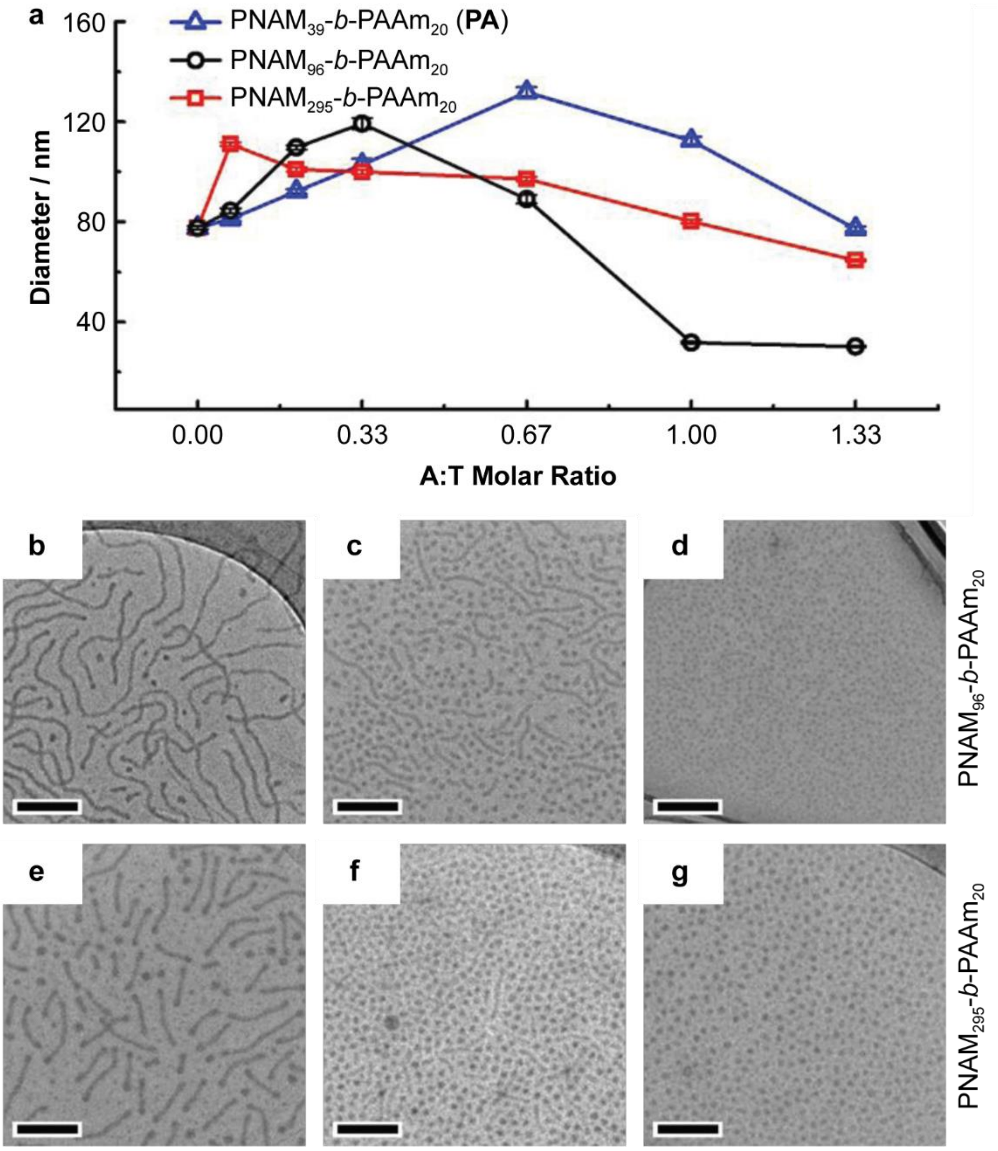

Supplementary Figure 31. The effect of altering the NAM block length on the MORPH process. (a) DLS diameters of nanoparticles **NT** following addition of different molar ratios of the polymers PNAM$_{39}$-*b*-PAAm$_{20}$ (**PA**), PNAM$_{96}$-*b*-PAAm$_{20}$ and PNAM$_{295}$-*b*-PAAm$_{20}$. Note that because of the non-spherical nature of the worms these data are intended to give only a qualitative indication of the differences. (b-d) Dry state TEM images of **NT** following addition of PNAM$_{96}$-*b*-PAAm$_{20}$ at A:T molar ratios of 0.33 (b), 0.67 (c) and 1.00 (d). (e-g) Dry state TEM images of **NT** following addition of PNAM$_{295}$-*b*-PAAm$_{20}$ at A:T molar ratios of 0.33 (e), 0.67 (f) and 1.00 (g). Scale bars = 200 nm.

Effect of Varying the Length of the Adenine-Containing Block

We investigated the effect of varying the length of adenine-containing block by performing addition experiments using the same seed nanoparticle (**NT**) and either $PNAM_{39}$-*b*-$PAAm_{10}$ (with a shorter adenine-containing block) or $PNAM_{39}$-*b*-$PAAm_{30}$ (with a longer adenine-containing block). The MORPH process was followed by DLS and TEM (Supplementary Figure 32). As discussed in the main text, decreasing the length of the adenine-containing block abolished anisotropic growth completely, while increasing it led to slower progression through the MORPH pathway.

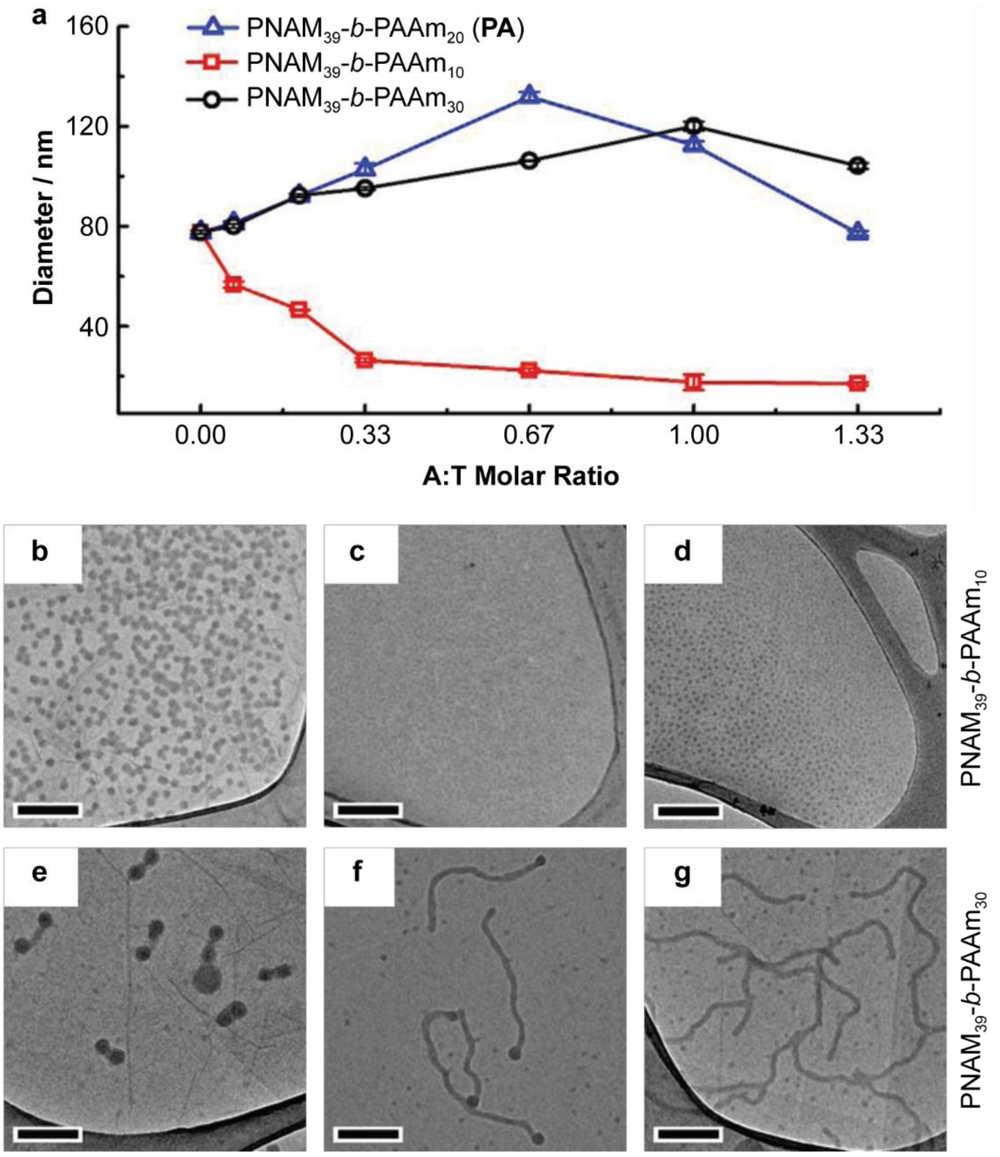

Supplementary Figure 32. The effect of altering the adenine-containing block length on the MORPH process. (a) DLS diameters of nanoparticles **NT** following addition of different molar ratio of the polymers PNAM$_{39}$-*b*-PAAm$_{20}$ (**PA**), PNAM$_{39}$-*b*-PAAm$_{10}$ and PNAM$_{39}$-*b*-PAAm$_{30}$. Note that because of the non-spherical nature of the worms these data are intended to give only a qualitative indication of the differences. (b-d) Dry state TEM images of **NT** following addition of PNAM$_{39}$-*b*-PAAm$_{10}$ at A:T molar ratios of 0.33 (b), 0.67 (c) and 1.00 (d). (e-g) Dry state TEM images of **NT** following addition of PNAM$_{39}$-*b*-PAAm$_{30}$ at A:T molar ratios of 0.33 (e), 0.67 (f) and 1.00 (g). Scale bars = 200 nm.

Fluorescent Tagging Using Morphological Transformation

Syntheses of Fluorescently-Labelled PA

Samples of **PA** tagged with a green (**PA^{G}**) or red (**PA^{R}**) dye were synthesised as shown in Supplementary Figure 33 by modifying the carboxylic acid end group of **PA** with amine-containing dye molecules. Successful dye incorporation was confirmed by ^{1}H NMR spectroscopy (Supplementary Figure 34).

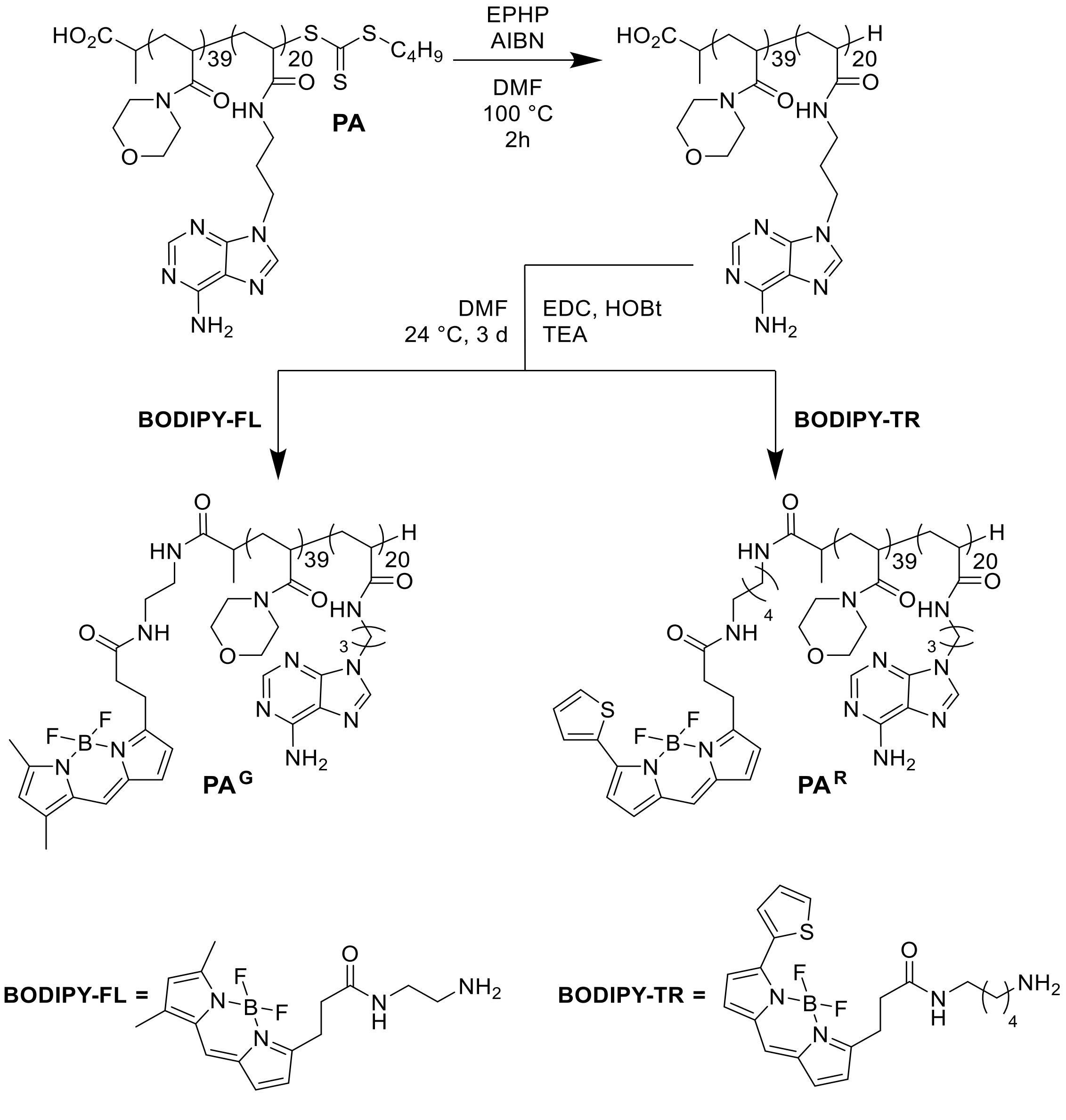

Supplementary Figure 33. Syntheses of **PA^{G}** ((BODIPY-FL)-PNAM$_{39}$-*b*-PAAm$_{20}$) and **PA^{R}** ((BODIPY-TR)-PNAM$_{39}$-*b*-PAAm$_{20}$).

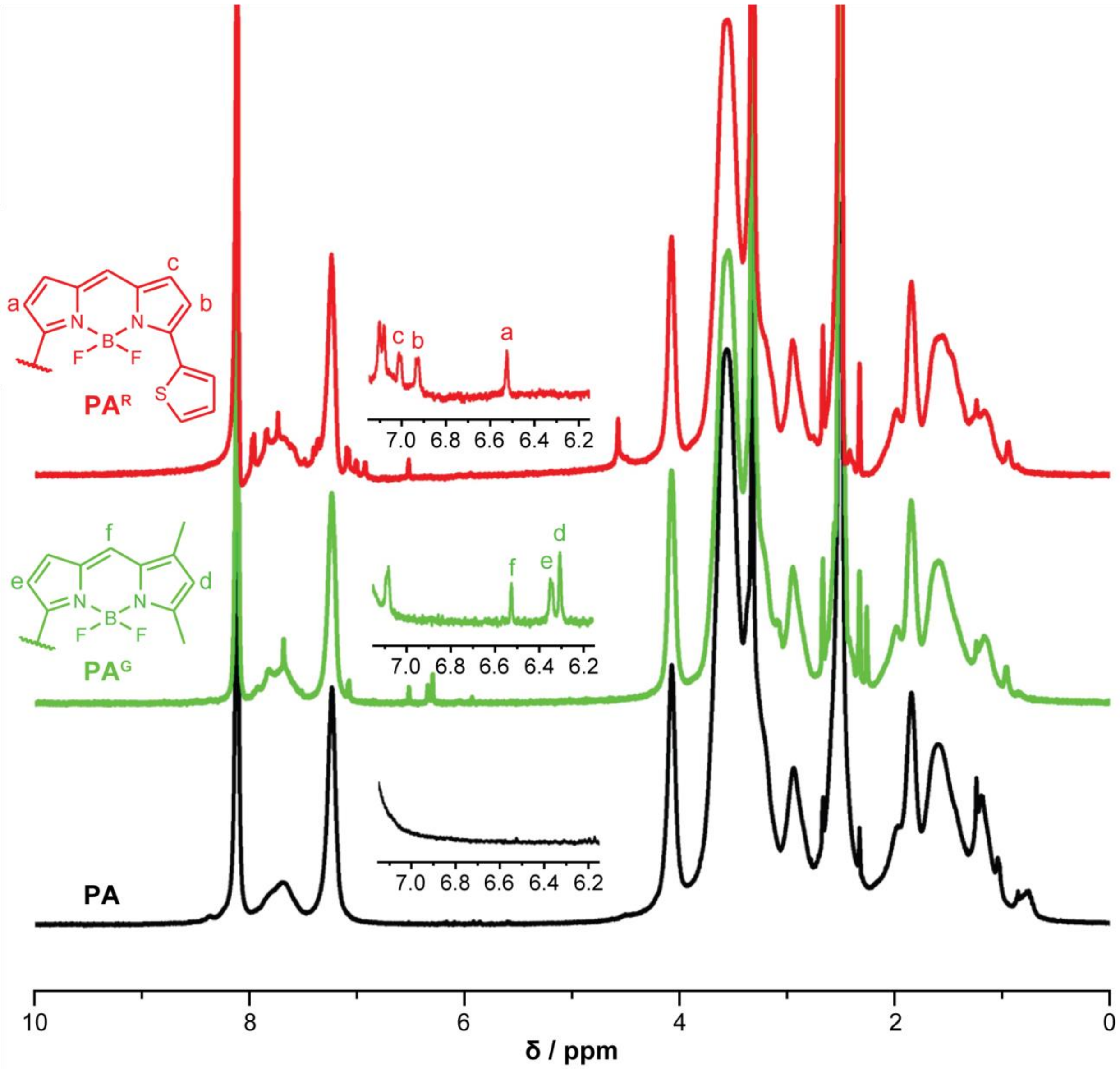

Supplementary Figure 34. ^{1}H NMR spectra of **PA**$^{\mathbf{G}}$ ((BODIPY-FL)-PNAM$_{39}$-*b*-PAAm$_{20}$), **PA**$^{\mathbf{R}}$ ((BODIPY-TR)-PNAM$_{39}$-*b*-PAAm$_{20}$) and **PA** (400 MHz, d_6-DMSO).

Stepwise Growth of Fluorescent Wormlike Nanoparticles

Fluorescent wormlike nanoparticles were fabricated as described in the main paper (Figure 4). Further control experiments are presented in Supplementary Figure 35, which demonstrate that colocalisation of the dyes was only observed when worms were grown with sequential additions of **PA**$^{\mathbf{G}}$ and **PA**$^{\mathbf{R}}$, and not when solutions of pure red and green worms were physically mixed.

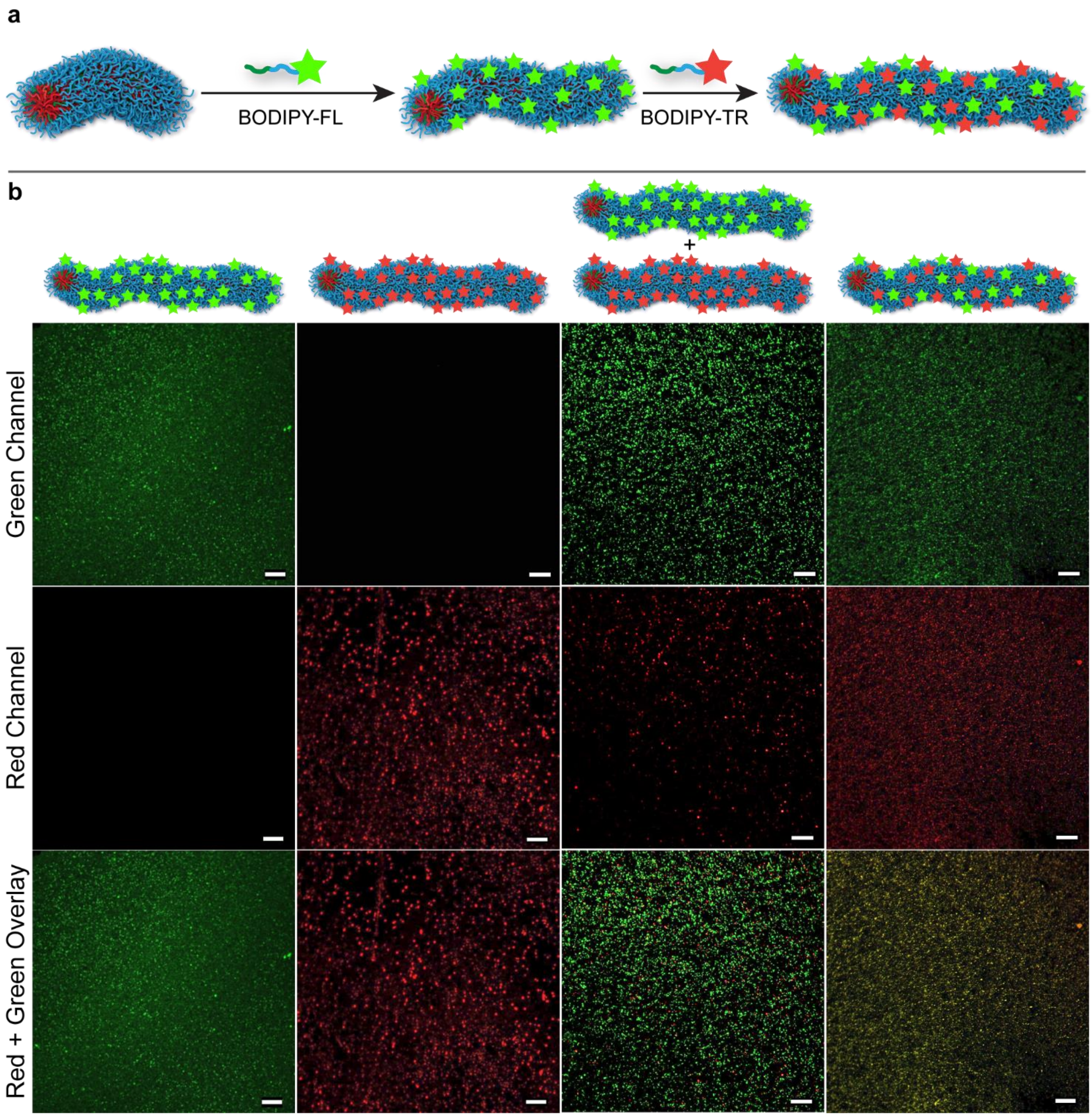

Supplementary Figure 35. Further control experiments for the controlled fabrication of fluorescent nanoparticles mediated by complementary H-bonding interactions. (a) Schematic overview showing the sequential addition of **PA** tagged with BODIPY-FL (**PA**$^{\mathbf{G}}$) to generate green fluorescent worms, followed by addition of **PA** tagged with BODIPY-TR (**PA**$^{\mathbf{R}}$) to generate yellow fluorescent worms. (b) Confocal miscopy images of different possible combinations of dye additions. 1st column: 2 sequential additions of **PA**$^{\mathbf{G}}$, showing only green particles. 2nd column: 2 sequential additions of **PA**$^{\mathbf{R}}$, showing only red particles. 3rd column: physical mixture of the particles shown in columns 1 and 2, showing discrete red and green particles. 4th column:

Sequential addition of **PA**$^{\mathbf{G}}$ followed by **PA**$^{\mathbf{R}}$, showing almost complete overlay of the red and green fluorescence, confirming colocalization. Scale bars = 10 μm.

Physical Model for MORPH

This subsection contains the technical details related to the physical model for MORPH presented in the main text. The focus is on relevant timescales in the experimental nanoparticle system, comparison with other polymer systems that tend to relax to equilibrium spherical micelles, and on formulating a more complex mathematical model that takes into account both nanoparticle shape and volume, and which reduces to the equation for eccentricity presented in the main text.

Relevant Timescales

There are many timescales relevant to the formation and growth of the nanoparticles. In the main text, we emphasize the timescales τ_I for insertion of a polymer into the nanoparticle core and timescale τ_R for the rearrangement and relaxation of the nanoparticle core chains. We assume τ_I is inversely proportional to the concentration of the polymer (i.e., more polymer will lead to faster insertion). We assume that τ_R will be determined principally by the bulk properties of the core chains (i.e., the bulk modulus and viscosity) and remain more or less independent of the polymer concentration. These timescales are microscopic, because they arise from the properties of individual polymer chains and chain–chain interactions.

A different approach to the dynamics is a phenomenological model with timescales for the growth of surface area and volume. The surface-area growth proceeds via timescale τ_A (and, intuitively, surface-area growth should depend strongly on the insertion timescale τ_I). The volume growth proceeds via a different timescale τ_V, which depends on the relaxation time τ_R.

Comparison with Equilibrium Phenomena

In addition to these timescales involved in the MORPH process, there are several timescales that describe equilibrium processes and which, in general, enter the physical description for an equilibrium micelle system. However, due to the non-equilibrium nature of the nanoparticles considered in this work, especially the glassiness of the nanoparticle core, these equilibrium timescales are too long to effect the morphological change from spheres to elongated nanoparticles that we observe.

In most diblock copolymer systems that form micelles, the dominant relaxation process for approaching equilibrium is single-chain extrusion (see, e.g., Ref. [7]). In this process, a micelle loses a single chain to the solution, and this chain diffuses and can rejoin a different micelle. This process leads to a broad equilibrium distribution of particle sizes. However, in the system we consider, single-chain extrusion is strongly suppressed by a combination of the glassiness of the nanoparticle core and the reversible H-bonding interactions between thymine and adenine (which are also not sufficiently strong for **PA** to pull the core chains out into the solution). As a result, polymers from the core do not individually leave the nanoparticle. Instead, any reformation into smaller nanoparticles must proceed via the budding and break-up of parts of the larger nanoparticles. This budding process is energetically costly and not observed during the shape-change step of the process. Instead, budding and break-up is only observed when these processes are strongly driven by the need to accommodate more polymers, i.e., at the end of the process in Figure 6 when the nanoparticle shell can no longer expand to accommodate more polymer insertion. Another potential disassembly process is a collective modulational instability of a cylinder, described in Refs. [8,9]. This process is expected to be as slow as the budding of individual smaller nanoparticles, and may be responsible for the cylinder-to-small-sphere transformation shown in the final stages of Figure 6.

Finally, surface tension of the core-solute interface is responsible for the (viscous) relaxation from anisotropic shapes into spherical nanoparticles. In equilibrium, this process is responsible for the

generic spherical shapes of many micelle systems. This relaxation process depends on the relaxation rate of the core. We believe that in our system, the suppression of this process through glassy core dynamics is essential for stabilizing a worm-like nanoparticle shape.

Swelling Dynamics

In this subsection, we proceed to derive the quantitative relations between the microscopic timescales (τ_R, τ_I) and macroscopic timescales (τ_A, τ_V). We demonstrate the importance of having a thin shell for the MORPH mechanism: the shell thickness effectively rescales the relaxation rate. As a starting point, consider the equations for the growth of nanoparticle volume V and surface area A:

$$\partial_t A = ({\tau_A}^{-1})A,\ \ \partial_t V = ({\tau_V}^{-1})aR^2 \qquad \text{(Supplementary Equation 24)}$$

where

$$A \sim (1+\epsilon^2)R^2 \qquad \text{(Supplementary Equation 25)}$$

ϵ is the eccentricity of the spheroidal shape,

$$V \sim R^3 \qquad \text{(Supplementary Equation 26)}$$

R is the nanoparticle radius, and a is the core thickness. We assume that the volume growth occurs primarily inside a shell of thickness a much smaller than particle radius R (although the case $a \sim R$ can be considered for shells of thickness comparable to the nanoparticle size).

The timescale τ_A for area growth depends on the core surface tension: the larger the surface tension, the slower the area growth rate. In the absence of polymer insertion, the nanoparticle will prefer a spherical shape that minimizes the core-solution interfacial area and the area growth rate at fixed volume would be negative, driven entirely by surface tension. More generally, the surface tension of the core favours shapes that are more isotropic and can present a barrier to the development of anisotropy.

MORPH Dynamics

The phenomenological model that we present for MORPH dynamics results from putting the above ingredients together. The equation for the evolution of the area can be rewritten as

$$R^2\,\partial_{\mathrm{t}}\epsilon^2 + 2\,R\,\partial_{\mathrm{t}}R = (\tau_{\mathrm{A}}{}^{-1})R^2 \qquad \text{(Supplementary Equation 27)}$$

to lowest order in ϵ^2, whereas the equation for the volume will take the form

$$\partial_t R = (\tau_{\mathrm{V}}{}^{-1})a/3 \qquad \text{(Supplementary Equation 28)}$$

Substituting the equation for $\partial_{\mathrm{t}}R$ into the equation for $\partial_{\mathrm{t}}\epsilon^2$, we obtain the dynamics introduced in the main text:

$$\partial_{\mathrm{t}}\epsilon^2 = [\tau_{\mathrm{A}}{}^{-1} - \tau_{\mathrm{V}}{}^{-1}a/(3R)] \qquad \text{(Supplementary Equation 29)}$$

where we identify

$$\tau_{\mathrm{I}}{}^{-1} = \tau_{\mathrm{A}}{}^{-1} \qquad \text{(Supplementary Equation 30)}$$

and

$$\tau_{\mathrm{R}}{}^{-1} = \tau_{\mathrm{V}}{}^{-1}a/(3R) \quad \text{(Supplementary Equation 31)}$$

The nonlinear terms ignored here can have two effects: stabilizing a dumbbell shape, and (if odd in ϵ) establishing a preference for elongated (prolate) over squished, pancake-like (oblate) shapes.

Glassiness of the core guarantees a slow relaxation time, so the transition from $\tau_{\mathrm{I}}{}^{-1} < \tau_{\mathrm{R}}{}^{-1}$ ($\epsilon = 0$) to $\tau_{\mathrm{I}}{}^{-1} > \tau_{\mathrm{R}}{}^{-1}$ ($\epsilon \neq 0$) can be realized. The relation given in $\tau_{\mathrm{R}}{}^{-1} = \tau_{\mathrm{V}}{}^{-1}a/(3R)$ (Supplementary Equation 31 shows the importance of having a thin shell (achieved through a short length of the added polymer core block): the ratio of shell thickness a to nanoparticle radius R rescales the effective relaxation rate that enters the equation for $\partial_{\mathrm{t}}\epsilon^2$: the anisotropic regime is easier to probe with a thinner shell.

One simple generalisation of the model that may better reflect the complexity of the polymer system is that the polymer insertion and relaxation timescales may be coupled due to many-body effects. As a result, the eccentricity equation may take the more general form,

$$\partial_t \epsilon^2 = \tau_I^{-\alpha} \tau_R^{-\beta} \left[\tau_I^{-1+\alpha+\beta} - \tau_R^{-1+\alpha+\beta} \right] \qquad \text{(Supplementary Equation 32)}$$

with exponents α and β satisfying

$$\alpha + \beta < 1 \qquad \text{(Supplementary Equation 33)}$$

Although this form changes the dynamical scaling, this more general form leaves the phenomenology of this mechanism intact, i.e., similar development of anisotropic behaviour may be observed in systems with quite different rates of insertion and core relaxation.

In this subsection, we have considered the instability from a spherically symmetric seed to an anisotropic ellipsoid (or worm). On the other side of the instability, once an anisotropic particle has been formed, other effects may play a significant role in the dynamics of further growth. For example, the polymers might exhibit preferential insertion from solution into the nanoparticle based on local surface curvature. In addition, if the inserted polymers diffuse along the surface, the diffusion dynamics could also depend on local curvature, leading to an uneven distribution of polymers on the surface. However, our evidence leads us to conclude that the driving force behind the formation and growth of anisotropic particles is the need to incorporate more surface area at fixed volume: if preferential insertion based on surface curvature was the key mechanism, an anisotropic seed would be necessary for anisotropic growth, whereas our seeds start out spherically symmetric.

SUPPLEMENTARY REFERENCES

1. Hua, Z. *et al.* Micellar nanoparticles with tuneable morphologies through interactions between nucleobase-containing synthetic polymers in aqueous solution. *Polym. Chem.* **7**, 4254–4262 (2016).

2. Andersson, M., Wittgren, B. & Wahlund, K. Accuracy in Multiangle Light Scattering Measurements for Molar Mass and Radius Estimations. Model Calculations and Experiments. *Anal. Chem.* **75**, 4279–4291 (2003).

3. Soetaert, K. & Petzoldt, T. Inverse Modelling, Sensitivity and Monte Carlo Analysis in R Using Package FME. *J. Stat. Softw.* **33**, 1–28 (2010).

4. Patterson, J. P. *et al.* A simple approach to characterizing block copolymer assemblies: graphene oxide supports for high contrast multi-technique imaging. *Soft Matter* **8**, 3322–3328 (2012).

5. Schärtl, W. *Light Scattering from Polymer Solutions and Nanoparticle Dispersions*. (Springer-Verlag Berlin Heidelberg, 2007). doi:10.1007/978-3-540-71951-9

6. Pedersen, J. S. Analysis of small-angle scattering data from colloids and polymer solutions: modeling and least-squares fitting. *Adv. Colloid Interface Sci.* **70**, 171–210 (1997).

7. Halperin, A. & Alexander, S. Polymeric Micelles: Their Relaxation Kinetics. *Macromolecules* **22**, 2403–2412 (1989).

8. Grason, G. M. & Santangelo, C. D. Undulated cylinders of charged diblock copolymers. *Eur. Phys. J. E* **20**, 335–346 (2006).

9. Lund, R., Willner, L., Richter, D., Lindner, P. & Narayanan, T. Kinetic Pathway of the Cylinder-to-Sphere Transition in Block Copolymer Micelles Observed in Situ by Time-Resolved Neutron and Synchrotron Scattering. *ACS Macro Lett.* **2**, 1082–1087 (2013).
